# Design, Synthesis, and Biological Evaluation of Novel Thiazolidinone-Containing Quinoxaline-1,4-di-*N*-oxides as Antimycobacterial and Antifungal Agents

**DOI:** 10.3389/fchem.2020.00598

**Published:** 2020-08-06

**Authors:** Heying Zhang, Jie Zhang, Wei Qu, Shuyu Xie, Lingli Huang, Dongmei Chen, Yanfei Tao, Zhenli Liu, Yuanhu Pan, Zonghui Yuan

**Affiliations:** ^1^National Reference Laboratory of Veterinary Drug Residues, MOA Key Laboratory for Detection of Veterinary Drug Residues, Huazhong Agricultural University, Wuhan, China; ^2^MOA Laboratory for Risk Assessment of Quality and Safety of Livestock and Poultry Products, Huazhong Agricultural University, Wuhan, China

**Keywords:** quinoxaline-1,4-di-*N*-oxides, thiazolidinone, antimycobacterial, antifungal, CoMFA, CoMSIA

## Abstract

Tuberculosis and fungal infections can pose serious threats to human health. In order to find novel antimicrobial agents, 26 novel quinoxaline-1,4-di-*N*-oxides containing a thiazolidinone moiety were designed and synthesized, and their antimycobacterial activities were evaluated. Among them, compounds **2t**, **2u**, **2y**, and **2z** displayed the most potent antimycobacterial activity against *Mycobacterium tuberculosis* strain H37Rv (minimal inhibitory concentration [MIC] = 1.56 μg/mL). The antifungal activity of all the compounds was also evaluated against *Candida albicans, Candida tropicalis, Aspergillus fumigatus*, and *Cryptococcus neoformans*. Compounds **2t**, **2u**, **2y**, and **2z** exhibited potential antifungal activities, with an MIC between 2 and 4 μg/mL. Comparative molecular field analysis (CoMFA: *q*^2^ = 0.914, *r*^2^ = 0.967) and comparative molecular similarity index analysis (CoMSIA: *q*^2^ = 0.918, *r*^2^ = 0.968) models were established to investigate the structure and antimycobacterial activity relationship. The results of contour maps revealed that electronegative and sterically bulky substituents play an important role in the antimycobacterial activity. Electronegative and sterically bulky substituents are preferred at the C7 position of the quinoxaline ring and the C4 position of the phenyl group to increase the antimycobacterial activity. Additionally, more hydrogen bond donor substituents should be considered at the C2 side chain of the quinoxaline ring to improve the activity.

## Introduction

Tuberculosis (TB) is a highly dreaded contagious and worrisome disease caused by *Mycobacterium tuberculosis*; it is the second leading cause of worldwide death among infectious diseases. Due to its high infectivity and mortality, a third of the world population is affected (Shinnick et al., 1995; Glaziou et al., 2009, 2018). Similarly, the incidence of fungal infections has gradually increased over the past two decades and poses a serious threat to human health (Brown et al., 2012; Castelli et al., 2014; Zhao et al., 2017). However, due to the irrational and even abusive use of antimicrobial agents, there has been a rapid emergence of bacterial resistance and the spread and promotion of pathogenic microorganisms. These phenomena highlight the need for the development of novel antimicrobial agents (Wise, 2011).

Quinoxaline-1,4-di-*N*-oxides are a class of compounds that possess very interesting biological properties (Carta et al., 2006; Gonzalez and Cerecetto, 2012), such as antibacterial (Ishikawa et al., 2013), anticancer (Mielcke et al., 2012; Rajule et al., 2012), antiviral (Wilhelmsson et al., 2008), antimycobacterial (Wilhelmsson et al., 2008; Ancizu et al., 2010; Keri et al., 2018), and antifungal (Tandon et al., 2006) actions. Villar et al. explored quinoxaline-1,4-di-*N*-oxides as antimycobacterial agents. Some of the reported compounds displayed good antimycobacterial activity against resistant strains; 3-methyl-2-(2′-methylphenyl aminocarbonyl)-quinoxaline-1,4-di-*N*-oxide exhibited the most potent antimycobacterial activity against an isoniazid-resistant strain (Villar et al., 2008). Similarly, other authors have reported antimycobacterial activity for quinoxaline-2-carboxylate and quinoxaline-2-carbonitrile derivatives. The compound 7-methyl-3-(4′-fluoro)phenylquinoxaline-2-carbonitrile-1,4-di-*N*-oxide showed good antimycobacterial activity against *M. tuberculosis* (*Mtb*) strain H37Rv (minimal inhibitory concentration [MIC] < 0.2 μg/mL and selectivity index [SI] > 500) (Ortega et al., 2001; Vicente et al., 2009). Further, using molecular hybridization, a group designed and prepared a series of quinoxaline-1,4-di-*N*-oxides containing isoniazid (INH) and quinoxaline-1,4-di-*N*-oxide derivatives and evaluated their *in vitro* antimycobacterial activity against *Mtb* strain H37Rv. Some of the compounds displayed good antimycobacterial activity, with median inhibitory concentration (IC_50_) values between 0.58 and 1.50 μM (Torres et al., 2011).

Thiazolidinone derivatives also have wide biological activities and pharmacological properties (Kaur et al., 2017). This interesting core has received considerable attention for its myriad biological activities, such as antibacterial (Palekar et al., 2009; Desai et al., 2013), antidiabetic (Hussain et al., 2019), antibiofilm (Pan et al., 2010), anticancer (Liu et al., 2012), antifungal (Dandia et al., 2006; Desai et al., 2013), anti-inflammatory (Abdellatif et al., 2016), tyrosinase inhibitory (Ha et al., 2012), cyclooxygenase-2 inhibitory (Abdellatif et al., 2016), and anti-HIV (Rawal et al., 2005, 2007) properties. The investigation of the biological activity of thiazolidinones has revealed that substitution at different positions will produce distinct activities.

**Figure 1:**
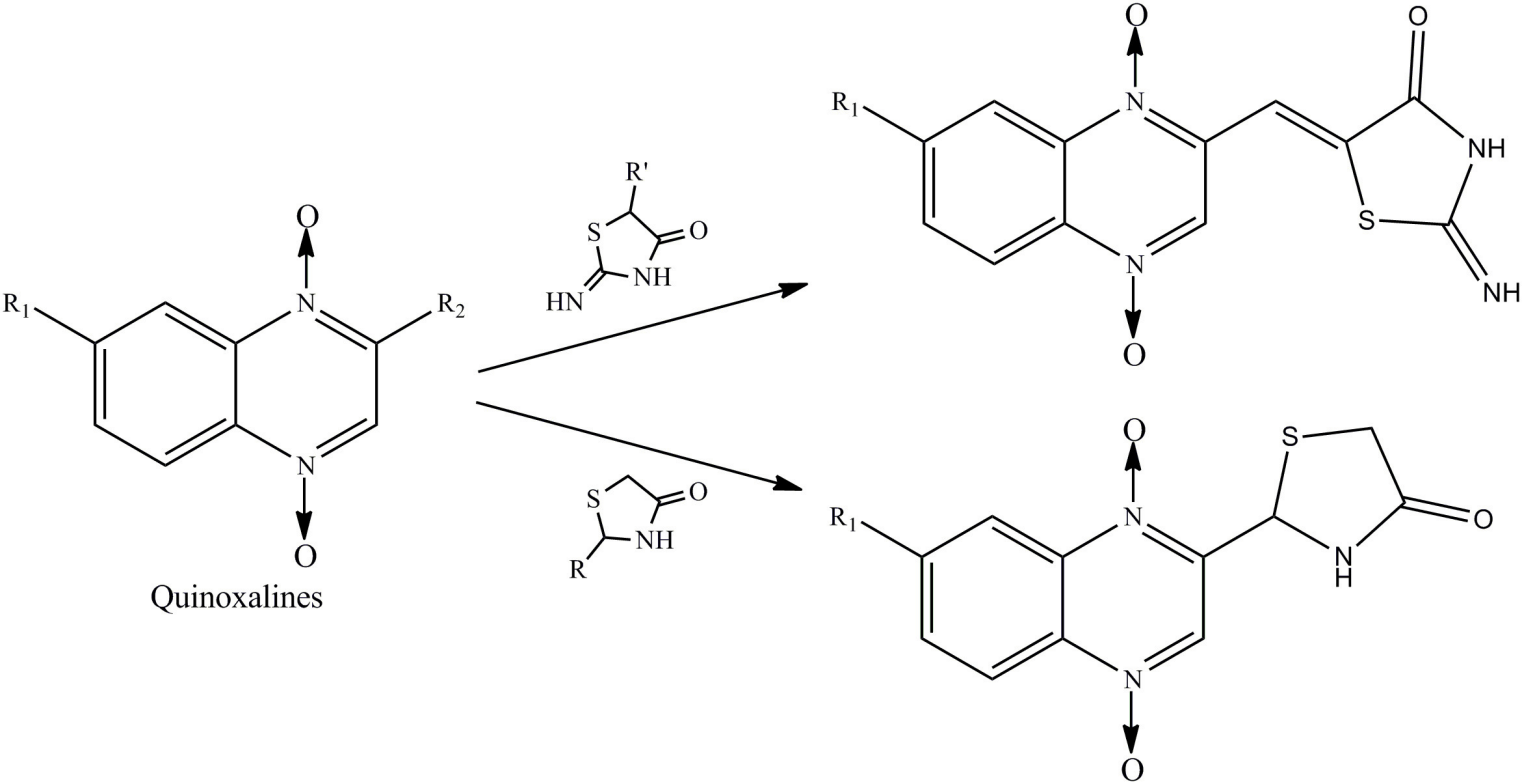
Hybridization design of quinoxaline-1,4-di-*N*-oxides containing a thiazolidinone moiety.

Therefore, the aforementioned compounds have inspired us to attach substituted thiazolidinones to the C2 side chain of the quinoxaline ring, and the combination of these two specific structures in one molecule leads to interesting antimycobacterial and antifungal activities. As a continuation of our research program on quinoxaline-1,4-di-*N*-oxides with antibacterial activity (Pan et al., 2016), here we report the synthesis of novel quinoxaline-1,4-di-*N*-oxides with substituted thiazolidinones attached to the C2 position of the quinoxaline ring (Figure 1). We evaluated their antimycobacterial activities against *Mtb* strain H37Rv and their antifungal activity against *Candida albicans, Candida tropicalis, Aspergillus fumigatus*, and *Cryptococcus neoformans*. Additionally, we preliminarily described the structure–antimycobacterial activity relationships (SAR) by 3D-QSAR models: comparative molecular field analysis (CoMFA) and comparative molecular similarity index analysis (CoMSIA).

## Materials and Methods

### Chemicals and Drugs

All of the chemicals were commercially available and procured from companies like Aldrich, Merck, and Aladdin. All reactants and solvents were analytically pure and were used without further purification. The starting compounds, 4-substituted-2-nitroaniline (**I**), were purchased from Aladdin. All of the positive control (rifampicin, amphotericin b, and ketoconazole) were purchased from the National Institutes of Food and Drug Control.

### Instruments

The nuclear magnetic resonance (NMR) spectra were measured on a Bruker Biospin AV400 instrument at 400 MHz for ^1^H NMR spectra and 100 MHz for ^13^C NMR spectra, with deuterated dimethylsulfoxide (DMSO-*d6*) as the solvent. The high-resolution mass spectra were acquired with a Q-TOF mass spectrometer (Impact II, Bruker). Thin-layer chromatography (TLC) was performed on silica gel plates (silica gel GF254, Qingdao Marine Chemical Plant, China) visualized at 254 nm. The derivatives were purified by flash column chromatography in silica gel (particle size 200–300 mesh) to get the pure compounds. 3D-QSAR procedures were carried out using SYBYL-X 2.0 (Tripos, Inc.). A multimode reader (Infinite M200, Tecan) was used to detect the cytotoxicity.

### Chemistry

**Scheme 1:**
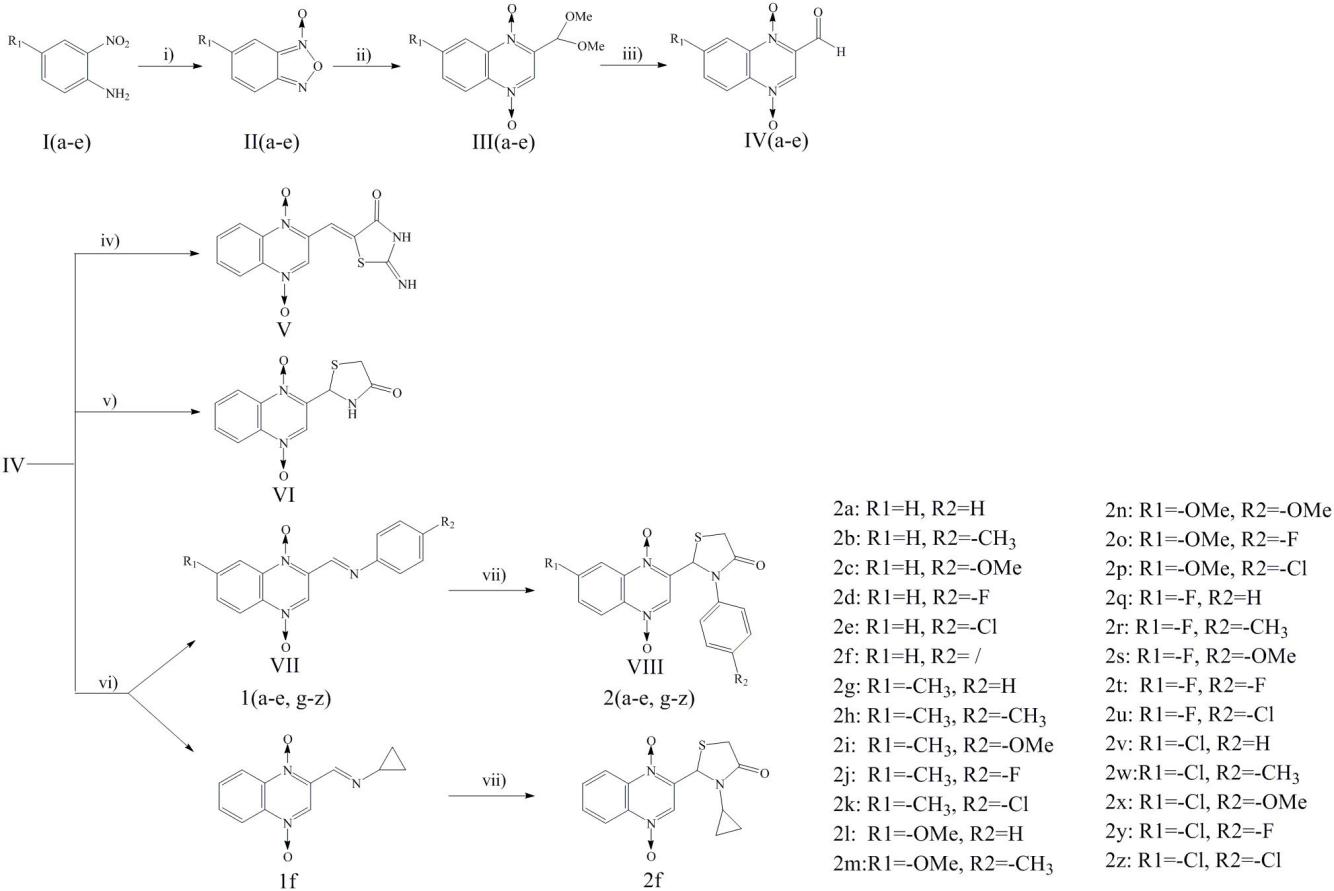
Reagents and conditions: (i) THF, NaOH, NaClO, 0°C, 2 h; (ii) DMF, THP, Pyruvic aldehyde dimethyl acetal, 45°C, 24 h; (iii) HCl, 60°C, 12 h; (iv) 2-iminothiazolidin-4-one, ethanol, ammonium acetate, glacial acetic acid, reflux, 24 h; (v) Toluene, thioglycollic acid, ammonium carbonate, reflux, 18 h; (vi) Methanol, aromatic amine/cyclopropylamine, rt, 30 min; (vii) Thioglycollic acid, toluene, 85°C, reflux, 8 h.

Quinoxaline-1,4-di-*N*-oxide derivatives were synthesized according to Scheme 1. These compounds were prepared by reaction of substituted-2-quinoxalinecarboxaldehyde-1, 4-di-*N*-oxide (**IV**) with 2-iminothiazolidin-4-one, thioglycollic acid, or aromatic amine/cyclopropylamine in one or two steps. Detailed reaction conditions are available in the general procedures.

#### General Procedure for the Synthesis of Substituted-Benzofuroxan (II)

To a stirred suspension of substituted-2-nitroaniline **(I)** (0.05 mol) in THF (75 mL), sodium hydroxide (0.015 mol, 0.6 g) was added as the catalyst, followed by sodium hypochlorite (240 mL) at 0°C. The reaction mixture was stirred at 0°C for 2 h. The reaction mixture was diluted with water and extracted with dichloromethane three times; subsequently, the combined organic layer was washed with water. The organic layer was dried over Na_2_SO_4_ and evaporated under reduced pressure to produce a crude solid, substituted-benzofuroxan **(II)**, that was pure enough to proceed to the next steps without further purification (Ortega et al., 2002; Zhao et al., 2016).

#### General Procedure for the Synthesis of Substituted-2- (dimethoxymethyl)-quinoxaline-1, 4-di-*N*-oxide (III)

The pyruvic aldehyde dimethyl acetal (0.15 mol, 17.7 g) was added to a solution of the substituted-benzofuroxan **(II)** (0.1 mol) in DMF. Pyrrolidine (0.05 mol, 3.6 g) was then added dropwise as the catalyst, and the mixture was stirred at 45°C for 24 h. The reaction mixture was cooled and then poured into ice water, and a yellow solid was obtained. The solid was washed by adding freezer-cold EtOH to afford substituted-2- (dimethoxymethyl) - quinoxaline-1, 4-di-*N*-oxide **(III)** (Haddadin and Issidorides, 1993; Zarranz et al., 2005).

#### General Procedure for the Synthesis of Substituted-2-quinoxalinecarboxaldehyde-1, 4-di-*N*-oxide (IV)

The substituted-2- (dimethoxymethyl) -quinoxaline-1, 4-di-*N*-oxide **(III)** (0.1 mol) was dissolved in 6M HCl (150 mL), and the mixture was stirred at 60°C for 12 h. The saturated NaHCO_3_ solution was then added drop by drop to adjust the pH value of the reaction mixture to 7.5, after which the reaction mixture was incubated at 4°C for 2 h. A pale yellow solid was obtained, filtered off, and washed by adding freezer-cold EtOH to afford substituted-2-quinoxalinecarboxaldehyde-1, 4-di-*N*-oxide **(IV)**.

#### General Procedure for the Synthesis of 2-iminothiazolidin-4-one (3)

A mixture of thiourea (0.05 mol, 3.8 g), 2-chloroacetic acid (0.05 mol, 4.7 g), and glacial acetic acid (20 ml) was refluxed for 1 h. The white precipitate, 2-iminothiazolidin-4-one **(3)**, was collected and washed with methanol. It was pure enough to proceed to the next steps without further purification (Ghareb et al., 2017).

#### General Procedure for the Synthesis of 2-(2′-iminothiazolidin-4′-one)-quinoxaline-1, 4-di-*N*-oxide (V)

To a solution of 2-iminothiazolidin-4-one **(3)** (0.01 mol, 1.2 g) in ethanol, equimolar amounts of substituted-2-quinoxalinecarboxaldehyde-1, 4-di-*N*-oxide **(IV)**, ammonium acetate, and glacial acetic acid (5 mL) were added. The resulting mixture was refluxed for ~24 h. Cooling the mixture to room temperature provided the targeted compound 2-(2'-iminothiazolidin-4'-one)-quinoxaline-1, 4-di-*N*-oxide **(V)**, which was then filtered, washed, dried, and recrystallized from hot ethanol (Jadav et al., 2015).

#### General Procedure for the Synthesis of 2- (4'(5H)-thiazolone)-quinoxaline-1, 4-di-*N*-oxide (VI)

A mixture of 2-quinoxalinecarboxaldehyde-1, 4-di-*N*-oxide **(IV)** (0.01 mol), thioglycollic acid (0.015 mol, 1.4 g), and (NH_4_)_2_CO_3_ (0.05 mol, 4.8 g) in 200 mL toluene was heated under reflux for 12 h with stirring and collection of the generated water in a water separator. The mixture was cooled, washed with water, and evaporated under reduced pressure. The oily residue was purified by silica gel column chromatography to get 2- (4'(5H)-thiazolone)-quinoxaline-1, 4-di-*N*-oxide **(VI)** (El Nezhawy et al., 2009).

#### General Procedure for the Synthesis of Substituted-2-iminequinoxaline-1, 4-di-*N*-oxide (VII)

The substituted-2-quinoxalinecarboxaldehyde-1, 4-di-*N*-oxide **(IV)** (0.01 mol) was dissolved in methanol. Substituted-aromatic amine/cyclopropylamine (0.015 mol) was then added, and the reaction mixture was stirred at 25°C for 30 min. A yellow solid was obtained during the reaction process. The resulting yellow precipitate was filtered off, washed with freezer-cold EtOH, and dried. Affording the substituted-2-iminequinoxaline-1, 4-di-*N*-oxide **(VII)**.

#### General Procedure for the Synthesis of substituted-2-(3′-substituted-4′(5H)-thiazolone)-quinoxaline-1, 4-di-*N*-oxide (VIII)

A solution of substituted-2-iminequinoxaline-1, 4-di-*N*-oxide **(VII)** (0.01 mol) in toluene was heated at 85°C for 30 min. Thioglycollic acid (0.03 mol, 2.8 g) was then added to the solution, which was then refluxed for 8 h. Subsequently, the reaction mixture was evaporated under reduced pressure. Then, the crude product was purified by silica gel column chromatography to obtain substituted-2- (3'-substituted-4'(5H)-thiazolone)-quinoxaline-1, 4-di-*N*-oxide **(VIII)**.

### Product Characterization

#### 2- (3′-phenyl-4′(5H)-thiazolidinone)-quinoxaline-1, 4-di-*N*-oxide (2a)

Yellow crystals; yield: 55%; mp: 245–248°C; ^1^H NMR (400 MHz) δ 8.61 (s, 1H), 8.49 (dd, *J* = 8.4, 1.3 Hz, 1H), 8.38 (dd, *J* = 8.4, 1.4 Hz, 1H), 8.03–7.91 (m, 2H), 7.53 (d, *J* = 7.9 Hz, 2H), 7.38 (t, *J* = 7.9 Hz, 2H), 7.23 (t, *J* = 7.4 Hz, 1H), 6.83 (s, 1H), 4.14 (dd, *J* = 15.3, 1.4 Hz, 1H), 3.84 (d, *J* = 15.3 Hz, 1H); ^13^C NMR (100 MHz) δ 171.66, 141.74, 138.23, 137.75, 137.54, 133.12, 132.64, 129.70, 127.46, 125.13, 120.28, 119.90, 57.85, 33.32; MS (ESI) *m*/*z* 340 [M+H]^+^. HR-MS (ESI): calculated for C_17_H_13_N_3_O_3_S [M+Na]^+^ 362.0570; found: 362.0634.

#### 2- (3′-P-methylphenyl-4′(5H)-thiazolidinone)-quinoxaline-1, 4-di-*N*-oxide (2b)

Yellow crystals; yield: 43%; mp: 251–253°C; ^1^H NMR (400 MHz) δ 8.60 (s, 1H), 8.52–8.45 (m, 1H), 8.41–8.34 (m, 1H), 8.02–7.91 (m, 2H), 7.39 (d, *J* = 7.8 Hz, 2H), 7.17 (d, *J* = 8.3 Hz, 2H), 6.79 (s, 1H), 4.12 (dd, *J* = 15.2, 1.5 Hz, 1H), 3.82 (d, *J* = 15.2 Hz, 1H), 2.23 (s, 3H); ^13^C NMR (100 MHz) δ 171.55, 141.75, 138.23, 137.73, 136.95, 134.95, 133.12, 132.66, 130.16, 129.74, 125.07, 120.28, 119.90, 57.90, 33.34, 20.96; MS (ESI) *m*/*z* 354 [M+H]^+^. HR-MS (ESI): calculated for C_18_H_15_N_3_O_3_S [M+Na]^+^ 376.0726; found: 376.0735.

#### 2- (3′-P-methoxyphenyl-4′(5H)-thiazolidinone)-quinoxaline-1, 4-di-*N*-oxide (2c)

Yellow crystals; yield: 58%; mp: 255–257°C; ^1^H NMR (400 MHz) δ 8.63 (s, 1H), 8.49 (dd, *J* = 8.4, 1.3 Hz, 1H), 8.41–8.36 (m, 1H), 8.03–7.90 (m, 2H), 7.40 (d, *J* = 7.4 Hz, 2H), 6.94–6.87 (m, 2H), 6.74 (s, 1H), 4.10 (dd, *J* = 15.1, 1.6 Hz, 1H), 3.81 (d, *J* = 15.2 Hz, 1H), 3.70 (s, 3H); ^13^C NMR (100 MHz) δ 171.57, 158.37, 141.75, 138.25, 137.74, 133.13, 132.67, 130.10, 127.02, 120.30, 119.91, 114.86, 58.07, 55.69, 33.25; MS (ESI) *m*/*z* 370 [M+H]^+^. HR-MS (ESI): calculated for C_18_H_15_N_3_O_4_S [M+Na]^+^ 392.0675; found: 392.0672.

#### 2- (3′-P-fluorophenyl-4′(5H)-thiazolidinone)-quinoxaline-1, 4-di-*N*-oxide (2d)

Yellow crystals; yield: 51%; mp: 240–242°C; ^1^H NMR (400 MHz) δ 8.66 (s, 1H), 8.52–8.46 (m, 1H), 8.42–8.36 (m, 1H), 8.03–7.92 (m, 2H), 7.57 (dd, *J* = 8.1, 4.9 Hz, 2H), 7.26–7.17 (m, 2H), 6.81 (s, 1H), 4.12 (dd, *J* = 15.3, 1.5 Hz, 1H), 3.83 (d, *J* = 15.3 Hz, 1H); ^13^C NMR (100 MHz) δ 171.74, 162.02, 159.59, 141.59, 138.25, 137.81, 133.76, 133.73, 133.11, 132.65, 129.76, 127.74, 127.65, 120.30, 119.91, 116.63, 116.40, 57.92, 33.25; MS (ESI) *m*/*z* 358 [M+H]^+^. HR-MS (ESI): calculated for C_17_H_12_FN_3_O_3_S [M+Na]^+^ 380.0476; found: 380.0490.

#### 2- (3′-P-chlorophenyl-4′(5H)-thiazolidinone)-quinoxaline-1, 4-di-*N*-oxide (2e)

Yellow crystals; yield: 56%; mp: 249–252°C; ^1^H NMR (400 MHz) δ 8.63 (s, 1H), 8.52–8.45 (m, 1H), 8.43–8.34 (m, 1H), 8.03–7.91 (m, 2H), 7.58 (d, *J* = 8.8 Hz, 2H), 7.48–7.38 (m, 2H), 6.84 (s, 1H), 4.12 (dd, *J* = 15.4, 1.4 Hz, 1H), 3.84 (d, *J* = 15.4 Hz, 1H); ^13^C NMR (100 MHz) δ 171.71, 141.59, 138.25, 137.83, 136.40, 133.10, 132.65, 131.54, 129.64, 126.82, 120.31, 119.91, 57.58, 33.24; MS (ESI) *m*/*z* 374 [M+H]^+^. HR-MS (ESI): calculated for C_17_H_12_ClN_3_O_3_S [M+Na]^+^ 396.0180; found: 396.0153.

#### 2- (3′-cyclopropyl-4′(5H)-thiazolidinone)-quinoxaline-1, 4-di-*N*-oxide (2f)

Yellow crystals; yield: 48%; mp: 249–252°C; ^1^H NMR (400 MHz) δ 8.64 (s, 1H), 8.54–8.42 (m, 2H), 8.06–7.93 (m, 2H), 6.01 (d, *J* = 1.5 Hz, 1H), 3.90 (dd, *J* = 15.1, 1.3 Hz, 1H), 3.60 (d, *J* = 15.1 Hz, 1H), 2.46 (s, 1H), 0.88–0.66 (m, 3H), 0.62–0.47 (m, 1H); ^13^C NMR (100 MHz) δ 173.44, 141.94, 138.48, 137.94, 133.00, 132.55, 129.84, 120.33, 119.94, 57.55, 33.47, 26.44, 8.01, 4.79; MS (ESI) *m*/*z* 304 [M+H]^+^. HR-MS (ESI): calculated for C_14_H_13_N_3_O_3_S [M+H]^+^ 304.0750; found: 304.0731.

#### 7-CH_3_-2- (3′-phenyl-4′(5H)-thiazolidinone)-quinoxaline-1, 4-di-**N**-oxide (2g)

Yellow crystals; yield: 43%; mp: 255–257°C; ^1^H NMR (400 MHz) δ 8.57 (s, 1H), 8.36 (d, *J* = 8.8 Hz, 1H), 8.17 (s, 1H), 7.80 (dd, *J* = 8.9, 1.7 Hz, 1H), 7.51 (d, *J* = 7.8 Hz, 2H), 7.37 (t, *J* = 7.9 Hz, 2H), 7.22 (dd, *J* = 10.6, 4.2 Hz, 1H), 6.81 (s, 1H), 4.13 (dd, *J* = 15.2, 1.4 Hz, 1H), 3.83 (d, *J* = 15.3 Hz, 1H), 2.54 (s, 3H); ^13^C NMR (100 MHz) δ 171.65, 143.73, 140.92, 137.55, 136.60, 134.82, 129.70, 127.48, 125.19, 119.67, 119.10, 57.82, 33.29, 21.63; MS (ESI) *m*/*z* 354 [M+H]^+^. HR-MS (ESI): calculated for C_18_H_15_N_3_O_3_S [M+Na]^+^ 376.0726; found: 376.0777.

#### 7-CH_3_-2- (3′-P-methylphenyl-4′(5H)-thiazolidinone)-quinoxaline-1, 4-di-*N*-oxide (2h)

Yellow crystals; yield: 47%; mp: 257–259°C; ^1^H NMR (400 MHz) δ 8.56 (s, 1H), 8.36 (d, *J* = 8.8 Hz, 1H), 8.17 (s, 1H), 7.80 (dd, *J* = 9.0, 1.7 Hz, 1H), 7.37 (d, *J* = 7.4 Hz, 2H), 7.16 (d, *J* = 8.3 Hz, 2H), 6.77 (s, 1H), 4.11 (dd, *J* = 15.2, 1.4 Hz, 1H), 3.81 (d, *J* = 15.2 Hz, 1H), 2.54 (s, 3H), 2.22 (s, 3H); ^13^C NMR (100 MHz) δ 171.55, 143.74, 140.98, 137.53, 136.97, 136.60, 134.95, 134.82, 130.16, 129.81, 125.12, 119.67, 119.10, 57.93, 33.33, 21.63, 20.96; MS (ESI) *m*/*z* 368 [M+H]^+^. HR-MS (ESI): calculated for C_19_H_17_N_3_O_3_S [M+H]^+^ 368.1063; found: 368.1035.

#### 7-CH_3_-2- (3′-P-methoxyphenyl-4′(5H)-thiazolidinone)-quinoxaline-1, 4-di-*N*-oxide (2i)

Yellow crystals; yield: 42%; mp: 255–259°C; ^1^H NMR (400 MHz) δ 8.59 (s, 1H), 8.36 (d, *J* = 8.8 Hz, 1H), 8.18 (s, 1H), 7.80 (dd, *J* = 8.9, 1.6 Hz, 1H), 7.38 (d, *J* = 5.5 Hz, 2H), 6.91 (d, *J* = 9.0 Hz, 2H), 6.72 (s, 1H), 4.10 (dd, *J* = 15.1, 1.4 Hz, 1H), 3.80 (d, *J* = 15.1 Hz, 1H), 3.69 (s, 3H), 2.55 (s, 3H); ^13^C NMR (100 MHz) δ 171.57, 158.38, 143.74, 140.86, 137.53, 136.61, 134.81, 130.10, 127.06, 119.68, 119.11, 114.85, 58.18, 55.68, 33.32, 21.63; MS (ESI) *m*/*z* 384 [M+H]^+^. HR-MS (ESI): calculated for C_19_H_17_N_3_O_4_S [M+H]^+^ 384.1013; found: 384.0952.

#### 7-CH_3_-2- (3′-P-fluorophenyl-4′(5H)-thiazolidinone)-quinoxaline-1, 4-di-*N*-oxide (2j)

Yellow crystals; yield: 48%; mp: 243–245°C; ^1^H NMR (400 MHz) δ 8.61 (s, 1H), 8.37 (d, *J* = 8.8 Hz, 1H), 8.19 (s, 1H), 7.81 (dd, *J* = 8.9, 1.6 Hz, 1H), 7.54 (d, *J* = 7.6 Hz, 2H), 7.25–7.17 (m, 2H), 6.78 (s, 1H), 4.10 (dd, *J* = 15.2, 1.4 Hz, 1H), 3.82 (d, *J* = 15.3 Hz, 1H), 2.56 (s, 3H); ^13^C NMR (100 MHz) δ 171.74, 162.04, 159.61, 143.76, 140.77, 137.62, 136.63, 134.84, 133.76, 133.74, 129.84, 127.79, 127.70, 119.70, 119.13, 116.64, 116.41, 57.91, 33.24, 21.64; MS (ESI) *m*/*z* 372 [M+H]^+^. HR-MS (ESI): calculated for C_18_H_14_FN_3_O_3_S [M+H]^+^ 372.0813; found: 372.0784.

#### 7-CH_3_-2- (3′-P-chlorophenyl-4′(5H)-thiazolidinone)-quinoxaline-1, 4-di-*N*-oxide (2k)

Yellow crystals; yield: 52%; mp: 244–248°C; ^1^H NMR (400 MHz) δ 8.59 (s, 1H), 8.36 (d, *J* = 8.8 Hz, 1H), 8.18 (s, 1H), 7.80 (dd, *J* = 8.9, 1.7 Hz, 1H), 7.57 (d, *J* = 8.7 Hz, 2H), 7.46–7.40 (m, 2H), 6.83 (s, 1H), 4.12 (dd, *J* = 15.3, 1.4 Hz, 1H), 3.83 (d, *J* = 15.3 Hz, 1H), 2.55 (s, 3H); ^13^C NMR (100 MHz) δ 171.71, 143.73, 140.76, 137.61, 136.60, 136.40, 134.79, 131.57, 129.64, 126.87, 119.67, 119.11, 57.59, 33.27, 21.63; MS (ESI) *m*/*z* 388 [M+H]^+^. HR-MS (ESI): calculated for C_18_H_14_ClN_3_O_3_S [M+H]^+^ 388.0517; found: 388.0529.

#### 7-OMe-2- (3′-phenyl-4′(5H)-thiazolidinone)-quinoxaline-1, 4-di-*N*-oxide (2l)

Yellow crystals; yield: 55%; mp: 254–258°C; ^1^H NMR (400 MHz) δ 8.60 (s, 1H), 8.40 (d, *J* = 9.5 Hz, 1H), 7.68 (d, *J* = 2.7 Hz, 1H), 7.59 (dd, *J* = 9.5, 2.8 Hz, 1H), 7.48 (d, *J* = 7.8 Hz, 2H), 7.36 (t, *J* = 7.9 Hz, 2H), 7.22 (t, *J* = 7.4 Hz, 1H), 6.79 (s, 1H), 4.12 (dd, *J* = 15.2, 1.5 Hz, 1H), 3.96 (s, 3H), 3.82 (d, *J* = 15.2 Hz, 1H); ^13^C NMR (100 MHz) δ 171.63, 162.58, 140.57, 139.16, 137.55, 133.27, 129.71, 127.51, 125.23, 124.58, 121.71, 99.37, 57.85, 57.00, 33.28; MS (ESI) *m*/*z* 370 [M+H]^+^. HR-MS (ESI): calculated for C_18_H_15_N_3_O_4_S [M+Na]^+^ 392.0675; found: 392.0665.

#### 7-OMe-2- (3′-P-methylphenyl-4′(5H)-thiazolidinone)-quinoxaline-1, 4-di-*N*-oxide (2m)

Yellow crystals; yield: 53%; mp: 257–259°C; ^1^H NMR (400 MHz) δ 8.59 (s, 1H), 8.39 (d, *J* = 9.5 Hz, 1H), 7.67 (d, *J* = 2.7 Hz, 1H), 7.58 (dd, *J* = 9.5, 2.7 Hz, 1H), 7.36 (d, *J* = 7.3 Hz, 2H), 7.16 (d, *J* = 8.3 Hz, 2H), 6.76 (s, 1H), 4.11 (dd, *J* = 15.2, 1.5 Hz, 1H), 3.96 (s, 3H), 3.80 (d, *J* = 15.2 Hz, 1H), 2.22 (s, 3H); ^13^C NMR (100 MHz) δ 171.52, 162.55, 139.61, 139.12, 136.96, 134.96, 133.50, 130.15, 125.14, 124.53, 121.68, 99.34, 57.84, 56.99, 33.36, 20.97; MS (ESI) *m*/*z* 384 [M+H]^+^. HR-MS (ESI): calculated for C_19_H_17_N_3_O_4_S [M+H]^+^ 384.1013; found: 384.1034.

#### 7-OMe-2- (3′-P-methoxyphenyl-4′(5H)-thiazolidinone)-quinoxaline-1, 4-di-*N*-oxide (2n)

Yellow crystals; yield: 49%; mp: 258–260°C; ^1^H NMR (400 MHz) δ 8.62 (s, 1H), 8.39 (d, *J* = 9.5 Hz, 1H), 7.68 (d, *J* = 2.7 Hz, 1H), 7.58 (dd, *J* = 9.5, 2.8 Hz, 1H), 7.37 (d, *J* = 4.9 Hz, 2H), 6.90 (d, *J* = 9.0 Hz, 2H), 6.71 (s, 1H), 4.10 (dd, *J* = 15.1, 1.6 Hz, 1H), 3.96 (s, 3H), 3.80 (d, *J* = 15.1 Hz, 1H), 3.69 (s, 3H); ^13^C NMR (100 MHz) δ 171.54, 162.54, 158.38, 139.68, 139.12, 133.52, 130.11, 127.06, 124.51, 121.68, 114.84, 99.34, 58.04, 56.98, 55.68, 33.30; MS (ESI) *m*/*z* 400 [M+H]^+^. HR-MS (ESI): calculated for C_19_H_17_N_3_O_5_S [M+H]^+^ 400.0962; found: 400.0915.

#### 7-OMe-2- (3′-P-fluorophenyl-4′(5H)-thiazolidinone)-quinoxaline-1, 4-di-*N*-oxide (2o)

Yellow crystals; yield: 57%; mp: 255–257°C; ^1^H NMR (400 MHz) δ 8.64 (s, 1H), 8.39 (d, *J* = 9.5 Hz, 1H), 7.69 (d, *J* = 2.7 Hz, 1H), 7.59 (dd, *J* = 9.5, 2.8 Hz, 1H), 7.52 (d, *J* = 4.7 Hz, 2H), 7.25–7.16 (m, 2H), 6.77 (s, 1H), 4.10 (dd, *J* = 15.2, 1.5 Hz, 1H), 3.96 (s, 3H), 3.82 (d, *J* = 15.2 Hz, 1H); ^13^C NMR (100 MHz) δ 171.71, 162.56, 162.03, 159.60, 140.54, 139.20, 133.78, 133.75, 133.53, 130.33, 127.81, 127.72, 124.53, 121.70, 116.63, 116.40, 99.36, 57.90, 57.00, 31.16; MS (ESI) *m*/*z* 388 [M+H]^+^. HR-MS (ESI): calculated for C_18_H_14_FN_3_O_4_S [M+H]^+^ 388.0762; found: 388.0764.

#### 7-OMe-2- (3′-P-chlorophenyl-4′(5H)-thiazolidinone)-quinoxaline-1, 4-di-*N*-oxide (2p)

Yellow crystals; yield: 47%; mp: 253–256°C; ^1^H NMR (400 MHz) δ 8.62 (s, 1H), 8.39 (d, *J* = 9.5 Hz, 1H), 7.68 (d, *J* = 2.7 Hz, 1H), 7.61–7.51 (m, 3H), 7.46–7.39 (m, 2H), 6.82 (s, 1H), 4.12 (dd, *J* = 15.3, 1.4 Hz, 1H), 3.96 (s, 3H), 3.83 (d, *J* = 15.3 Hz, 1H); ^13^C NMR (100 MHz) δ 171.68, 162.55, 139.47, 139.21, 136.41, 133.51, 131.56, 130.14, 129.64, 126.89, 124.52, 121.68, 99.35, 57.55, 56.99, 33.29; MS (ESI) *m*/*z* 404 [M+H]^+^. HR-MS (ESI): calculated for C_18_H_14_ClN_3_O_4_S [M+H]^+^ 404.0466; found: 404.0427.

#### 7-F-2- (3′-phenyl-4′(5H)-thiazolidinone)-quinoxaline-1, 4-di-*N*-oxide (2q)

Yellow crystals; yield: 44%; mp: 249–252°C; ^1^H NMR (400 MHz) δ 8.66 (s, 1H), 8.55 (dd, *J* = 9.5, 5.1 Hz, 1H), 8.12 (dd, *J* = 8.9, 2.7 Hz, 1H), 7.91 (ddd, *J* = 9.6, 7.9, 2.8 Hz, 1H), 7.52 (d, *J* = 7.9 Hz, 2H), 7.38 (dd, *J* = 10.7, 5.1 Hz, 2H), 7.26–7.18 (m, 1H), 6.81 (s, 1H), 4.12–4.09 (m, 1H), 3.83 (d, *J* = 15.3 Hz, 1H); ^13^C NMR (100 MHz) δ 171.62, 165.15, 162.63, 141.46, 139.05, 138.94, 137.50, 135.65, 130.54, 129.70, 127.48, 125.18, 123.49, 123.39, 122.71, 122.45, 105.97, 105.69, 57.73, 33.28; MS (ESI) *m*/*z* 358 [M+H]^+^. HR-MS (ESI): calculated for C_17_H_12_FN_3_O_3_S [M+Na]^+^ 380.0476; found: 380.0487.

#### 7-F-2- (3′-P-methylphenyl-4′(5H)-thiazolidinone)-quinoxaline-1, 4-di-*N*-oxide (2r)

Yellow crystals; yield: 41%; mp: 249–251°C; ^1^H NMR (400 MHz) δ 8.65 (s, 1H), 8.55 (dd, *J* = 9.5, 5.1 Hz, 1H), 8.13 (dd, *J* = 8.9, 2.7 Hz, 1H), 7.91 (ddd, *J* = 9.6, 7.9, 2.8 Hz, 1H), 7.38 (d, *J* = 7.8 Hz, 2H), 7.17 (d, *J* = 8.2 Hz, 2H), 6.77 (s, 1H), 4.10 (dd, *J* = 15.2, 1.5 Hz, 1H), 3.81 (d, *J* = 15.2 Hz, 1H), 2.23 (s, 3H); ^13^C NMR (100 MHz) δ 171.51, 165.15, 162.63, 141.48, 139.03, 138.92, 136.98, 135.66, 134.90, 130.60, 130.17, 125.12, 123.49, 123.39, 122.72, 122.46, 105.97, 105.69, 57.81, 33.32, 20.97; MS (ESI) *m*/*z* 372 [M+H]^+^. HR-MS (ESI): calculated for C_18_H_14_FN_3_O_3_S [M+H]^+^ 372.0813; found: 372.0789.

#### 7-F-2- (3′-P-methoxyphenyl-4′(5H)-thiazolidinone)-quinoxaline-1, 4-di-*N*-oxide (2s)

Yellow crystals; yield: 49%; mp: 251–253°C; ^1^H NMR (400 MHz) δ 8.69 (s, 1H), 8.55 (dd, *J* = 9.5, 5.1 Hz, 1H), 8.13 (dd, *J* = 8.9, 2.7 Hz, 1H), 7.91 (ddd, *J* = 9.6, 7.9, 2.8 Hz, 1H), 7.45–7.34 (m, 2H), 6.95–6.86 (m, 2H), 6.73 (s, 1H), 4.09 (dd, *J* = 15.2, 1.6 Hz, 1H), 3.80 (d, *J* = 15.2 Hz, 1H), 3.70 (s, 3H); ^13^C NMR (100 MHz) δ 171.52, 165.15, 162.63, 158.39, 141.55, 139.05, 138.93, 135.67, 130.73, 130.05, 127.06, 123.50, 123.40, 122.71, 122.45, 114.85, 105.98, 105.70, 58.04, 55.69, 33.28; MS (ESI) *m*/*z* 388 [M+H]^+^. HR-MS (ESI): calculated for C_18_H_14_FN_3_O_4_S [M+H]^+^ 388.0762; found: 388.0789.

#### 7-F-2- (3′-P-fluorophenyl-4′(5H)-thiazolidinone)-quinoxaline-1, 4-di-*N*-oxide (2t)

Yellow crystals; yield: 53%; mp: 250–253°C; ^1^H NMR (400 MHz) δ 8.71 (s, 1H), 8.55 (dd, *J* = 9.5, 5.1 Hz, 1H), 8.13 (dd, *J* = 8.9, 2.7 Hz, 1H), 7.91 (ddd, *J* = 9.7, 8.0, 2.7 Hz, 1H), 7.56 (dd, *J* = 8.1, 4.8 Hz, 2H), 7.26–7.16 (m, 2H), 6.79 (s, 1H), 4.10 (dd, *J* = 15.3, 1.5 Hz, 1H), 3.83 (d, *J* = 15.3 Hz, 1H); ^13^C NMR (100 MHz) δ 171.69, 165.15, 162.63, 162.05, 159.62, 141.34, 139.12, 139.01, 135.69, 133.72, 133.69, 130.65, 127.80, 127.71, 123.50, 123.40, 122.69, 122.43, 116.63, 116.40, 105.98, 105.70, 57.80, 33.21; MS (ESI) *m*/*z* 376 [M+H]^+^. HR-MS (ESI): calculated for C_17_H_11_F_2_N_3_O_3_S [M+H]^+^ 376.0562; found: 376.0570.

#### 7-F-2- (3′-P-chlorophenyl-4′(5H)-thiazolidinone)-quinoxaline-1, 4-di-*N*-oxide (2u)

Yellow crystals; yield: 51%; mp: 249–252°C; ^1^H NMR (400 MHz) δ 8.68 (s, 1H), 8.55 (dd, *J* = 9.5, 5.1 Hz, 1H), 8.13 (dd, *J* = 8.9, 2.7 Hz, 1H), 7.91 (ddd, *J* = 9.6, 7.9, 2.8 Hz, 1H), 7.57 (d, *J* = 8.8 Hz, 2H), 7.47–7.40 (m, 2H), 6.83 (s, 1H), 4.16–4.05 (m, 1H), 3.83 (d, *J* = 15.3 Hz, 1H); ^13^C NMR (100 MHz) δ 171.66, 165.15, 162.63, 141.34, 139.14, 139.03, 136.35, 135.68, 131.57, 130.53, 129.63, 126.88, 123.49, 123.39, 122.68, 122.42, 105.98, 105.70, 57.47, 33.19; MS (ESI) *m*/*z* 392 [M+H]^+^. HR-MS (ESI): calculated for C_17_H_11_ClFN_3_O_3_S [M+H]^+^ 392.0266; found: 392.0225.

#### 7-Cl-2- (3′-phenyl-4′(5H)-thiazolidinone)-quinoxaline-1, 4-di-*N*-oxide (2v)

Yellow crystals; yield: 52%; mp: 248–250°C; ^1^H NMR (400 MHz) δ 8.65 (s, 1H), 8.47 (d, *J* = 9.2 Hz, 1H), 8.35 (d, *J* = 2.2 Hz, 1H), 8.00 (dd, *J* = 9.2, 2.3 Hz, 1H), 7.52 (d, *J* = 7.8 Hz, 2H), 7.40–7.34 (m, 2H), 7.26–7.20 (m, 1H), 6.81 (s, 1H), 4.12 (dd, *J* = 15.3, 1.5 Hz, 1H), 3.83 (d, *J* = 15.3 Hz, 1H); ^13^C NMR (100 MHz) δ 171.62, 142.17, 138.34, 137.66, 137.49, 137.24, 133.43, 130.52, 129.71, 127.49, 125.20, 122.26, 119.16, 57.74, 33.24; MS (ESI) *m*/*z* 374 [M+H]^+^. HR-MS (ESI): calculated for C_17_H_12_ClN_3_O_3_S [M+H]^+^ 374.0361; found: 374.0337.

#### 7-Cl-2- (3′-P-methylphenyl-4′(5H)-thiazolidinone)-quinoxaline-1, 4-di-*N*-oxide (2w)

Yellow crystals; yield: 58%; mp: 250–252°C; ^1^H NMR (400 MHz) δ 8.62 (d, *J* = 11.8 Hz, 1H), 8.51–8.42 (m, 1H), 8.40–8.30 (m, 1H), 7.99 (ddd, *J* = 13.4, 9.2, 2.3 Hz, 1H), 7.38 (d, *J* = 7.7 Hz, 2H), 7.17 (d, *J* = 8.4 Hz, 2H), 6.76 (s, 1H), 4.10 (dd, *J* = 15.2, 1.4 Hz, 1H), 3.81 (d, *J* = 15.2 Hz, 1H), 2.23 (s, 3H); ^13^C NMR (100 MHz) δ 171.52, 142.14, 138.32, 137.67, 137.24, 136.98, 134.89, 133.43, 130.62, 130.17, 125.13, 122.26, 119.52, 57.78, 33.20, 20.97; MS (ESI) *m*/*z* 388 [M+H]^+^. HR-MS (ESI): calculated for C_18_H_14_ClN_3_O_3_S [M+H]^+^ 388.0517; found: 388.0483.

#### 7-Cl-2- (3′-P-methoxyphenyl-4′(5H)-thiazolidinone)-quinoxaline-1, 4-di-*N*-oxide (2x)

Yellow crystals; yield: 56%; mp: 252–254°C; ^1^H NMR (400 MHz) δ 8.68 (s, 1H), 8.47 (d, *J* = 9.2 Hz, 1H), 8.36 (d, *J* = 2.2 Hz, 1H), 8.00 (dd, *J* = 9.2, 2.3 Hz, 1H), 7.45–7.34 (m, 2H), 6.94–6.87 (m, 2H), 6.72 (s, 1H), 4.08 (dd, *J* = 15.1, 1.6 Hz, 1H), 3.80 (d, *J* = 15.2 Hz, 1H), 3.70 (s, 3H); ^13^C NMR (100 MHz) δ 171.53, 158.40, 142.26, 138.33, 137.66, 137.25, 133.42, 130.76, 130.04, 127.08, 122.27, 119.53, 114.85, 58.00, 55.70, 33.16; MS (ESI) *m*/*z* 404 [M+H]^+^. HR-MS (ESI): calculated for C_18_H_14_ClN_3_O_4_S [M+H]^+^ 404.0466; found: 404.0451.

#### 7-Cl-2- (3′-P-fluorophenyl-4′(5H)-thiazolidinone)-quinoxaline-1, 4-di-*N*-oxide (2y)

Yellow crystals; yield: 55%; mp: 251–253°C; ^1^H NMR (400 MHz) δ 8.68 (d, *J* = 11.9 Hz, 1H), 8.47 (dd, *J* = 5.7, 3.5 Hz, 1H), 8.38 (d, *J* = 9.1 Hz, 1H), 7.99 (ddd, *J* = 12.8, 9.2, 2.3 Hz, 1H), 7.62–7.50 (m, 2H), 7.27–7.17 (m, 2H), 6.79 (s, 1H), 4.09 (dd, *J* = 15.2, 1.1 Hz, 1H), 3.83 (d, *J* = 15.3 Hz, 1H); ^13^C NMR (100 MHz) δ 171.70, 162.07, 159.63, 142.70, 138.40, 137.65, 137.27, 133.70, 133.67, 133.40, 130.05, 127.83, 127.74, 122.27, 119.52, 116.63, 116.40, 57.84, 33.17; MS (ESI) *m*/*z* 392 [M+H]^+^. HR-MS (ESI): calculated for C_17_H_11_ClFN_3_O_3_S [M+H]^+^ 392.0266; found: 392.0237.

#### 7-Cl-2- (3′-P-chlorophenyl-4′(5H)-thiazolidinone)-quinoxaline-1, 4-di-*N*-oxide (2z)

Yellow crystals; yield: 58%; mp: 252–255°C; ^1^H NMR (400 MHz) δ 8.66 (d, *J* = 12.0 Hz, 1H), 8.47 (dd, *J* = 5.7, 3.5 Hz, 1H), 8.40–8.34 (m, 1H), 7.99 (ddd, *J* = 12.7, 9.2, 2.3 Hz, 1H), 7.57 (d, *J* = 8.7 Hz, 2H), 7.47–7.40 (m, 2H), 6.83 (s, 1H), 4.10 (d, *J* = 14.2 Hz, 1H), 3.83 (d, *J* = 15.4 Hz, 1H); ^13^C NMR (100 MHz) δ 171.66, 141.99, 138.43, 137.65, 137.26, 136.34, 133.40, 131.58, 130.53, 129.64, 126.91, 122.26, 119.53, 57.48, 33.19; MS (ESI) *m*/*z* 407 [M+H]^+^. HR-MS (ESI): calculated for C_17_H_11_Cl_2_N_3_O_3_S [M+H]^+^ 407.9971; found: 407.9982.

### Antimycobacterial Activity

All synthesized compounds were tested *in vitro* for their antimycobacterial activity at an Animal Biological Safety Level-3 (ABSL-3) facility in a state key laboratory of agricultural microbiology within HuaZhong Agricultural University. Antitubercular activity was evaluated against *Mtb* H37Rv (ATCC27294) using a microplate Alamar blue assay (MABA) (Jimenez-Arellanes et al., 2003). Briefly, the strain was cultured at 37°C in Middlebrook 7H9 broth supplemented with 0.2% glycerol and 10% oleic acid albumin dextrose catalase (OADC; Difco) and grew to the logarithmic phase. The culture was adjusted to 1 × McFarland′s nephelometer and then diluted 20-fold with fresh broth medium. In sterile flat-bottom 96-well microplates, 100 μg/mL of each compound was decreasingly diluted (by two-fold). The final concentration of each compound was in the range of 0.10–50 μg/mL. Next, 100 μL of the prepared bacterial suspension was added to each well and mixed with the diluted compounds. One well with broth only and one well with bacteria only were set as the negative and growth control, respectively. The microplates were incubated at 37°C in a 5% CO_2_ atmosphere for 5 days. Twenty μL Alamar blue dye and 12 μL of sterile 10% Tween 80 were added to one growth control well, and the plates were incubated again at 37°C for 24 h. After this incubation, if the well-became pink, the same reagent was added to all of the other wells, and the plate was incubated for an additional 24 h. The MIC was defined as the lowest concentration of sample in which the color shift from blue to pink was not observed. Rifampin was used as a reference standard (MIC = 0.125 μg/mL).

### Cytotoxicity Assay

The compounds that exhibited antimycobacterial activity against *Mtb* strain H37Rv (MIC < 6.25 μg/mL) were tested for cytotoxic activity against the VERO cell line using an MTT assay. VERO cells (2 × 10^4^ cells) were seeded into 96-well plates and incubated for 12 h. Serial dilutions of the compounds were mixed with the cell suspension in the plates. Both treated cells and blank and solvent controls were incubated for 72 h, followed by incubation with MTT (0.5 mg/mL) for another 4 h. The microplates were measured at 570 nm using a multimode reader (Infinite M200, Tecan). The percentage of viable cells was calculated as the mean with respect to the controls set to 100%. Triplicate wells were analyzed for each concentration. IC_50_ was defined as the concentration that reduced the viable cells by 50% compared to the untreated control cells. It was considered significant when the SI was more than 10.

### Macrophage Assay

Compounds with an MIC ≤ 6.25 μg/mL and an SI > 10 were then tested to evaluate efficacy *in vitro* in a TB-infected macrophage model according to Skinner et al. (1994). EC_90_ and EC_99_ are defined as the concentrations effecting 90 and 99% reductions in residual mycobacterial growth after 7 days as compared to untreated controls. Compounds with EC_90_ > 16 × MIC are considered inactive.

### Antifungal Activity

All synthesized compounds and the positive control drugs amphotericin B and ketoconazole were tested for their *in vitro* antifungal activity. Antifungal activity was evaluated against four fungi, *C. albicans* (ATCC90028), *C. tropicalis* (ATCC7349), *A. fumigatus* (3.5352), and *C. neoformans* (2.3201), by broth microdilution according to the protocols from the NCCLS (Jiang et al., 2014). Briefly, all tested compounds were dissolved in DMSO, and the solutions were diluted with fresh broth medium. All of the fungi-seeded broth (1 × 10^5^ colony-forming units [CFU]/mL) were prepared in RPMI 1640 medium and added into the serially diluted drug solution. The tubes were incubated at 28°C, and the MIC (μg/mL) was recorded 72–96 h post-incubation. Broth control (without fungi), growth controls (with fungi and without drug), solvent (DMSO) control, and drug control for both test drugs and standard drugs were set under identical conditions. The minimum drug concentration in the tubes in which no apparent growth of the organism was observed represented the MIC of the compounds.

### 3D-QSAR

3D-QSAR models were established according previously reported procedures (Radadiya et al., 2014; Pan et al., 2016). All of the molecular modeling calculations were performed using SYBYL-X 2.0 installed on a Dell GPCJ4X1 workstation running Windows 7. Energy minimizations were performed using the MMFF94 force field with the Powell conjugate gradient minimization algorithm and a convergence criterion of 0.005 kcal/mol^*^Å. Charges were calculated by the Gasteiger-Hücker method in the software. Alignment of molecules was performed using the database alignment method in SYBYL.

## Results and Discussion

### Chemistry

Compound design began with the molecular hybridization knowledge that two specific structural moieties, thiazolidinones and the quinoxaline ring, could be fused into one molecule with the goal of obtaining novel compounds with better biological activity (Figure 1). Twenty-six novel quinoxaline-1,4-di-*N*-oxides containing a thiazolidinone moiety were prepared according to the synthetic routes illustrated in Scheme 1. The 5-substituted-benzofurans (**II**) were obtained by the reaction with 4-substituted-2-nitroaniline (**I**), according to the methods previously described and without further purification before proceeding to the next step (Ortega et al., 2002; Zhao et al., 2016). Compounds **III** were synthesized by the reaction of 5-substituted-benzofuroxan (**II**) with pyruvic aldehyde dimethyl acetal, using pyrrolidine as the catalyst (Haddadin and Issidorides, 1993; Zarranz et al., 2005). Compounds **III** were hydrolyzed with 6N HCl to form compound **IV**; compound **IV** was precipitated by adjusting the pH value of the reaction mixture to 7.5. Knoevenagel condensation of 2-iminothiazolidin-4-one (**3**) with 2-quinoxalinecarboxaldehyde-1, 4-di-*N*-oxide (**IV**) in the presence of ammonium acetate and glacial acetic acid provided compound **V** (Jadav et al., 2015; Ghareb et al., 2017). Compounds **IV** were refluxed with thioglycollic acid and ammonium carbonate for 18 h to get compounds **VI** (El Nezhawy et al., 2009). Subsequently, we modified the structure of thiazolidinone to obtain new derivatives. Aldimine condensation reaction of compounds **IV** with various aromatic amine/cyclopropylamine gave compounds **VII** under alkaline conditions. Finally, compounds **VIII** were refluxed with thioglycollic acid for 8 h to get the final products, **2a−2z**.

All quinoxaline-1,4-di-*N*-oxide derivatives were purified by flash column chromatography in silica gel to get the pure compounds. The structures were characterized by ^1^H NMR, ^13^C NMR, and high-resolution mass spectrometry (HR-MS) analysis.

### Biological Evaluation

#### *In vitro* Antimycobacterial Activity

The novel quinoxaline-1,4-di-*N*-oxide derivatives were investigated for their antimycobacterial activity against *Mtb* strain H37Rv using MABA with rifampin as the positive control (Jimenez-Arellanes et al., 2003). First, compounds **V** and **VI** were synthesized; they have different thiazolidinones at the C2 side chain of the quinoxaline ring. When evaluating their antimycobacterial activities, compound **VI** (MIC = 6.25 μg/mL) exhibited better antimycobacterial activity against *Mtb* strain H37Rv compared to compound **V** (MIC > 50 μg/mL). These data indicate that 1,3-thiazolidin-4-one directly linked to the C2 side chain of the quinoxaline ring displays good antimycobacterial activity. The results showed that structural modification should be based on compounds **VI**. Hence, we changed the synthesis strategy and obtained a series of new derivatives (**2a−2z**) with different substituents at R1 and R2 of compounds **VIII**. The theoretical lipophilicities (*C*log*P*) of the derivatives were calculated using SYBYL-X 2.0 and compared with their antimycobacterial activity (Table 1). The results for the *in vitro* antimycobacterial activity of novel quinoxaline-1,4-di-*N*-oxide derivatives against H37Rv are also listed in Table 1. Twelve out of the 26 evaluated compounds passed the cut-off established by the TAACF (MIC ≤ 6.25 μg/mL). Compounds **2t**, **2u**, **2y**, and **2z** showed the best antimycobacterial activity (MIC = 1.56 μg/mL). This was slightly lower than that of the positive control (rifampicin, MIC = 0.125 μg/mL) but higher than that of the quinoxalines. It seemed that chlorine atoms of the derivatives are responsible for their higher lipophilicity (*C*log*P* > 0.7) and strongly enhance their antimycobacterial activities. When a chlorine atom was attached to position R1 or R2, such as in compounds **2e**, **2k**, **2u**, **2v**, **2y**, and **2z** (*C*log*P* = 0.71, 1.21, 1.00, 0.86, 1.00, and 1.57, respectively), the antimycobacterial activity was significantly improved (MIC ≤ 3.13 μg/mL). It was worth noting that compounds **2d**, **2j**, and **2t** also exhibited good antimycobacterial activity, whereas they have lower lipophilicity (*C*log*P* < 0.7). This result indicated that introducing a halogen atom at position R1 or R2 could increase the antimycobacterial activity. The above results showed that the structural modification of the quinoxaline-1,4-di-*N*-oxide was successful.

**Table 1:** Results of antimycobacterial and cytotoxicity assays.

**Compounds**	**R1**	**R2**	***C*log*P*^**a**^**	**MIC^**b**^ (μg/mL)**	**${\text{IC}}_{50}^{\text{c}}$ (μg/mL)**	**SI^**d**^ (IC_**50**_/MIC)**
**V**	H	/	/	>50	/	/
**VI**	H	H	/	6.25	/	/
**2a**	H	H	−0.01	6.25	143.6	23
**2b**	H	-CH_3_	0.49	12.5	/	/
**2c**	H	-OMe	−0.09	12.5	/	/
**2d**	H	-F	0.14	3.13	122.8	39.2
**2e**	H	-Cl	0.71	3.13	115.7	37
**2f**	H	/	−1.54	25	/	/
**2g**	CH_3_	H	0.49	12.5	/	/
**2h**	CH_3_	-CH_3_	0.99	25	/	/
**2i**	CH_3_	-OMe	0.41	50	/	/
**2j**	CH_3_	-F	0.64	6.25	113.5	18.2
**2k**	CH_3_	-Cl	1.21	3.13	102.2	32.7
**2l**	OCH_3_	H	0.26	100	/	/
**2m**	OCH_3_	-CH_3_	0.76	100	/	/
**2n**	OCH_3_	-OMe	0.18	200	/	/
**2o**	OCH_3_	-F	0.40	50	/	/
**2p**	OCH_3_	-Cl	0.97	50	/	/
**2q**	F	H	0.29	6.25	126.4	20.2
**2r**	F	-CH_3_	0.79	12.5	/	/
**2s**	F	-OMe	0.21	25	/	/
**2t**	F	-F	0.43	1.56	100.9	64.7
**2u**	F	-Cl	1.00	1.56	91.2	58.5
**2v**	Cl	H	0.86	3.13	118.8	38
**2w**	Cl	-CH_3_	1.36	6.25	125.3	20
**2x**	Cl	-OMe	0.78	12.5	/	/
**2y**	Cl	-F	1.00	1.56	106.8	68.5
**2z**	Cl	-Cl	1.57	1.56	94.6	60.6
**RMP**^***e***^	/	/	/	0.125	/	/

a Calculated by SYBYL-X 2.0 (Tripos, Inc.).

b Actual minimum inhibitory concentration (MABA assay).

c Measurement of cytotoxicity in VERO cells.

d Selectivity index.

e *Rifampicin*.

In general, most compounds with a phenyl group or substituted phenyl group in position R2, like compounds **2a**, **2d**, **2e**, **2k**, **2t**, **2u**, **2y**, and **2z**, exhibited good antimycobacterial activity. On the contrary, compounds **2l−2p** showed low activity against *Mtb* strain H37Rv. Considering that the antimycobacterial activity results from the compounds′ structures, which have a phenyl group substituted at position R2, we decided to keep the phenyl group in position R2 and modify the substitution of the phenyl group. Different substituents were placed at the *para* position of the phenyl group, considering the fluoro and chloro moieties as electron-withdrawing groups and methoxy and methyl groups as electron-releasing groups. Further analysis of the antimycobacterial activity results (Table 1) shows that an electron-withdrawing moiety on the para position of the phenyl group is an essential requirement to increase activity. Similarly, introducing an electron-releasing group at position R1 of the quinoxaline ring increases the antimycobacterial activity, and the activity improved in this order: fluoro = chloro > H > methyl.

Further analysis of the activity profile of the prepared collection revealed some SARs. The presence of an electron-withdrawing moiety at position R1 or R2 can significantly improve the antimycobacterial activity. However, an electron-releasing group in position R1 or R2 reduces the activity of the compounds. Replacing the halogen atom with a methyl or methoxy is detrimental to the activity. In position R2, we conclude that the substitution of an electron-withdrawing moiety at the *para* position of the phenyl group increases the antimycobacterial activity; the same is true for position R1.

#### Cytotoxicity and SI

Preliminary *in vitro* screening of the cytotoxicity of the 12 active compounds was assayed on VERO cells. The cytotoxicity was evaluated as the IC_50_ value and SI (SI = IC_50_/MIC); these values are listed in Table 1. The SI value (SI > 10) indicates the concentration of the compounds at which they are active against mycobacteria but are non-toxic toward host cells (Hartkoorn et al., 2007). All 12 compounds exhibited an SI > 10; these data indicate their potential for antimycobacterial activity. Notably, compounds **2t**, **2u**, **2y**, and **2z** showed the best activity, with SI values ranging from 58.5 to 68.5.

#### Macrophage Assay

We selected four compounds (**2t**, **2u**, **2y**, and **2z**), all of which have high SI values, to test their *in vitro* efficacy in a TB-infected macrophage model (Table 2). Each compound showed excellent activity in the macrophage assay (EC_90_/MIC = 1.06, 0.54, 0.85, and 0.59, respectively). Based on the results, we conclude that compounds **2t**, **2u**, **2y**, and **2z** are active against mycobacterial and are non-toxic. This activity, selectivity, and low cytotoxicity render these four compounds valid leads for additional studies as well as for synthesizing new compounds that have good activity against *Mtb* strain H37Rv.

**Table 2:** Results of macrophage assay.

**Compounds**	**EC90 (μg/mL)^**a**^**	**EC99 (μg/mL)^**a**^**	**EC90/MIC^**b**^**
**2t**	1.66	5.25	1.06
**2u**	0.84	3.27	0.54
**2y**	1.33	4.58	0.85
**2z**	0.92	3.93	0.59

a EC_90_ and EC_99_ are defined as the concentrations causing 90 and 99% reductions in residual mycobacterial growth after 7 days as compared to untreated controls.

b Compounds with EC_90_ > 16*/MIC are considered inactive.

#### *In vitro* Antifungal Activity

Table 3 presents the results for the *in vitro* antifungal activity of compounds **2a–2z**, amphotericin B, and ketoconazole against *C. albicans* (ATCC90028), *C. tropicalis* (ATCC7349), *A. fumigatus* (3.5352), and *C. neoformans* (2.3201). In summary, eight out of 26 compounds (**2s−2z**) showed good antifungal activity (MIC ≤ 8 μg/mL) against the aforementioned organisms, while the positive controls (amphotericin B and ketoconazole) showed better activity, with MIC values ranging from 0.25 to 2 μg/mL. Specifically, compounds **2t**, **2u**, **2y**, and **2z** exhibited potential antifungal activities, with an MIC between 2 and 4 μg/mL. The MIC values of the active compounds demonstrated the rationality of our design strategy. On the contrary, compounds **2a−2p** showed lower activity against *C. albicans, C. tropicalis, A. fumigatus*, and *C. neoformans*.

**Table 3:** Antifungal activity of the compounds **2a**−**2z**.

**Compounds**	**MIC (μg/mL)**			
	***Candida albicans***	***C. tropicalis***	***A. fumigatus***	***C. neoformans***
	**ATCC 90028**	**ATCC 7349**	**3.5352**	**2.3201**
**2a**	16	8	32	16
**2b**	16	8	32	32
**2c**	64	32	>64	32
**2d**	16	8	32	8
**2e**	8	8	32	16
**2f**	32	16	64	64
**2g**	32	16	32	32
**2h**	32	8	64	32
**2i**	64	32	>64	16
**2j**	16	8	32	16
**2k**	32	16	32	32
**2l**	64	32	>64	>64
**2m**	>64	64	>64	>64
**2n**	>64	64	>64	>64
**2o**	64	64	64	32
**2p**	64	64	>64	32
**2q**	8	4	16	16
**2r**	8	8	8	16
**2s**	8	8	8	8
**2t**	4	2	4	4
**2u**	4	4	2	4
**2v**	8	8	4	8
**2w**	8	4	8	4
**2x**	8	8	8	8
**2y**	2	4	4	2
**2z**	4	4	4	4
**AMB**^***a***^	1	2	0.5	0.5
**KCZ**^***b***^	0.25	0.5	0.5	0.25

a Amphotericin b.

b *Ketoconazole*.

We observed that substituting a chlorine or fluorine atom at position R1 or R2 or at both positions (**2q−2z**) increased the antifungal activity of the compounds. According to the antifungal activity values (Table 3), the presence of an electron-withdrawing moiety at position R1 or R2, especially a fluorine or chlorine atom, is an essential requirement to increase the antifungal activity. Besides, an electron-releasing group (methyl and methoxy group) at position R1 and R2 obviously reduces or eliminates the antifungal activity.

### 3D-QSAR Analysis

**Figure 2:**
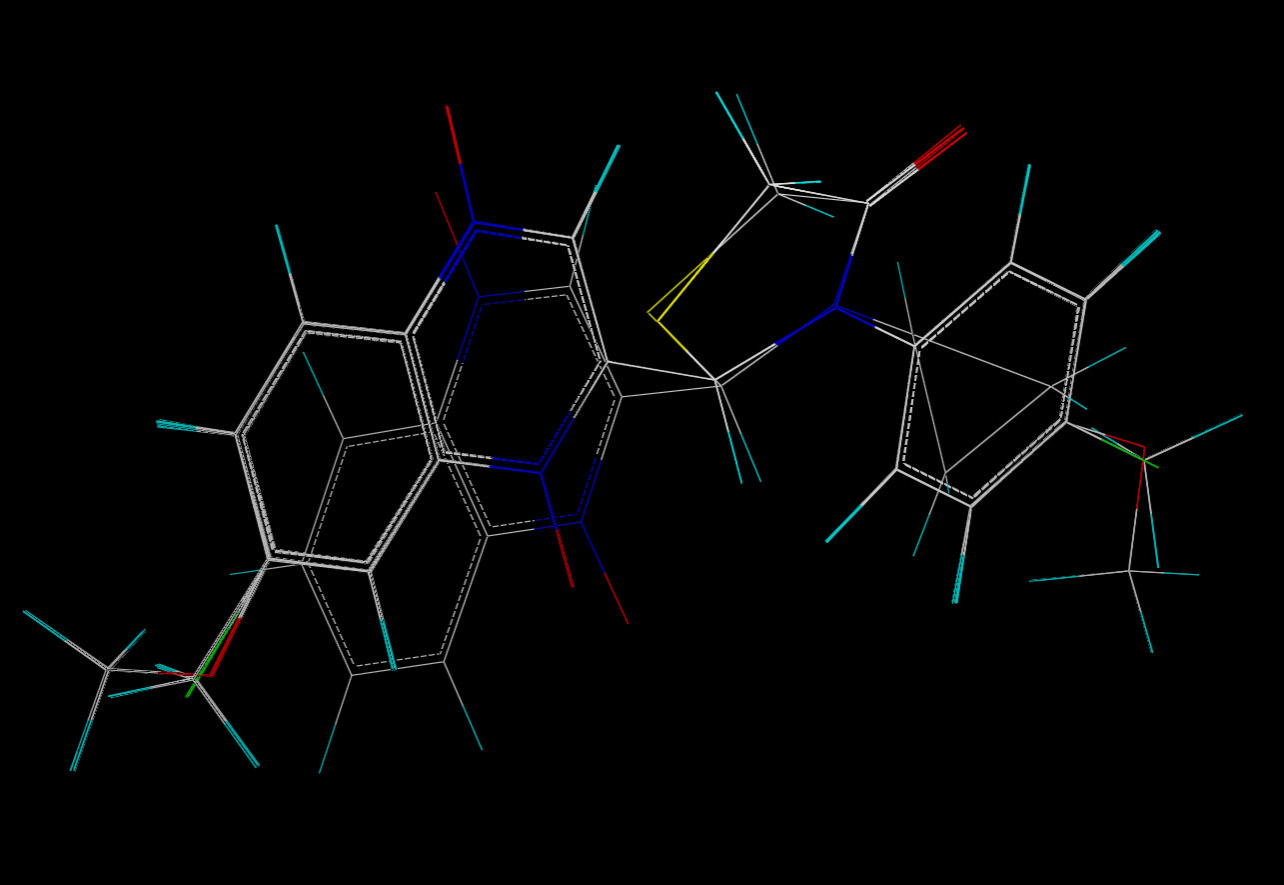
Alignment and superposition of molecules using compound **2y** as the template.

In order to better analyze the SAR, we established CoMFA and CoMSIA models based on the antimycobacterial activity data of the novel quinoxaline-1,4-di-*N*-oxide derivatives. For the alignment, we used the thiazolidinone nucleus as the template, and all the test compounds were aligned to the template molecule (**2y**), results were displayed in Figure 2.

**Figure 3:**
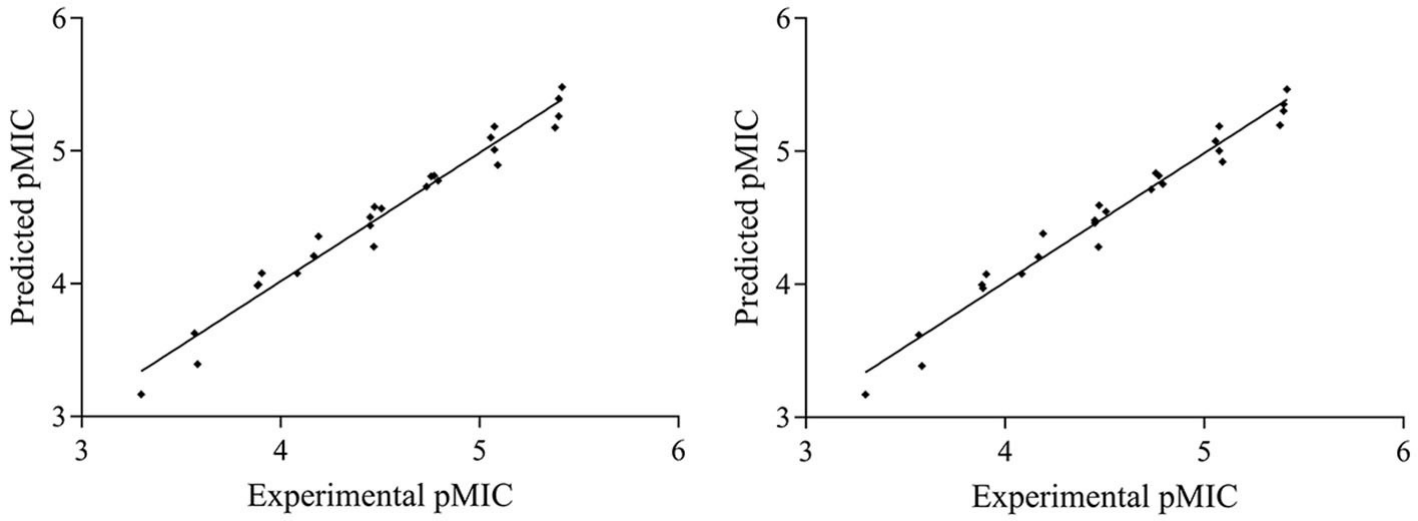
Graphs of predicted vs. experimental *p*MIC of the 26 compounds for CoMFA and CoMSIA 3D-QSAR models.

The Partial Least Squares (PLS) statistic results of the CoMFA and CoMSIA models are summarized in Table 4. The CoMFA model presented a *q*^2^ of 0.914 by leave-one-out cross-validation, which signified good internal predictive ability of the model (*q*^2^ > 0.5). The correlation coefficient (*r*^2^) between the experimental and predicted activities was 0.967, with an *F*-value of 74.947 and a standard error of estimate equal to 0.132. These data suggest that the fitness of the model is 96.7% against experimental results. The contributions to the activity were from the steric (76.2%) and electrostatic (23.8%) fields. The CoMSIA model produced a total of five models by varying the electrostatic, steric, hydrophobic, hydrogen bond donor, and acceptor fields. Among these different field combinations, the CoMSIA model provided the best statistical parameters (Table 4). We performed the CoMSIA study using the same molecule alignment as defined in the CoMFA study. The cross-validated *q*^2^ was 0.918, which reflects satisfactory internal consistency. The correlation coefficient (*r*^2^) value was 0.968, with a standard error of 0.137 and an *F*-value of 54.654. The contributions of steric, electrostatic, hydrophobic, hydrogen bond donor, and acceptor fields in the CoMSIA model were 25.5, 19.1, 54.2, 0, and 1.2%, respectively; these data reflect the relative importance of these fields. The predicted vs. experimental *p*MIC values are listed in Table 5 and are depicted graphically in Figure 3. Notably, these two models are able to predict *p*MIC values in good agreement with the experimental results within a statistically tolerable error range, indicating that they are statistically significant and reliable 3D-QSAR models.

**Table 4:** PLS statistics of CoMFA and CoMSIA 3D-QSAR models.

**Statistical parameters**	**CoMFA**	**CoMSIA**
*q*^2a^	0.914	0.918
ONC^b^	7	9
*r*^2c^	0.967	0.968
SE^d^	0.132	0.137
*F*-values^e^	74.947	54.654
**Field contribution (%)**		
Steric	76.2	25.5
Electrostatic	23.8	19.1
Hydrophobic	/	54.2
H-bond donor	/	/
H-bond acceptor	/	1.2

a Cross-validated correlation coefficient after the leave-one-out procedure.

b Optimum number of components.

c Non-cross-validated correlation coefficient.

d Standard error of estimate.

e *F-test value*.

**Table 5:** Experimental and predicted antituberculosis activities against *M. tuberculosis* strain H37Rv (*p*MIC) of the compounds for CoMFA and CoMSIA models.

**Compounds**	**MIC (μg/mL)**	***p*MIC exp**	**CoMFA**		**CoMSIA**	
			***p*MIC pred**.	**Residual**	***p*MIC pred**.	**Residual**
**2a**	6.25	4.734	4.731	0.003	4.713	0.022
**2b**	12.5	4.451	4.502	−0.051	4.482	−0.031
**2c**	12.5	4.470	4.279	0.191	4.280	0.190
**2d**	3.13	5.057	5.102	−0.045	5.076	−0.019
**2e**	3.13	5.076	5.184	−0.107	5.189	−0.113
**2f**	25	4.084	4.078	0.005	4.078	0.005
**2g**	12.5	4.451	4.439	0.012	4.461	−0.010
**2h**	25	4.167	4.208	−0.041	4.207	−0.041
**2i**	50	3.884	3.986	−0.102	3.996	−0.112
**2j**	6.25	4.773	4.813	−0.040	4.817	−0.044
**2k**	3.13	5.092	4.895	0.197	4.922	0.170
**2l**	100	3.567	3.625	−0.058	3.618	−0.051
**2m**	100	3.583	3.393	0.190	3.386	0.197
**2n**	200	3.300	3.165	0.135	3.171	0.129
**2o**	50	3.889	3.994	−0.106	3.970	−0.081
**2p**	50	3.906	4.079	−0.173	4.076	−0.170
**2q**	6.25	4.757	4.808	−0.051	4.836	−0.079
**2r**	12.5	4.472	4.578	−0.105	4.595	−0.123
**2s**	25	4.190	4.357	−0.167	4.381	−0.191
**2t**	1.56	5.381	5.177	0.204	5.197	0.184
**2u**	1.56	5.399	5.262	0.137	5.305	0.094
**2v**	3.13	5.076	5.008	0.069	5.004	0.072
**2w**	6.25	4.792	4.776	0.016	4.753	0.039
**2x**	12.5	4.508	4.567	−0.058	4.546	−0.037
**2y**	1.56	5.399	5.395	0.004	5.353	0.046
**2z**	1.56	5.415	5.480	−0.064	5.466	−0.051

#### Contour Analysis

We generated contour maps based on the CoMFA and CoMSIA data in Table 5 by using the “STDDEV^*^COEFF” mapping option contoured by contribution. We used compound **2y** (template), the most active molecule of the derivatives, for the presentation of contour maps.

**Figure 4:**
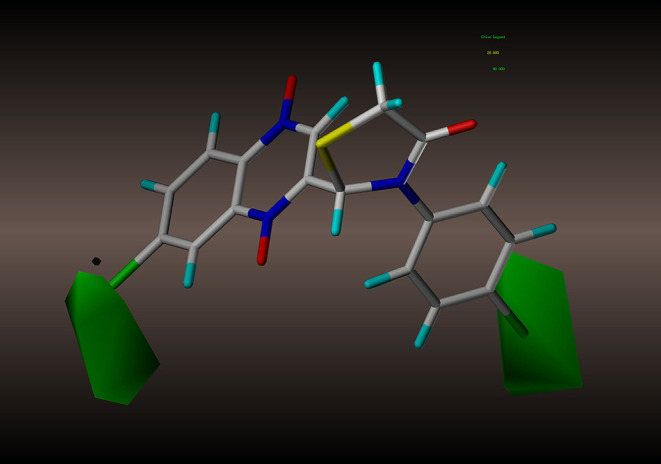
CoMFA steric contour of compound **2y**: green contour favors steric or bulky group, and yellow contour denotes disfavored region.

**Figure 5:**
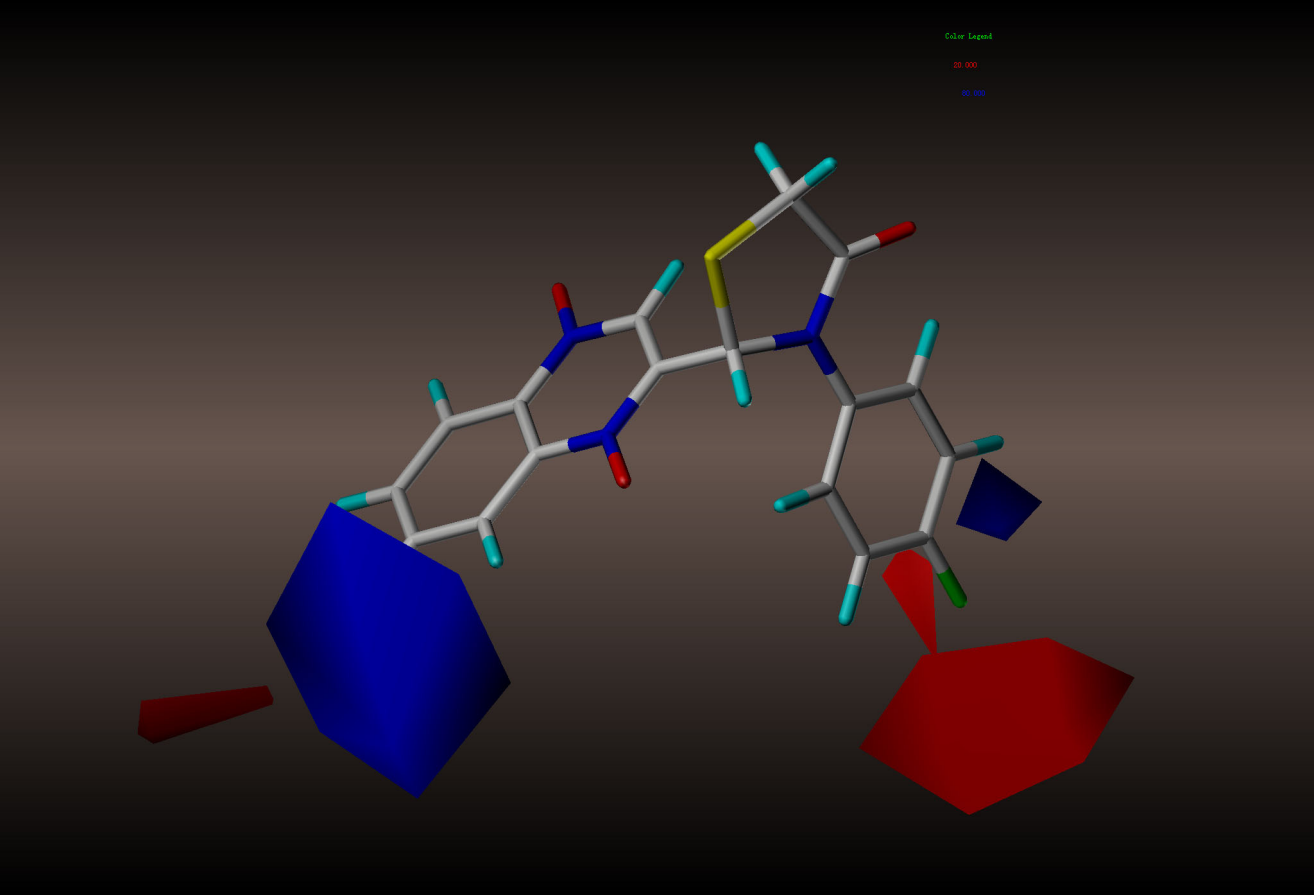
CoMFA electrostatic contour of compound **2y**: blue contour indicates electropositive charge and red contour electronegative charge.

For the CoMFA results, the contour map of the steric field appears in Figure 4. The steric contours are represented in green and yellow; The green contour favors steric or bulky groups, while the yellow contour denotes disfavored regions. We identified two green contours near the C7 position of the quinoxaline ring and the C4 position of the phenyl group. This finding indicates that a sterically bulky substituent is preferred at these positions to increase the activities of compounds. This interpretation is consistent with the experimental finding that compound **2a**, with a sterically bulky substituent, is more active than **2f**. In the contour map of the electrostatic field (Figure 5), the introduction of electropositive substituents in blue regions decreases the affinity, while electronegative groups in red regions may improve the affinity. We noted that a major red contour encloses the C4 position of the phenyl group and that there is a small red contour around the C7 position of the quinoxaline ring. This result is in harmony with the fact that compounds **2a−2z** with electronegative groups at these positions showed better activity. We also observed a large blue contour around the C7 position of the quinoxaline ring and a small blue contour between the C3 and C4 position of the phenyl group. These findings show that electropositive substituents in these positions would decrease the affinity of compounds, a phenomenon that might explain why, when the methyl and methoxyl groups were replaced by fluoro or chloro groups, the compound activities increased dramatically. For example, compounds **2q−2z** were more active than compounds **2l−2p**.

**Figure 6:**
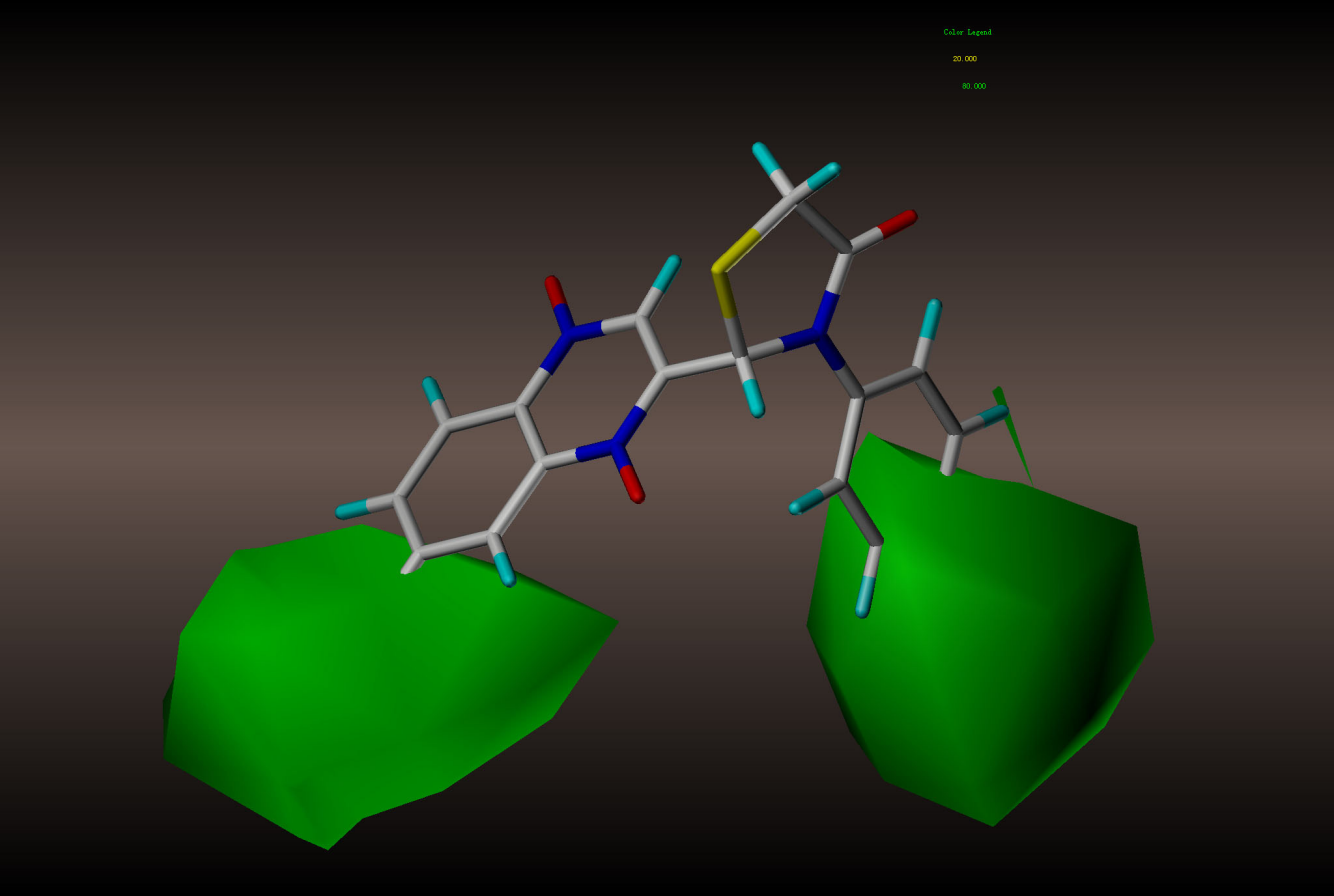
CoMSIA steric contour of compound **2y**: green contour favors steric or bulky group, and yellow contour denotes disfavored region.

**Figure 7:**
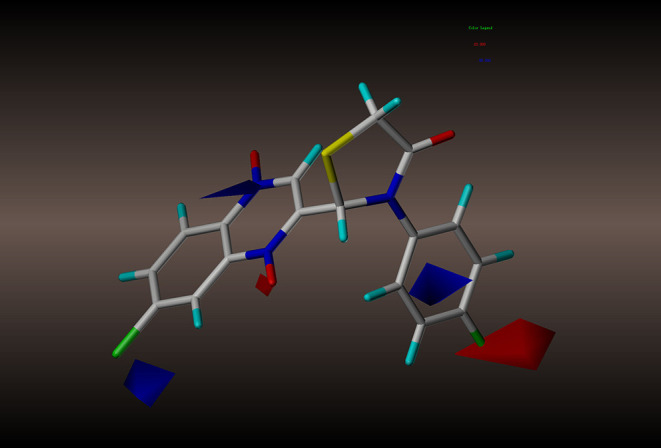
CoMSIA electrostatic contour of compound **2y**: blue contour indicates electropositive charge and red contour electronegative charge.

**Figure 8:**
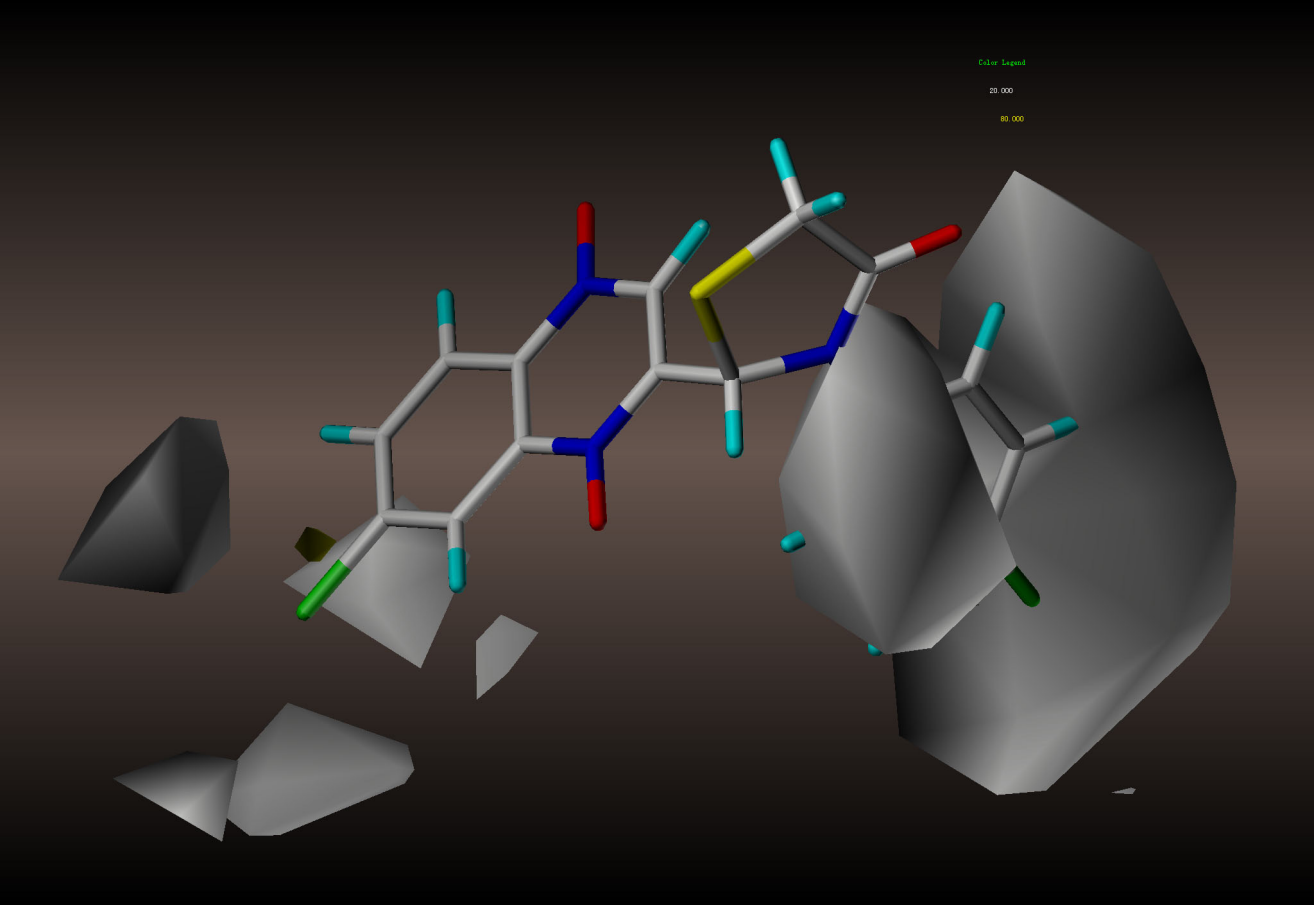
CoMSIA hydrophobic contour maps based on compound **2y**: yellow contours represent regions where hydrophobic groups increase activity, while white contours highlight regions that would favor hydrophilic groups.

**Figure 9:**
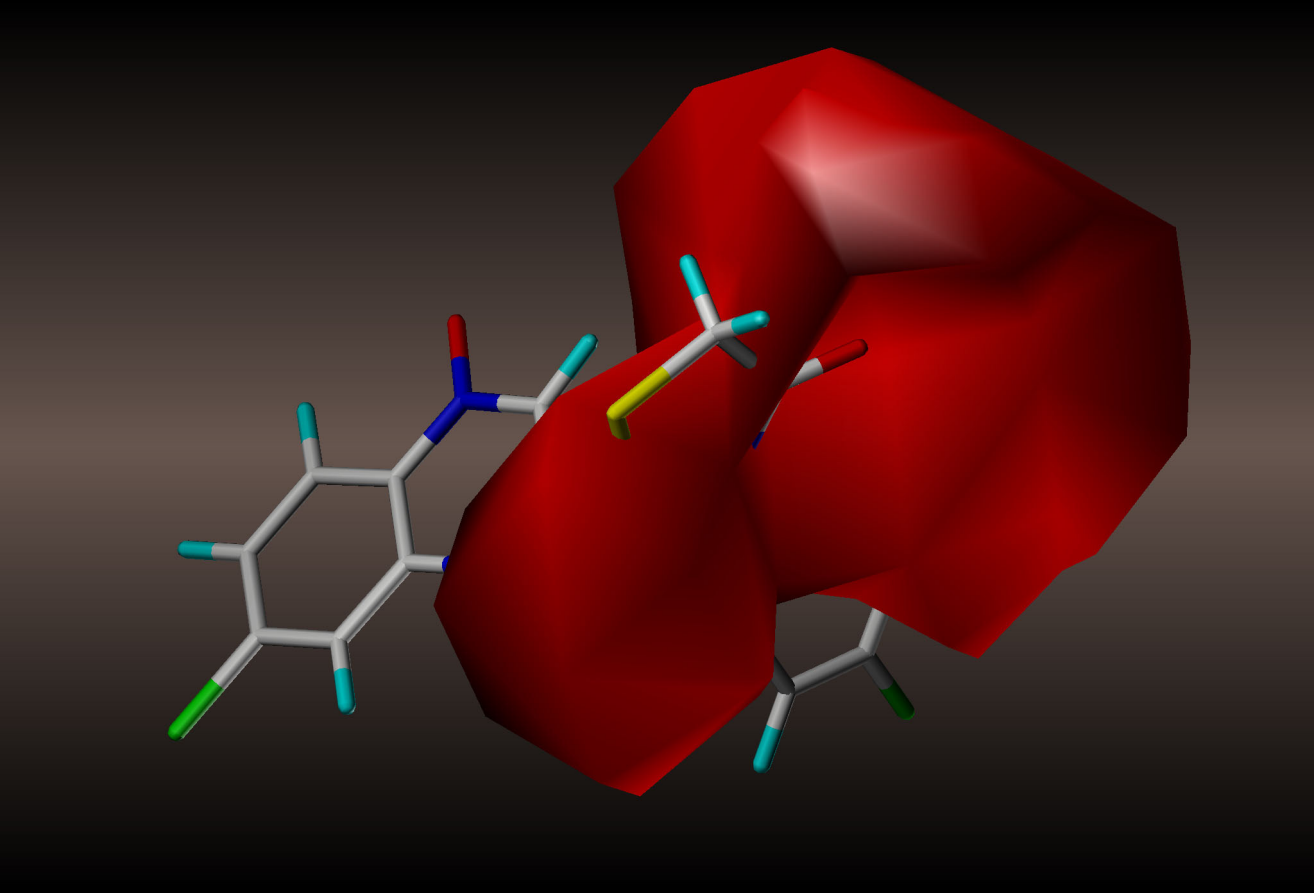
CoMSIA hydrogen bond acceptor contour maps based on compound **2y**: magenta and red contours represent favorable and unfavorable hydrogen bond acceptor regions, respectively.

For the CoMSIA results, we generated five models by varying the electrostatic, steric, hydrophobic, hydrogen bond donor, and acceptor fields. The contour maps of the steric and electrostatic fields are shown in Figures 6, 7, respectively; these results are consistent with the CoMFA results. The hydrophobic contour map is displayed in Figure 8. In this graph, there is one major white contour that covers the phenyl group in the R2 position and many small white contours occur near the C7 position of the quinoxaline ring. These data indicate that the hydrophilic property of this substituent is beneficial to the activities of the compounds. In the hydrogen bond acceptor contour map (Figure 9), one big red contour covers the C2 side chain of the quinoxaline ring. This finding suggests that hydrogen bond acceptor substituents at this position are unfavorable for the activities. Thus, more hydrogen bond donor substituents should be considered at the C2 side chain of the quinoxaline ring to improve the anti-TB activity of compounds. The CoMFA and CoMSIA contour maps can be used as tools to better understand the relationship between structure and activity for different physicochemical properties.

In contrast with our previous study (Pan et al., 2016), we modified the C2 and C7 positions of the quinoxaline ring. Based on the above results, we can draw some conclusions. For the C7 position of the quinoxaline ring and the C4 position of the phenyl group, sterically bulky substituents, or electronegative groups are preferred to increase the activities of compounds. For the C7 position of the quinoxaline ring, a hydrophilic substituent is also beneficial to the activities of compounds. For the C3 and C4 positions of the phenyl group, electropositive substituents would decrease the affinity of compounds. For the C2 side chain of the quinoxaline ring, more hydrogen bond donor substituents should be considered. Specifically, substituting a halogen atom at position R1 could increase the activity dramatically.

## Conclusion

We synthesized 26 novel quinoxaline-1,4-di-*N*-oxides containing a thiazolidinone moiety and evaluated their *in vitro* antimycobacterial and antifungal activities. For antimycobacterial activity, 12 out of the 26 compounds were active, with an MIC ≤ 6.25 μg/mL and SI > 10. Among them, compounds **2t**, **2u**, **2y**, and **2z** displayed the most potent antimycobacterial activity against *Mtb* strain H37Rv (MIC = 1.56 μg/mL). For antifungal activity, eight out of 26 compounds showed good antifungal activity against *C. albicans, C. tropicalis, A. fumigatus*, and *C. neoformans* (MIC ≤ 8 μg/mL). Compounds **2t**, **2u**, **2y**, and **2z** exhibited potential antifungal activities, with an MIC between 2 and 4 μg/ml. The 3D-QSAR results revealed that variations in substitutions at the C7 position and the side chain at the C2 position of the quinoxaline ring could significantly impact the activity. Specifically, compounds with a sterically bulky and electronegative charge substituent at the C7 position of the quinoxaline ring or the terminal of the side chain at the C2 position would exhibit increased antimycobacterial activity. The above results showed that the most suitable substituent on the quinoxaline nucleus is an electron-withdrawing moiety at position R1 or R2, especially that of a halogen atom.

## Data Availability Statement

All datasets generated for this study are included in the article/Supplementary Material.

## Author Contributions

ZY conceived the idea. HZ and YP constructed the workflow. HZ and JZ synthesized and purified the compounds. HZ and WQ performed the experiments. SX and LH analyzed and discussed the data. YP and DC revised the paper. YT and ZL performed and revised the experiments. HZ completed the paper. All authors discussed the results and contributed to the final manuscript.

## Conflict of Interest

The authors declare that the research was conducted in the absence of any commercial or financial relationships that could be construed as a potential conflict of interest.
