# Supplementary material for: Design, Synthesis, and Biological Evaluation of Novel Thiazolidinone-Containing Quinoxaline-1,4-di-N-oxides as Antimycobacterial and Antifungal Agents
Source: Front Chem. 2020 Aug 6;8:598. doi: 10.3389/fchem.2020.00598 (PMC7424068; doi:10.3389/fchem.2020.00598)

**2a**-^1^H NMR


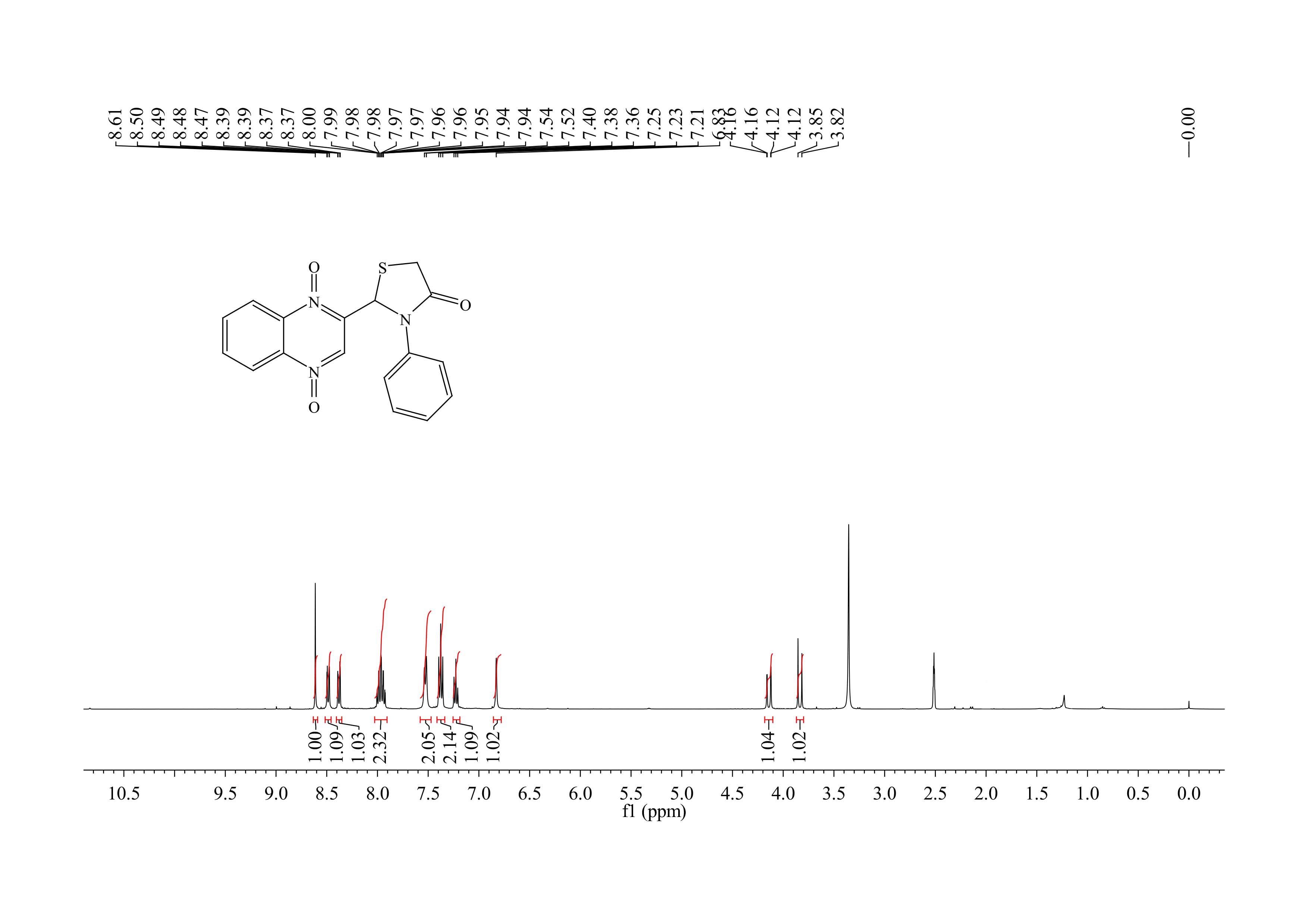


**2a**-^13^C NMR


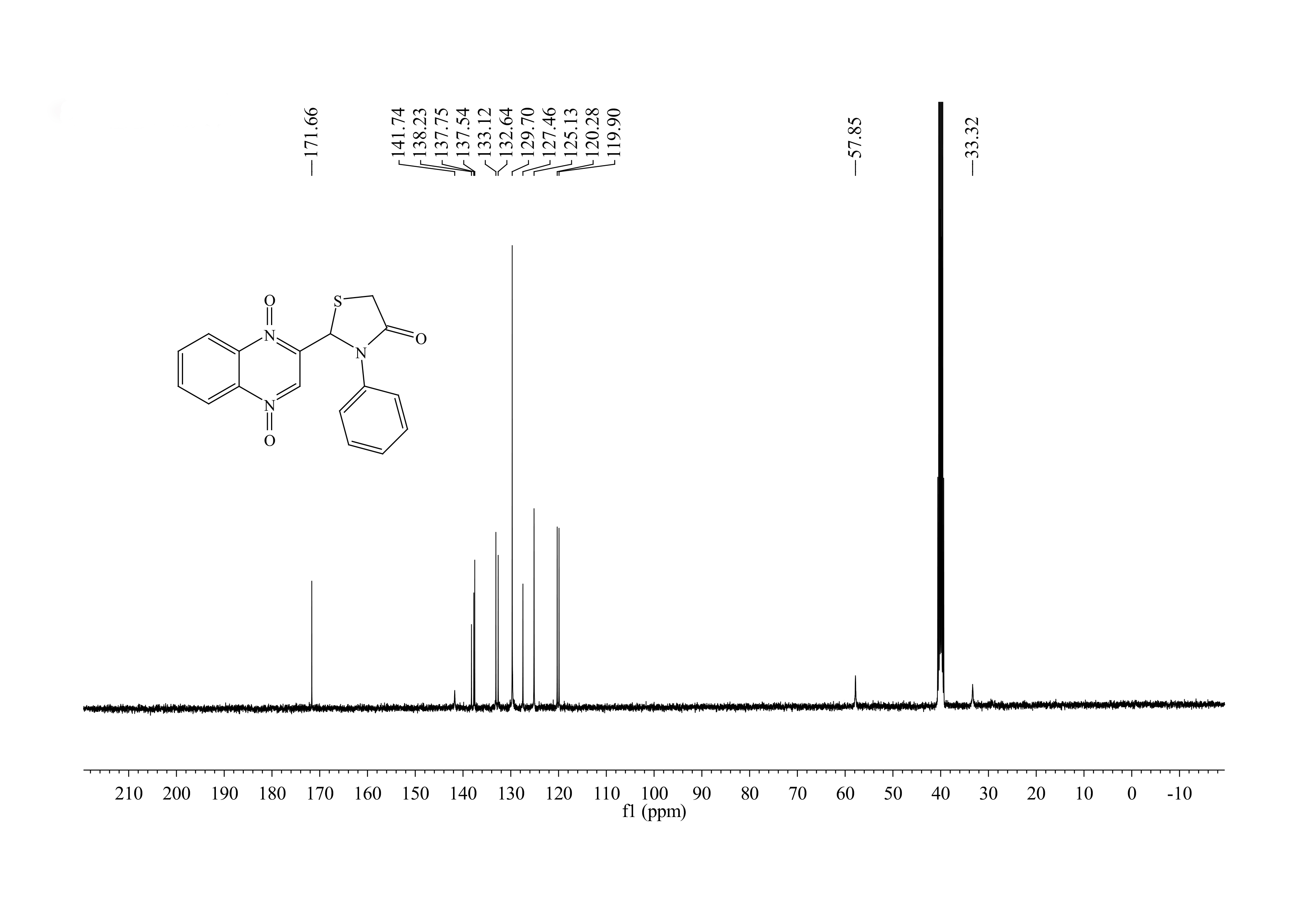


**2b**-^1^H NMR


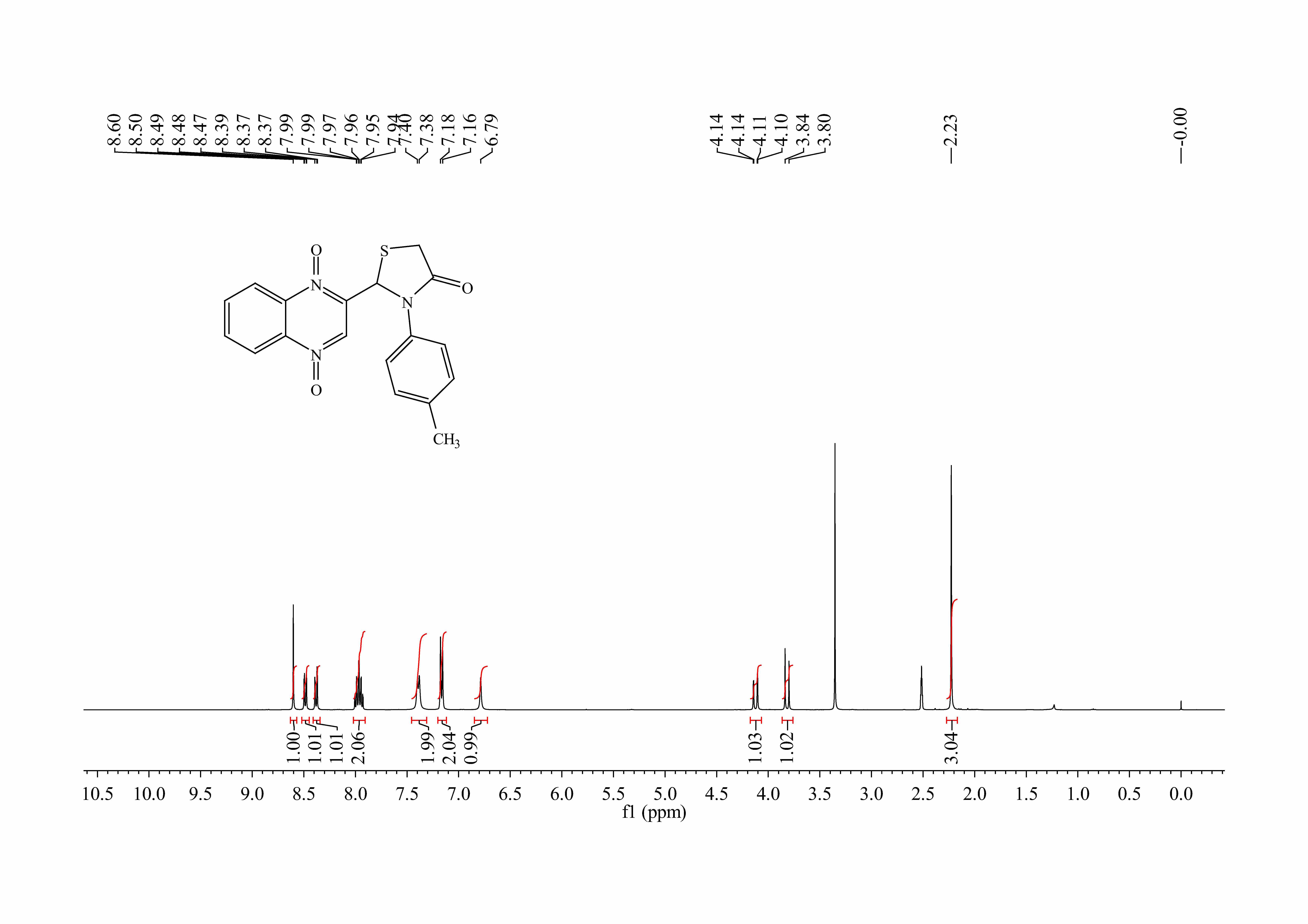


**2b**-^13^C NMR


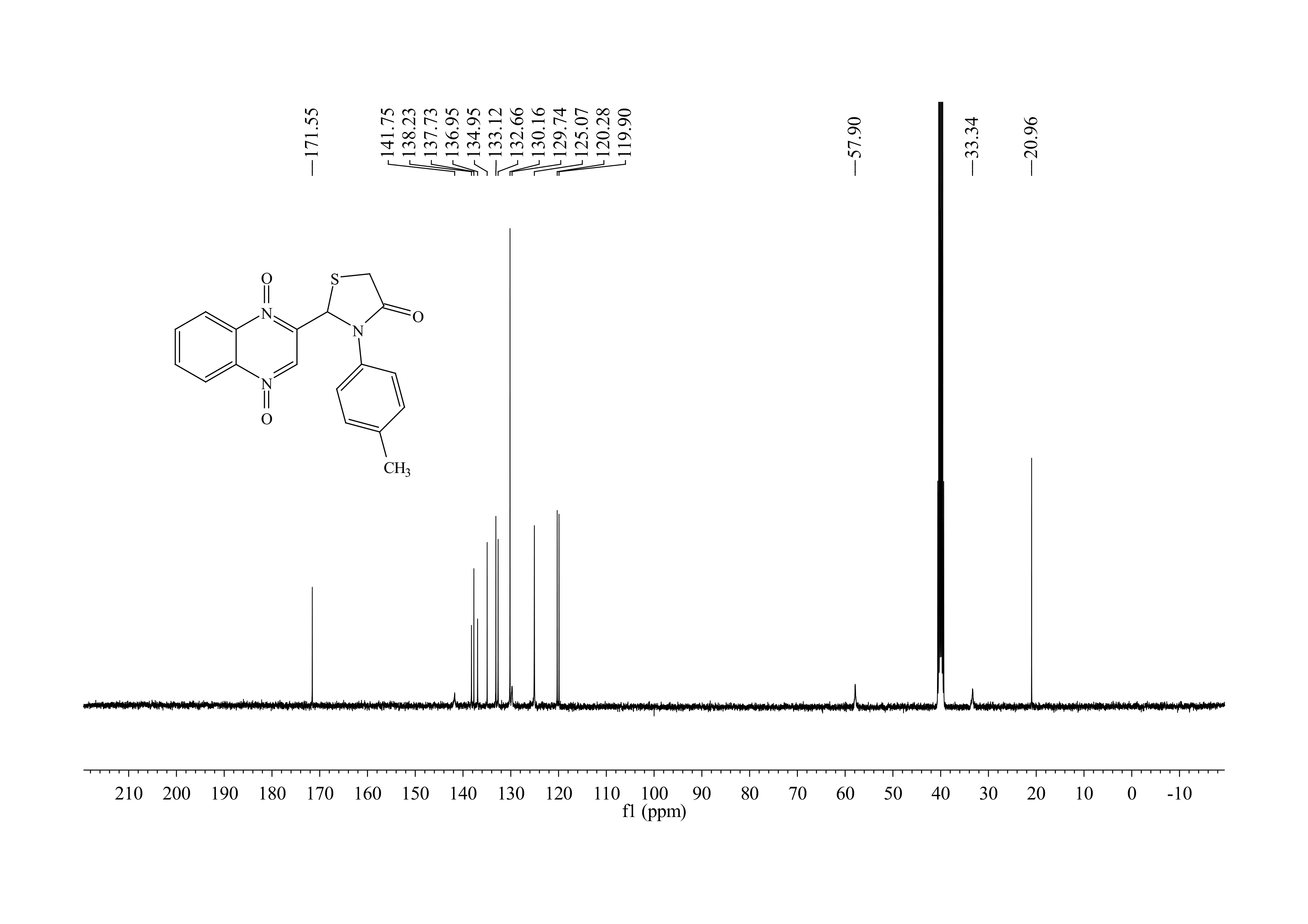


**2c**-^1^H NMR


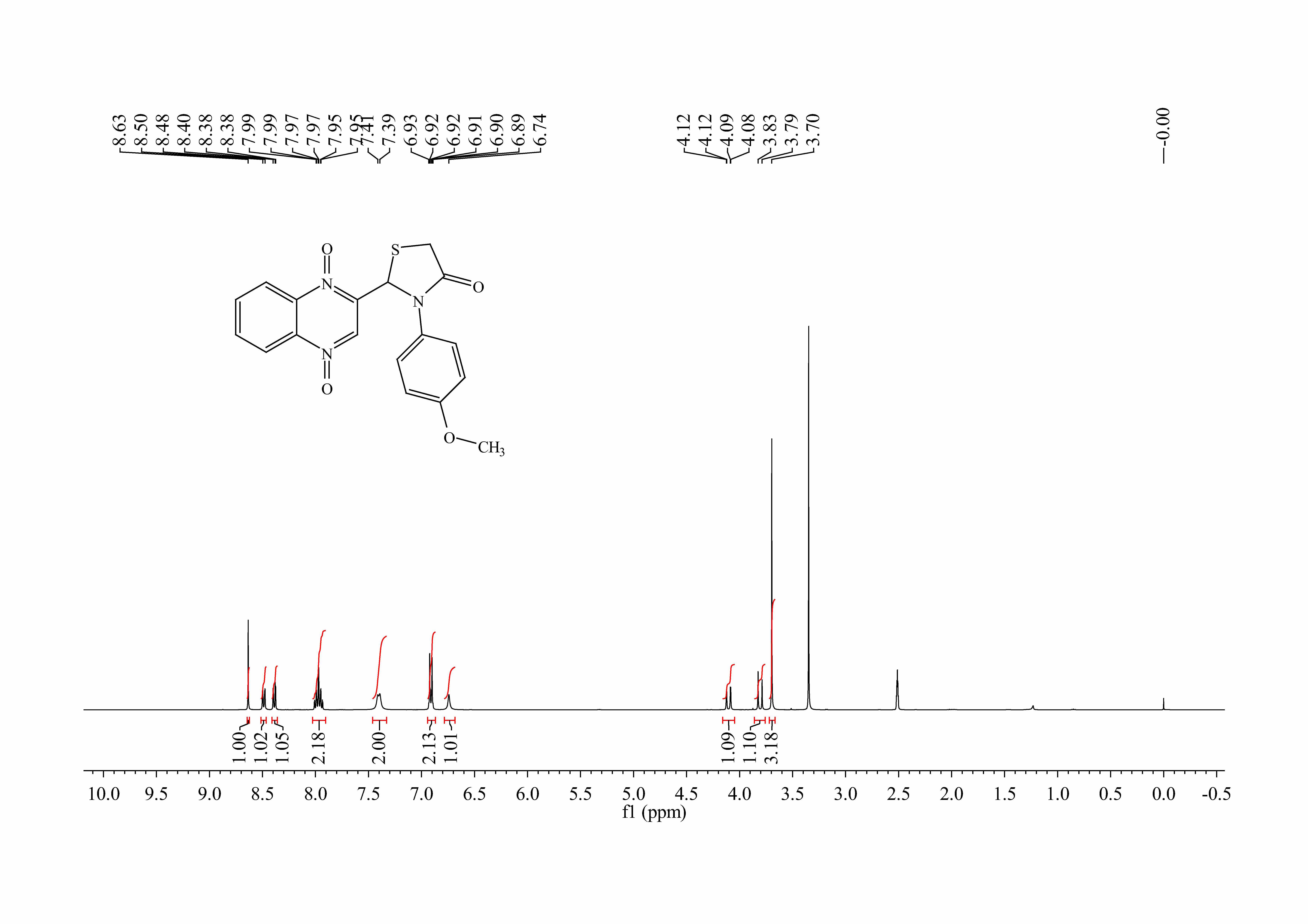


**2c**-^13^C NMR


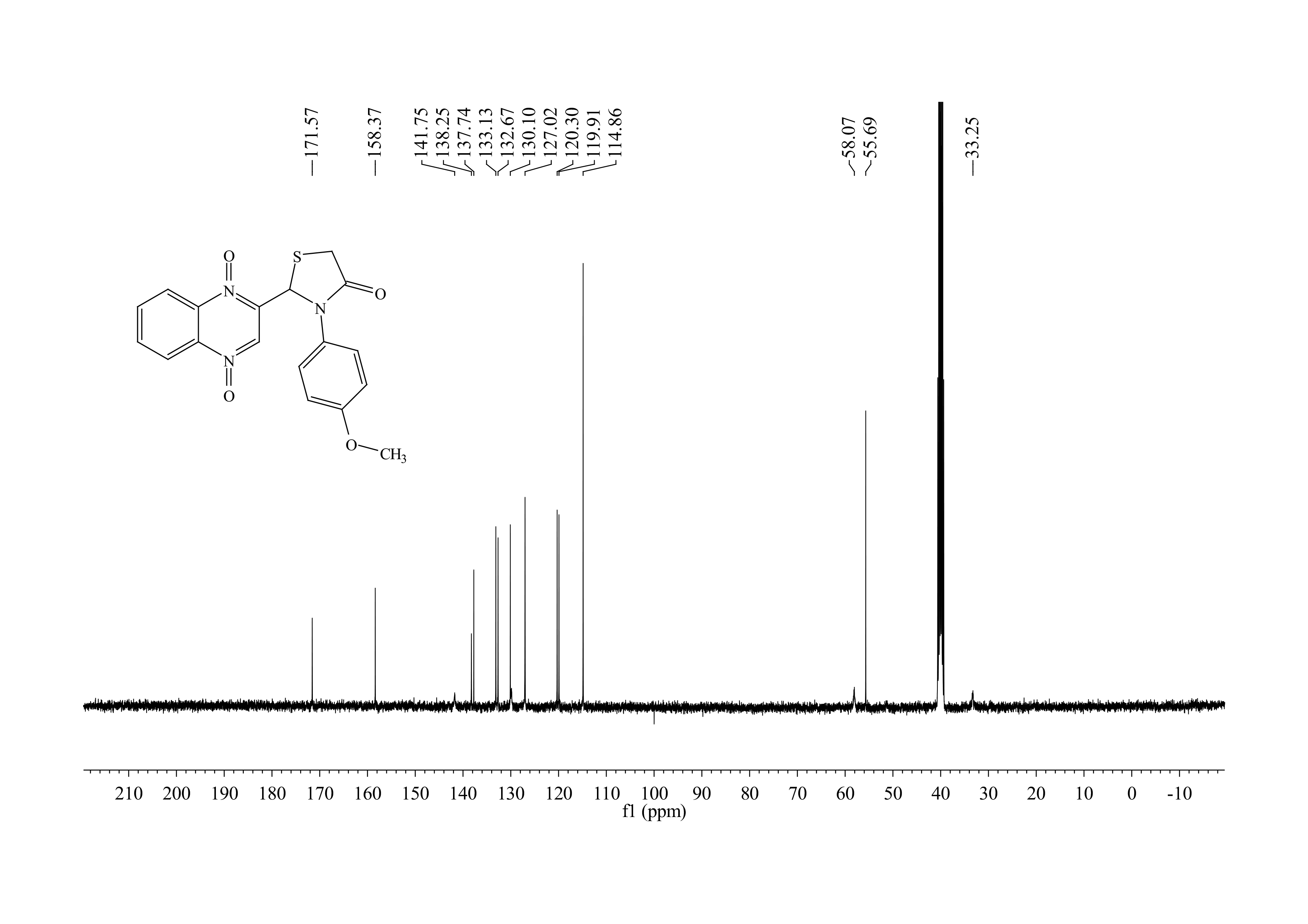


**2d**-^1^H NMR


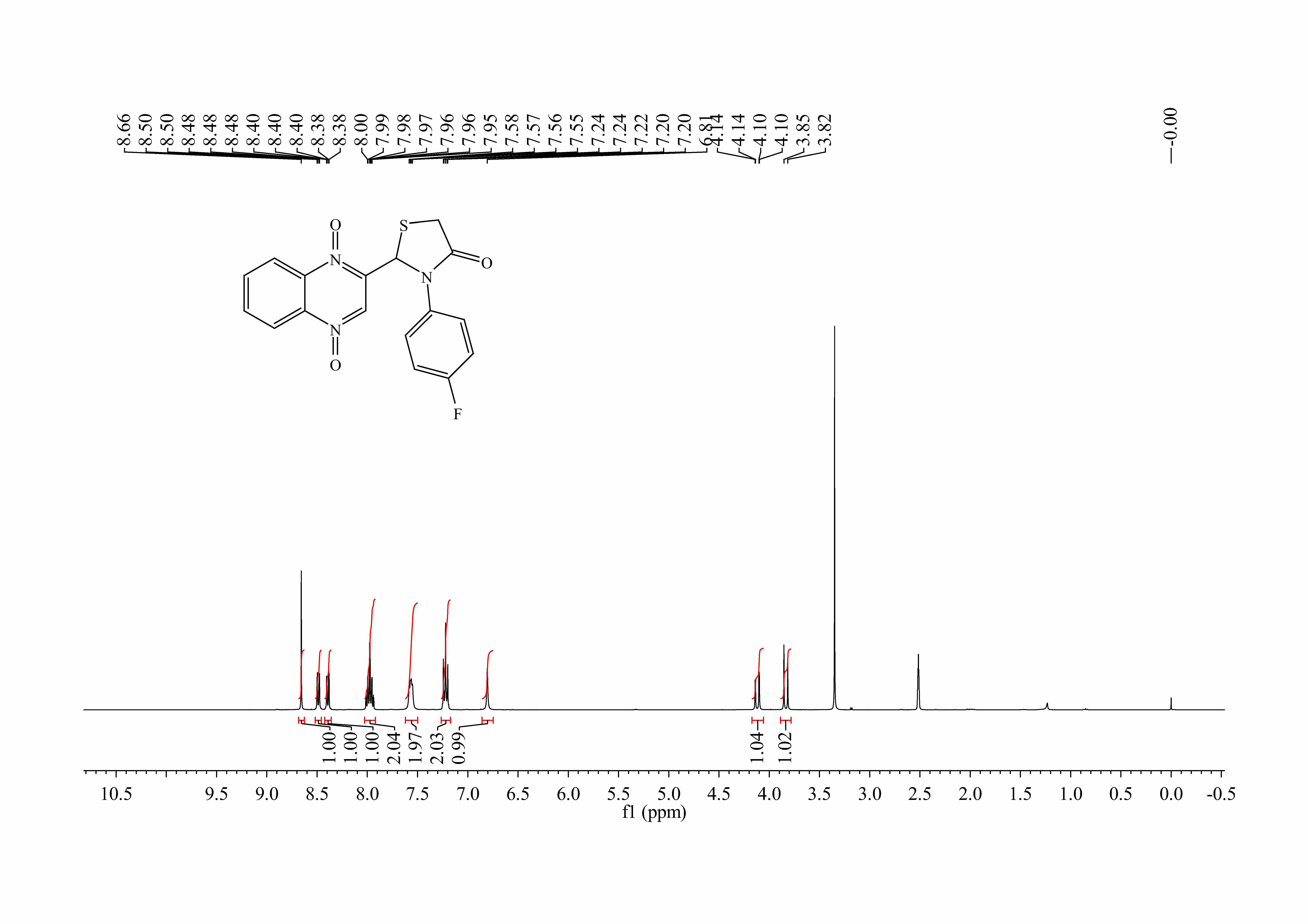


**2d**-^13^C NMR


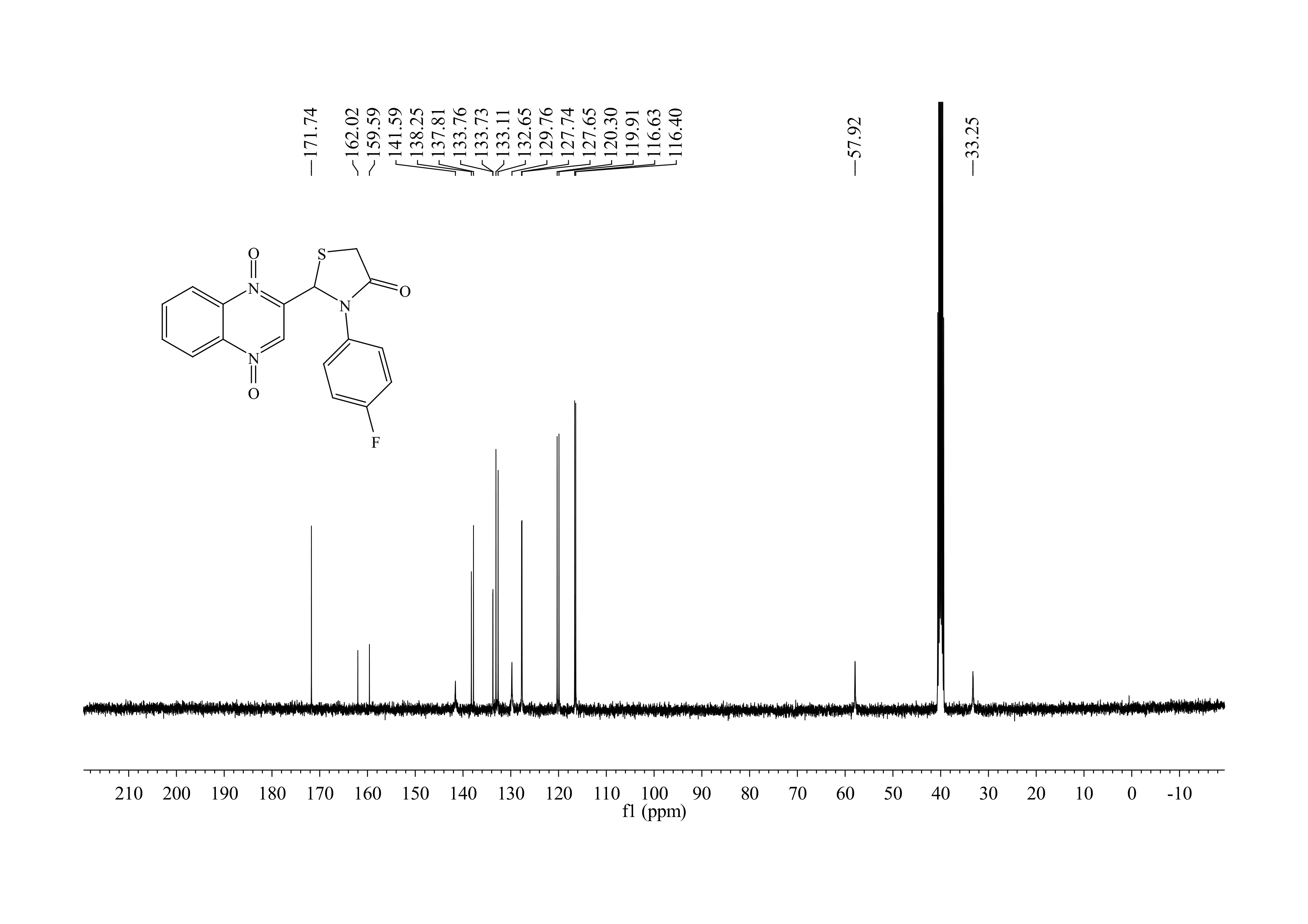


**2e**-^1^H NMR


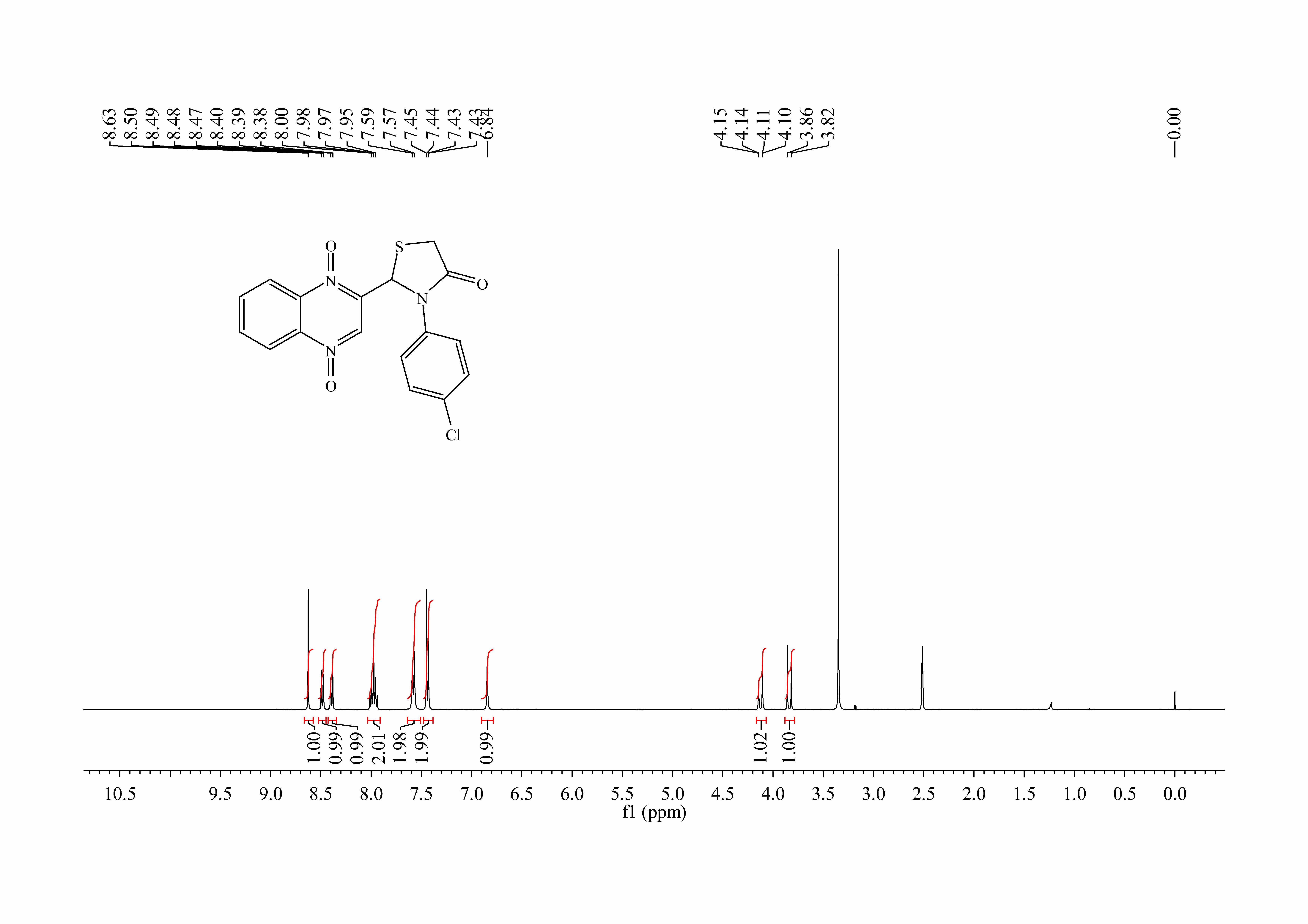


**2e**-^13^C NMR


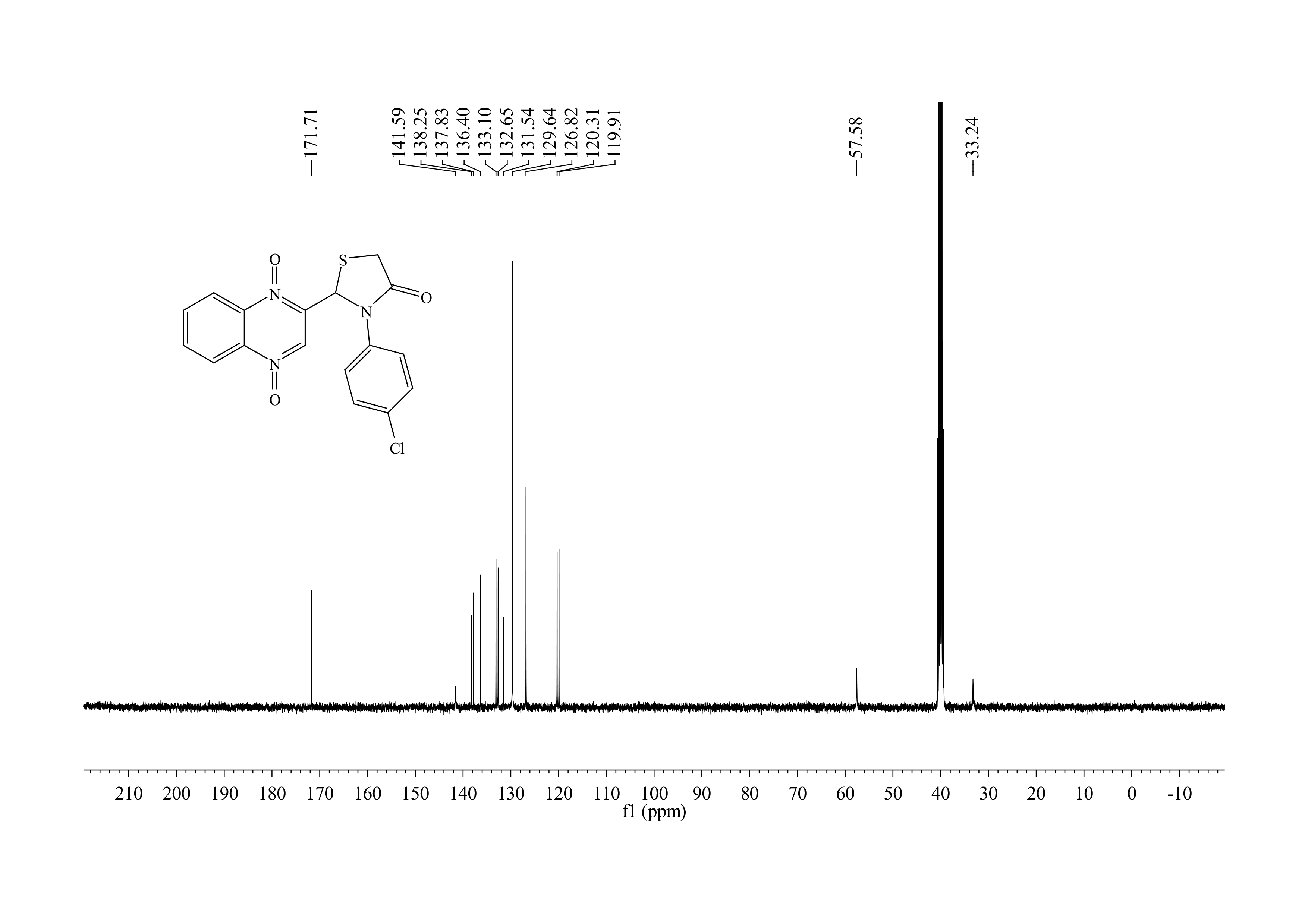


**2f**-^1^H NMR


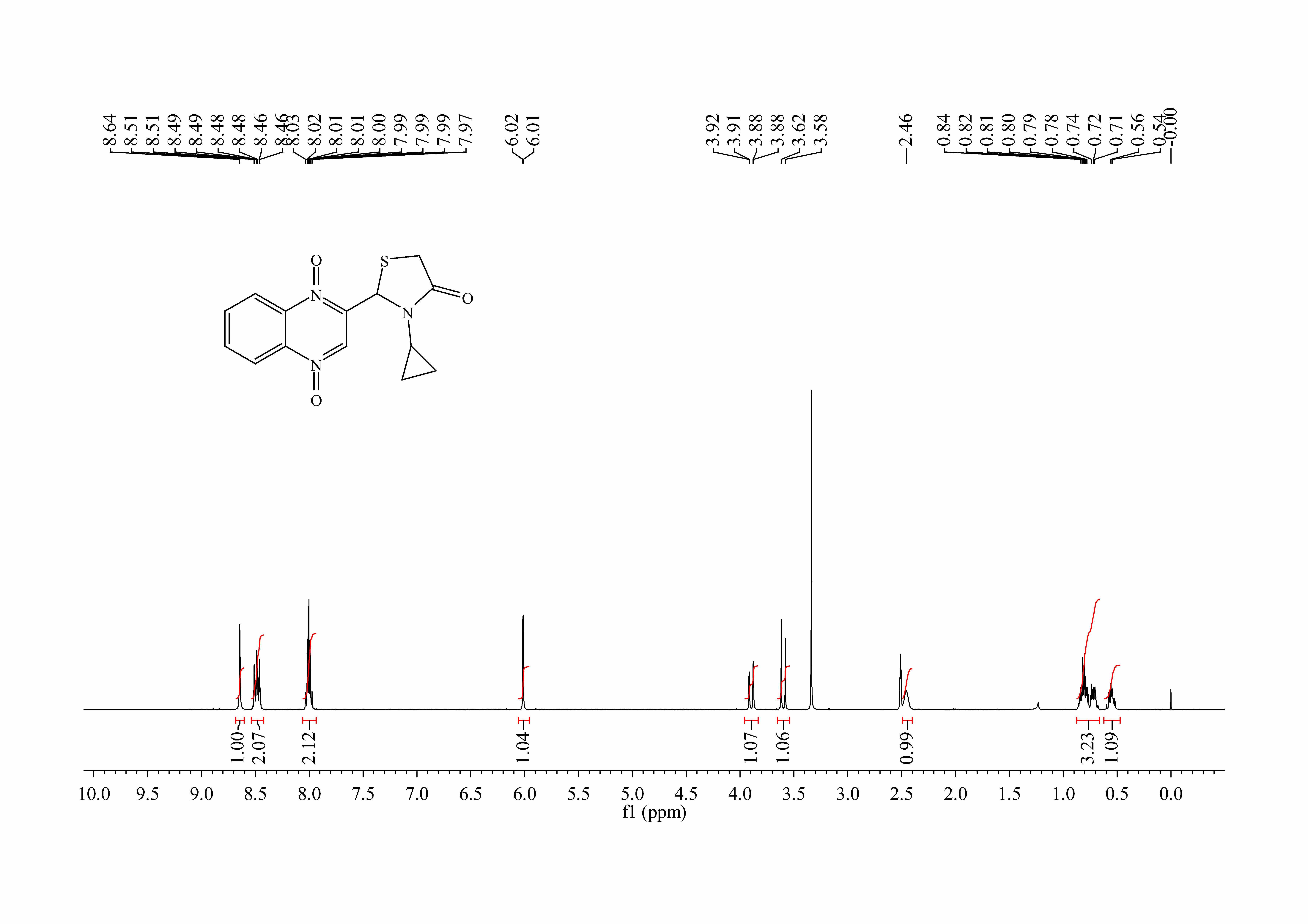


**2f**-^13^C NMR


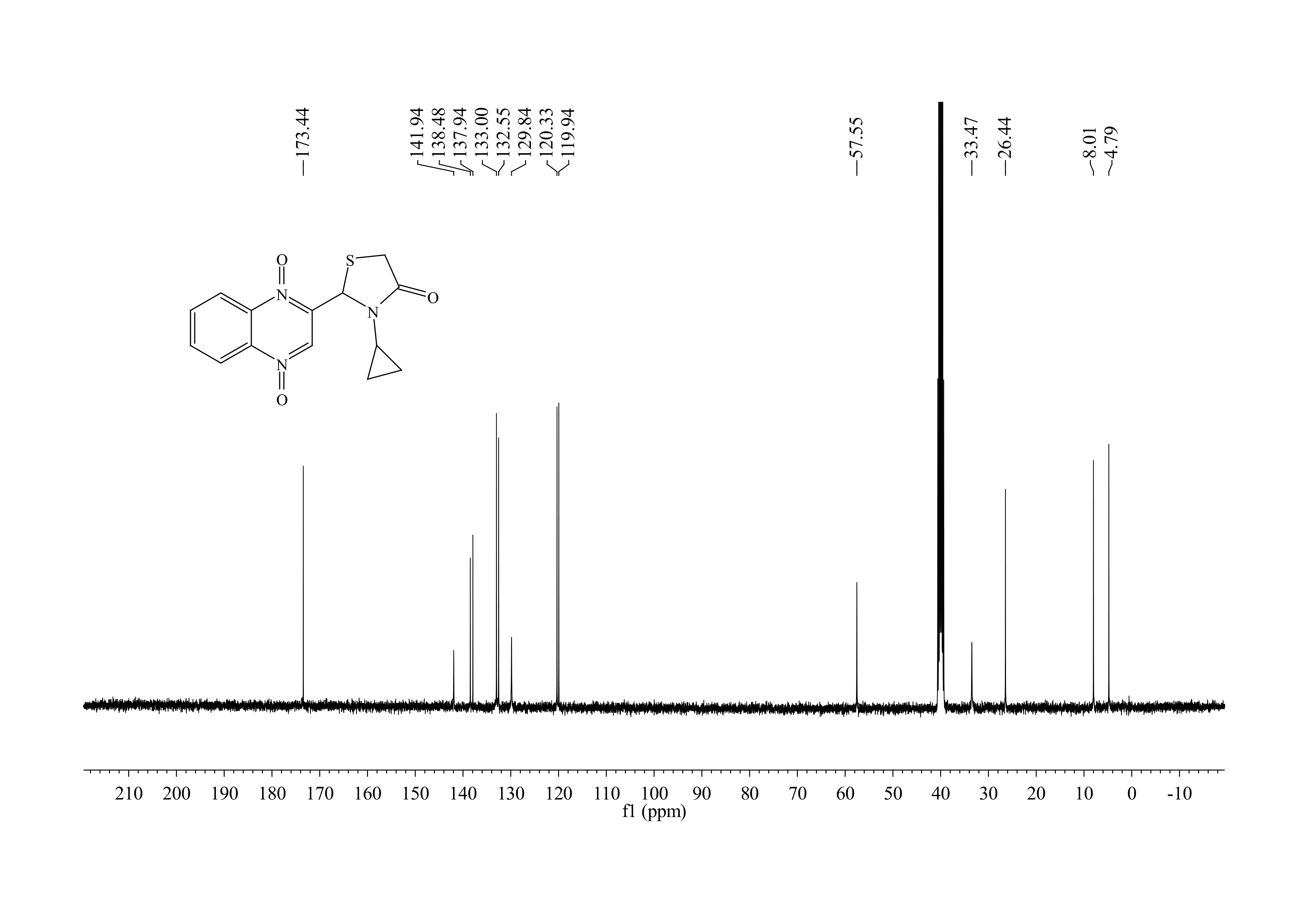


**2g**-^1^H NMR


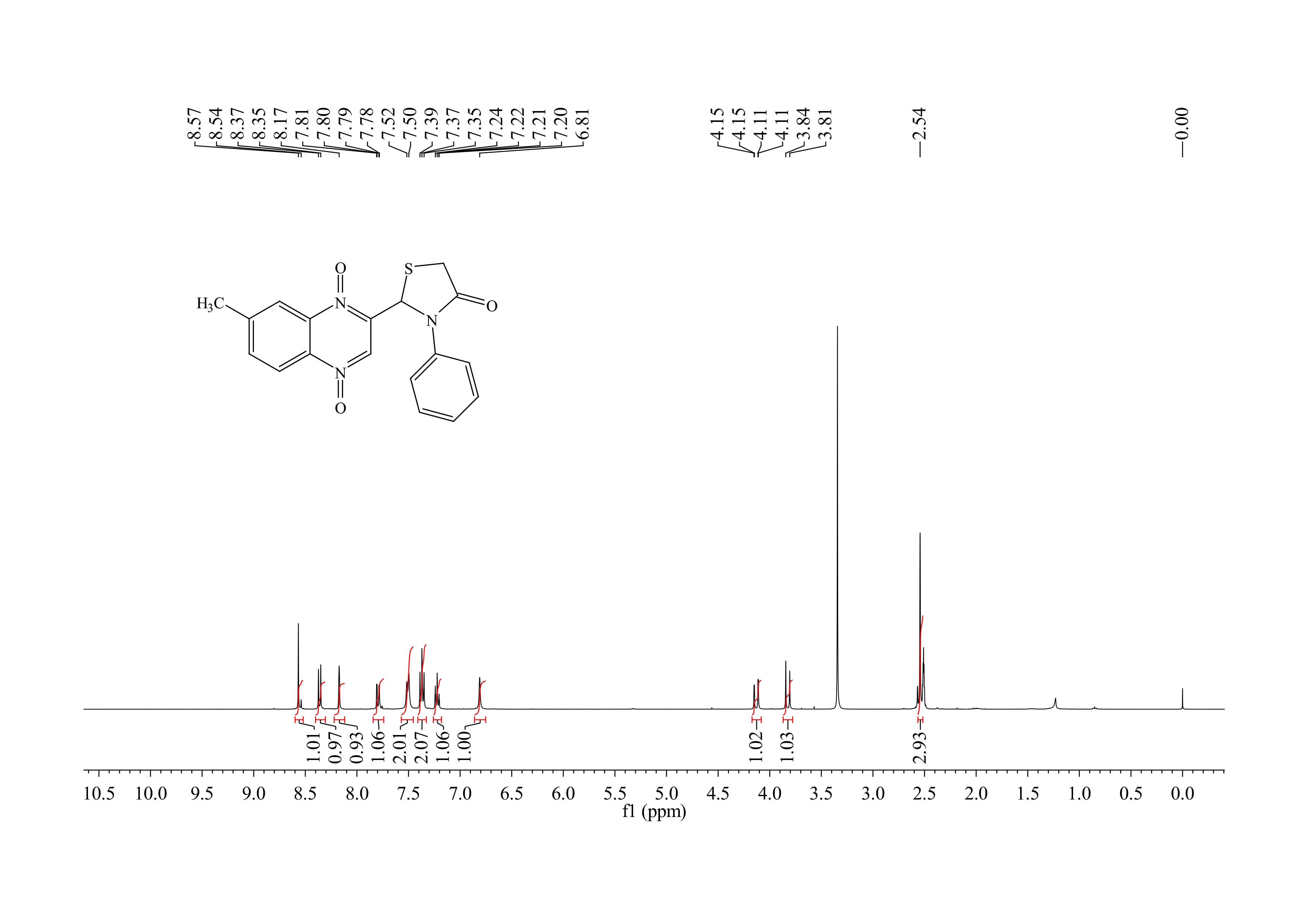


**2g**-^13^C NMR


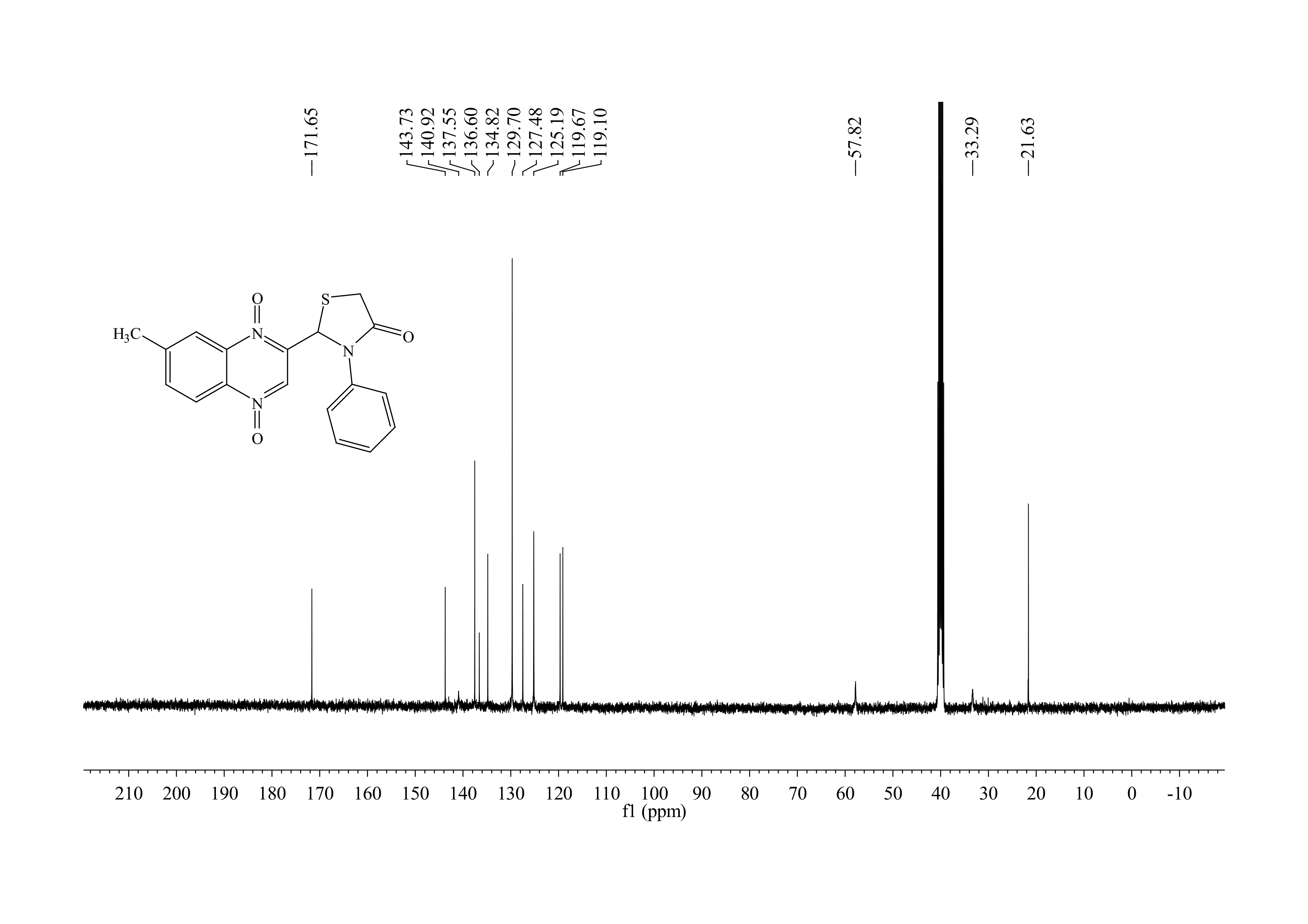


**2h**-^1^H NMR


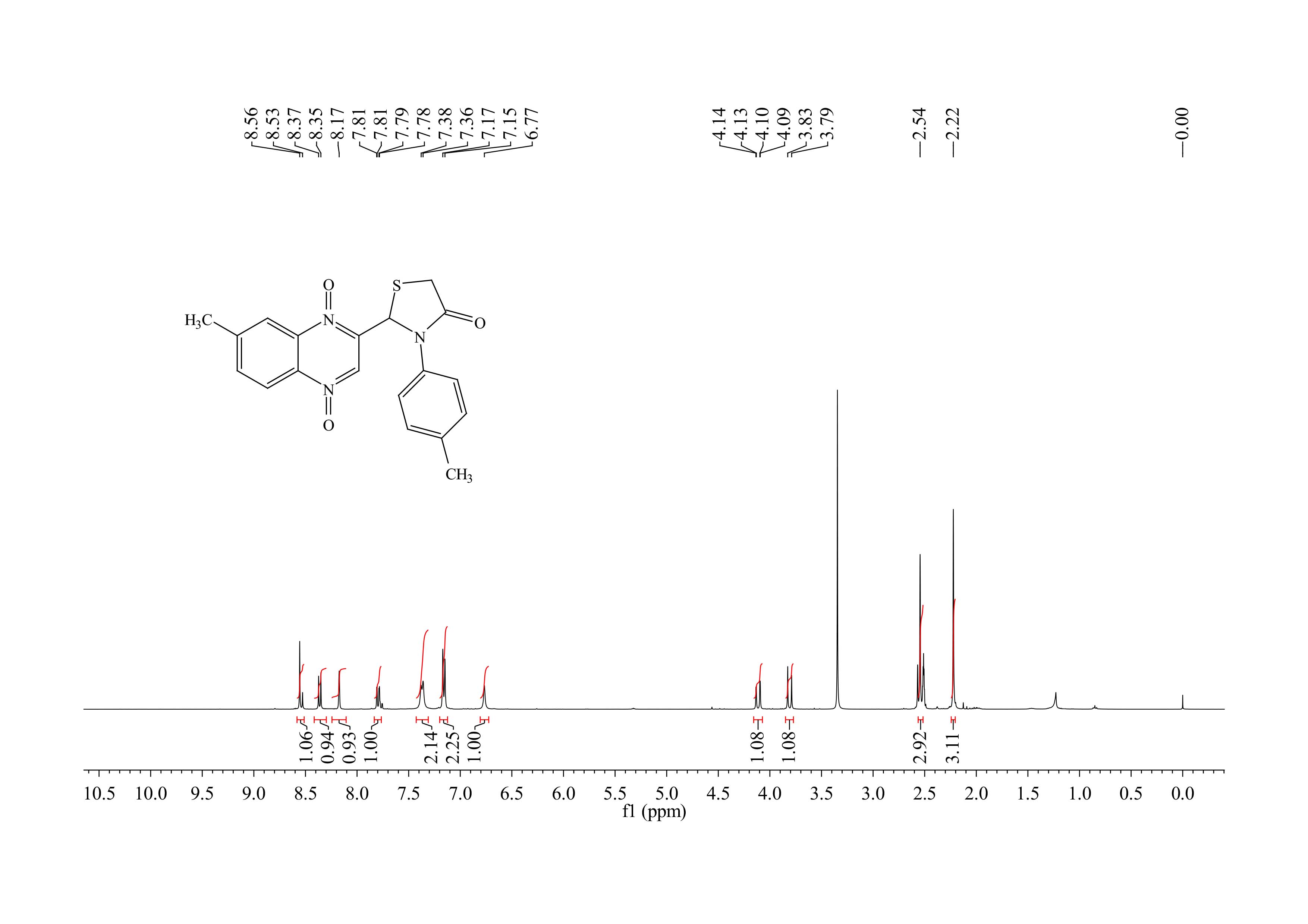


**2h**-^13^C NMR


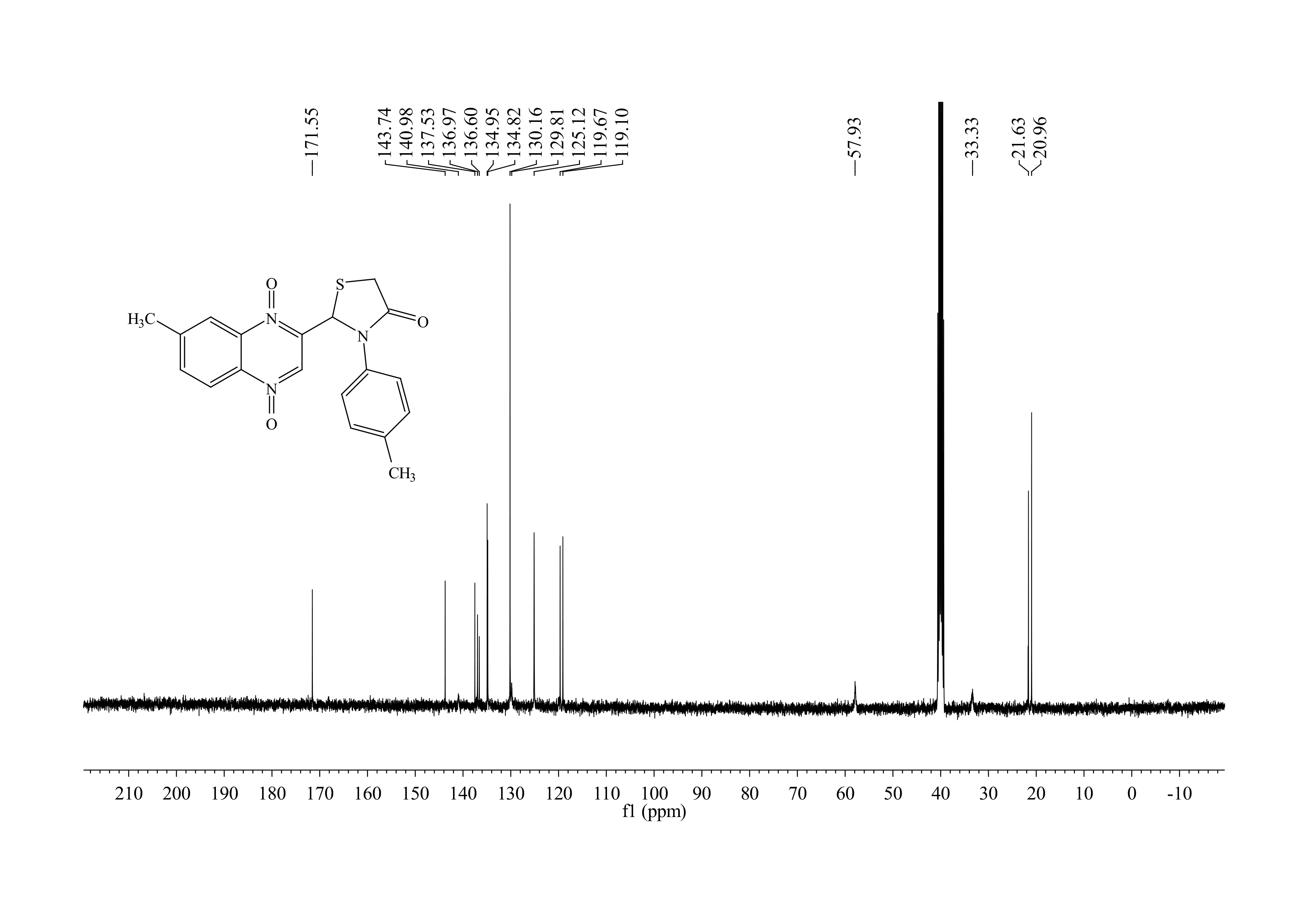


**2i**-^1^H NMR


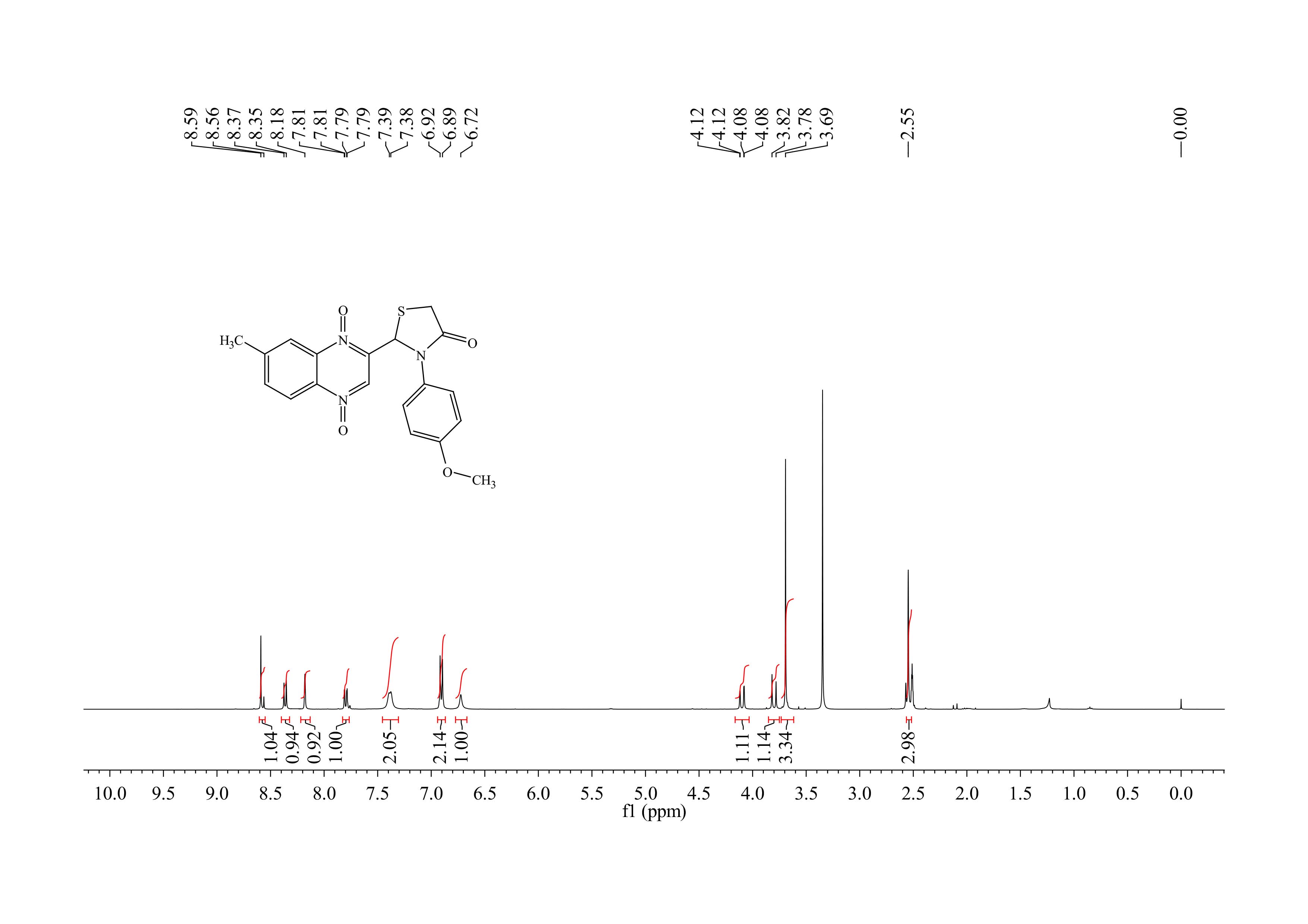


**2i**-^13^C NMR


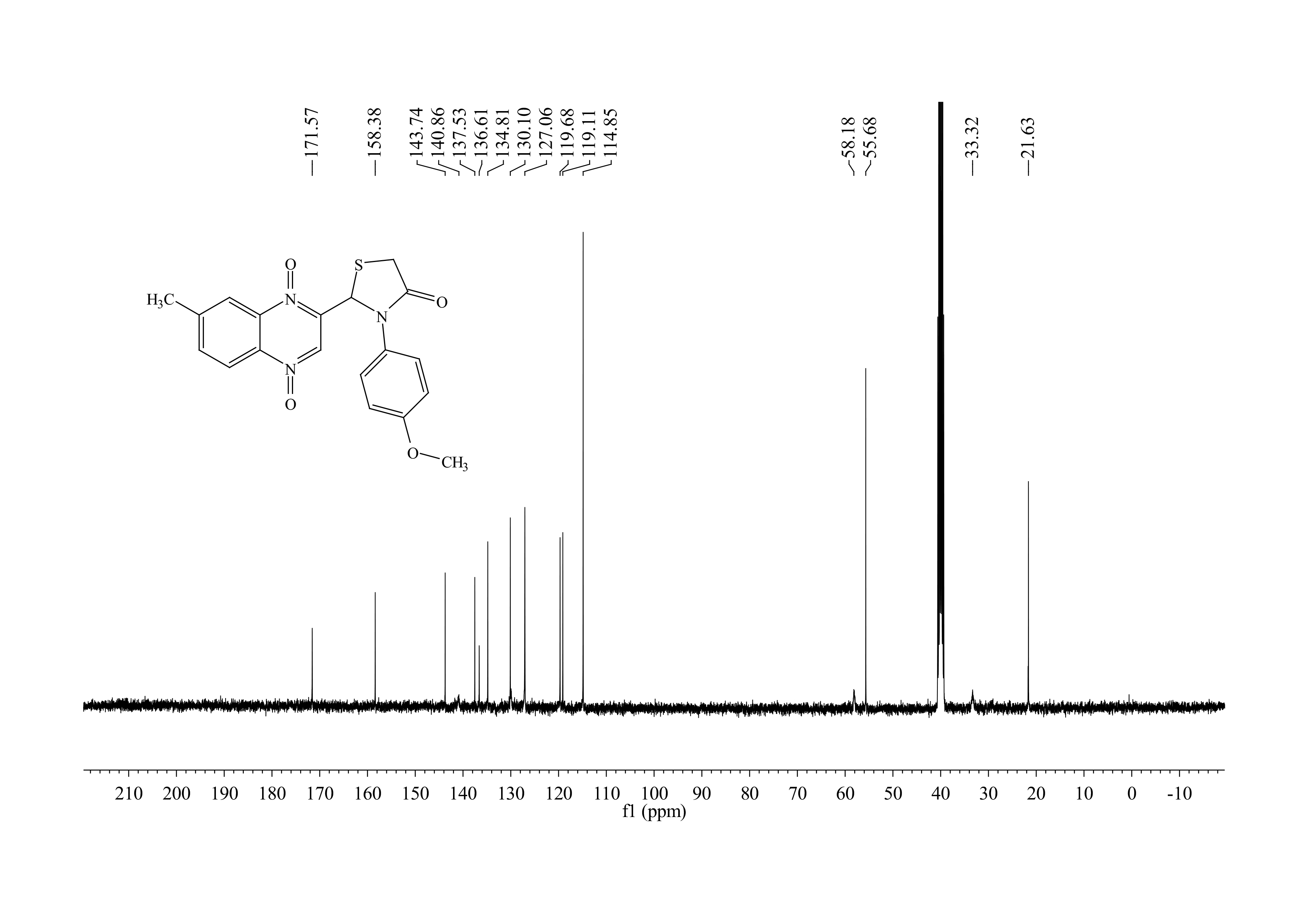


**2j**-^1^H NMR


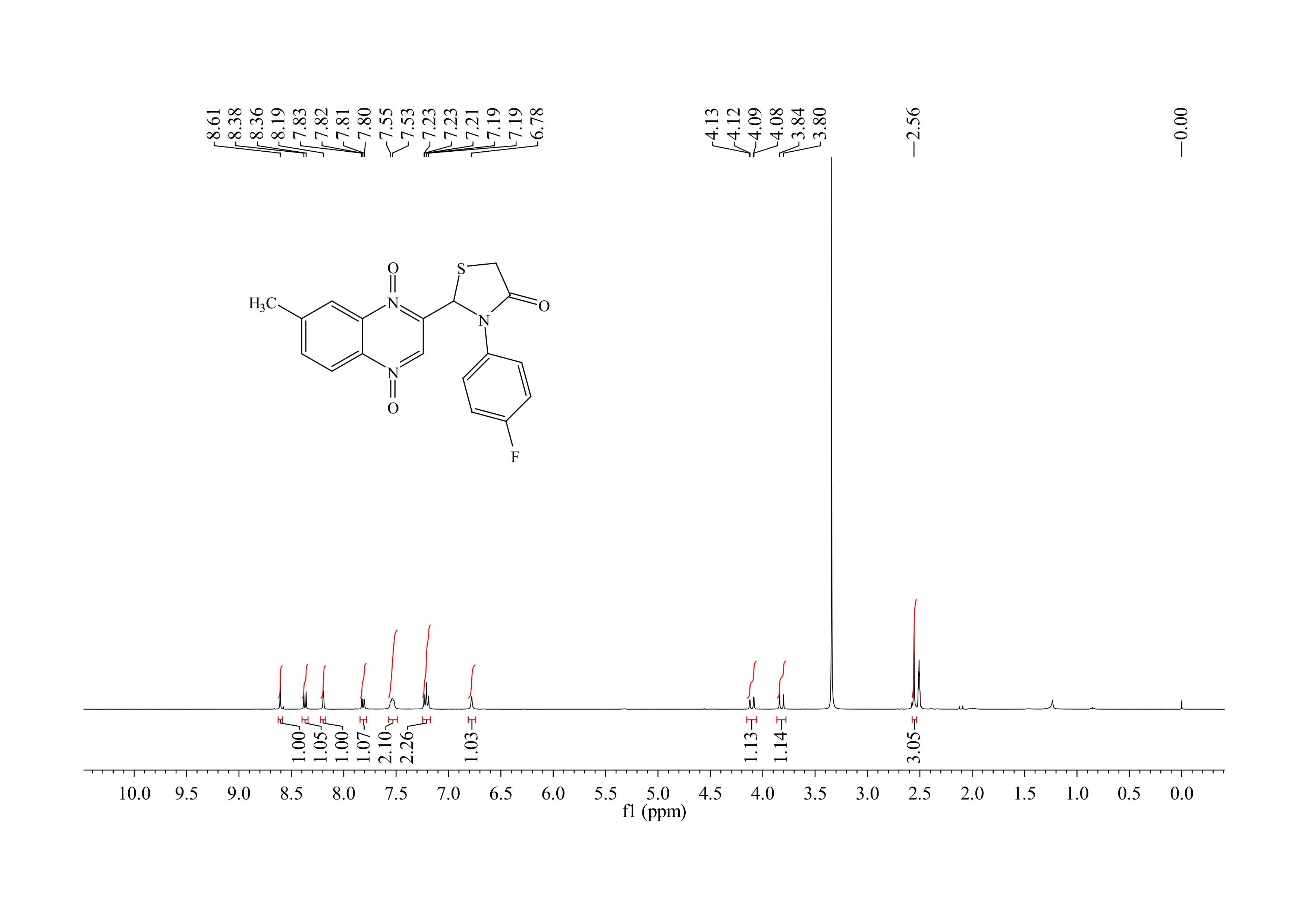


**2j**-^13^C NMR


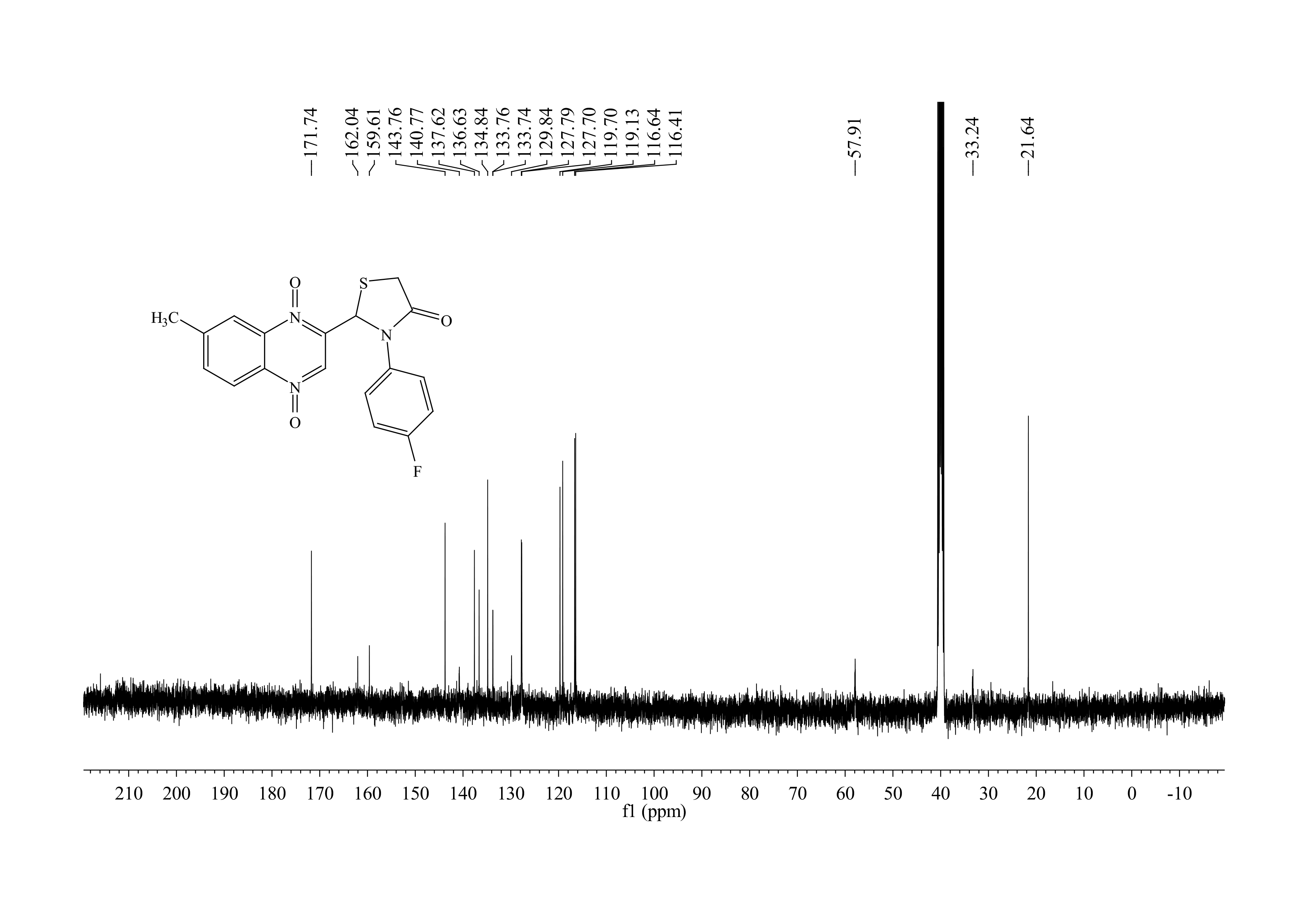


**2k**-^1^H NMR


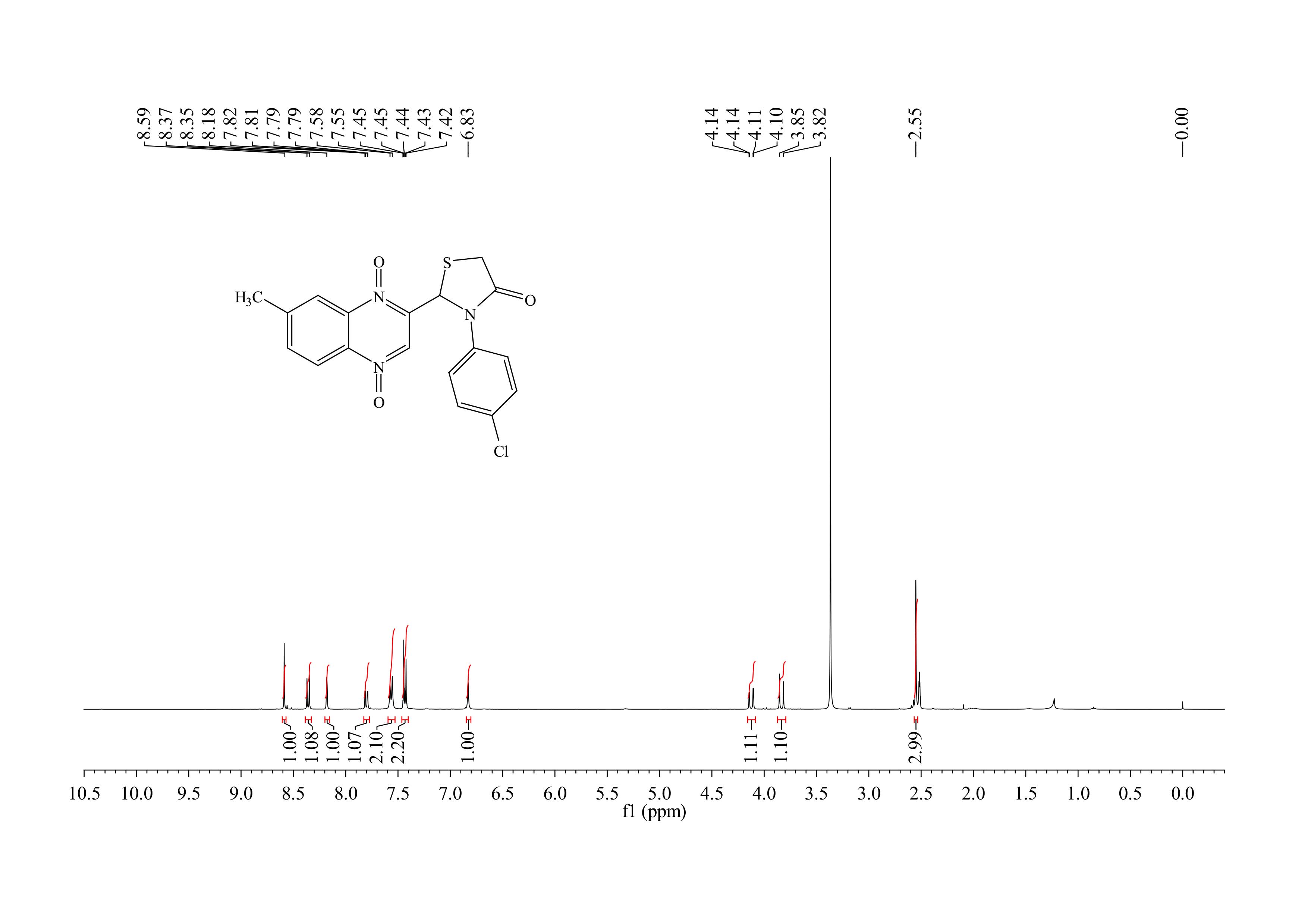


**2k**-^13^C NMR


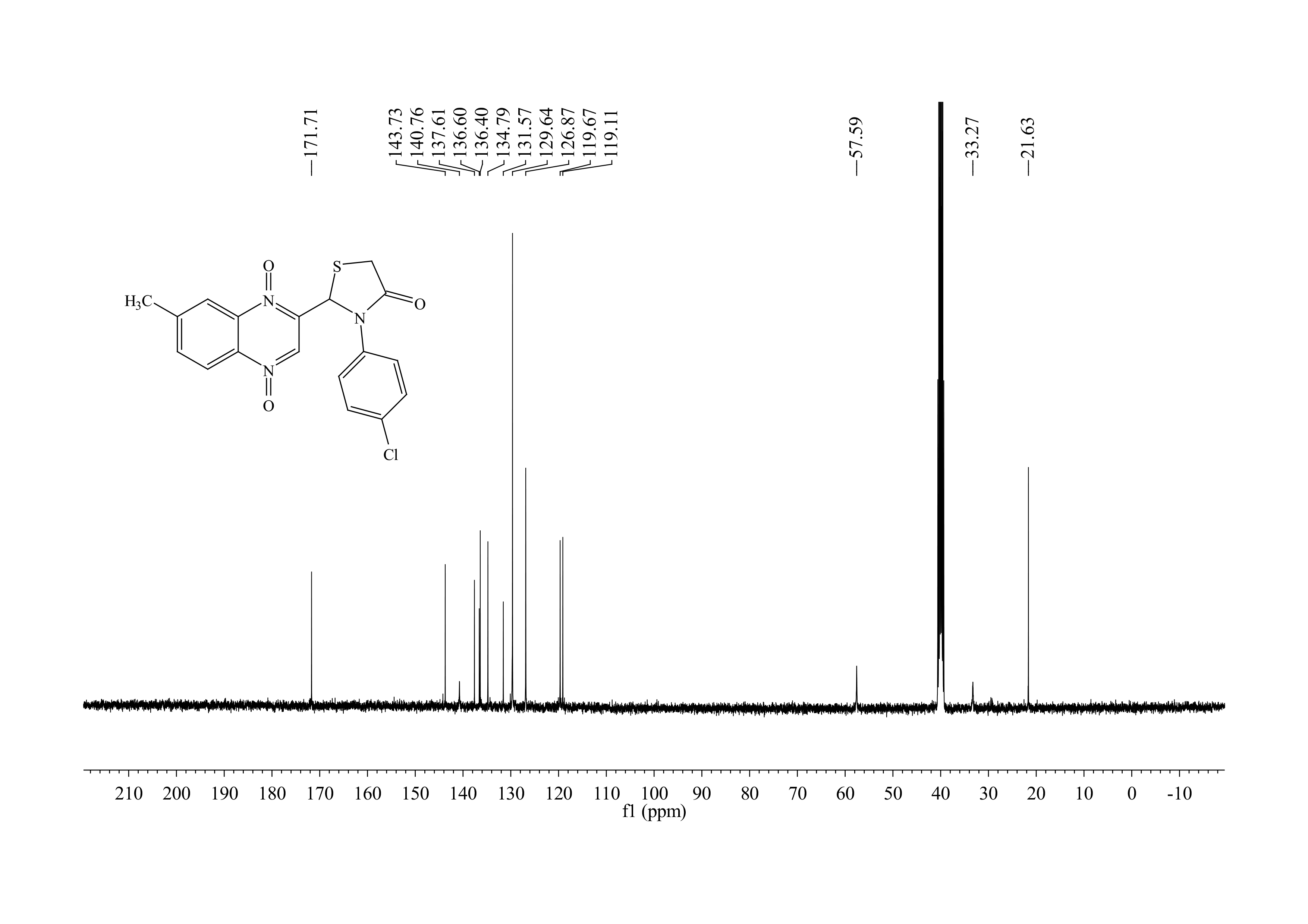


**2l**-^1^H NMR


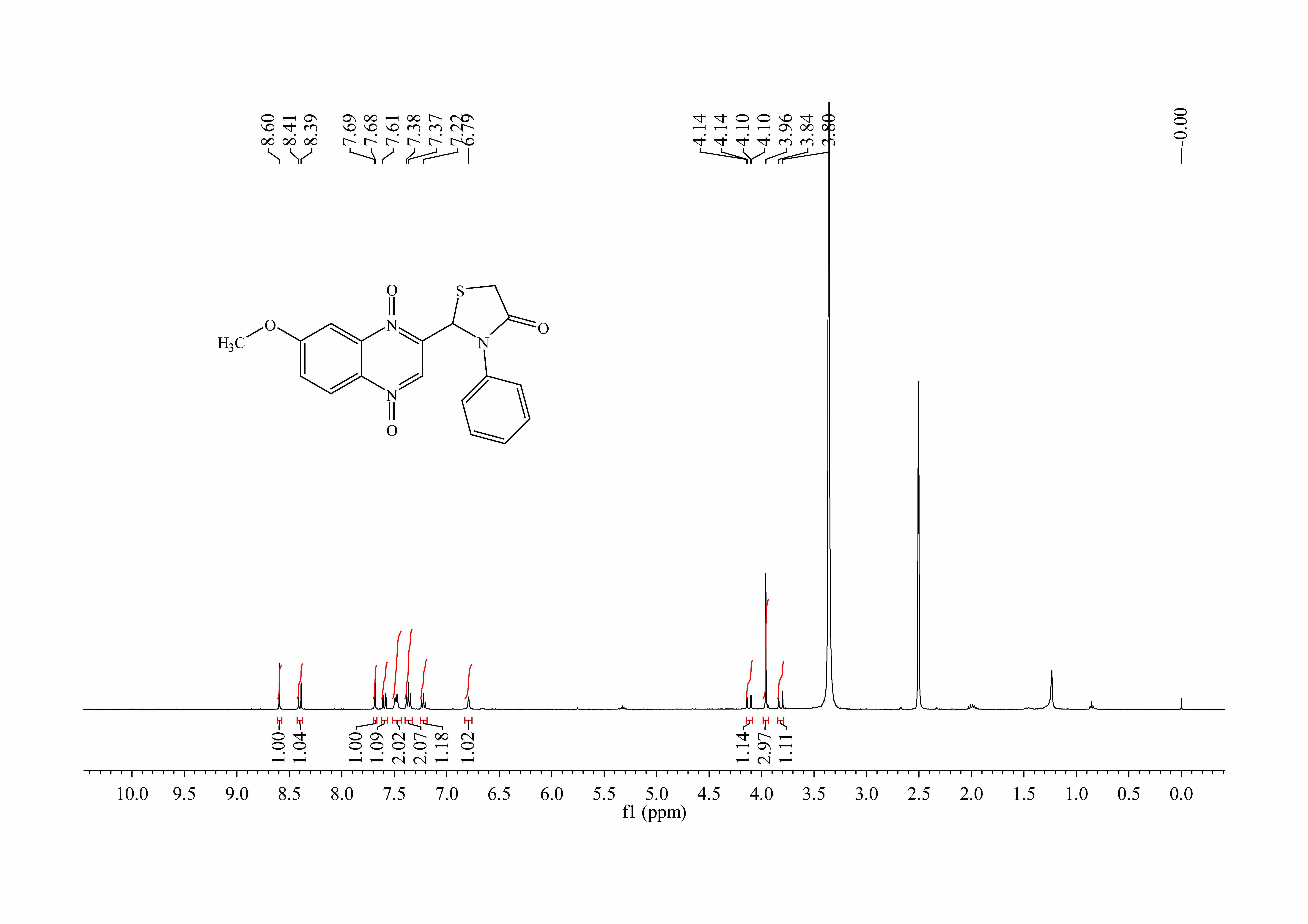


**2l**-^13^C NMR


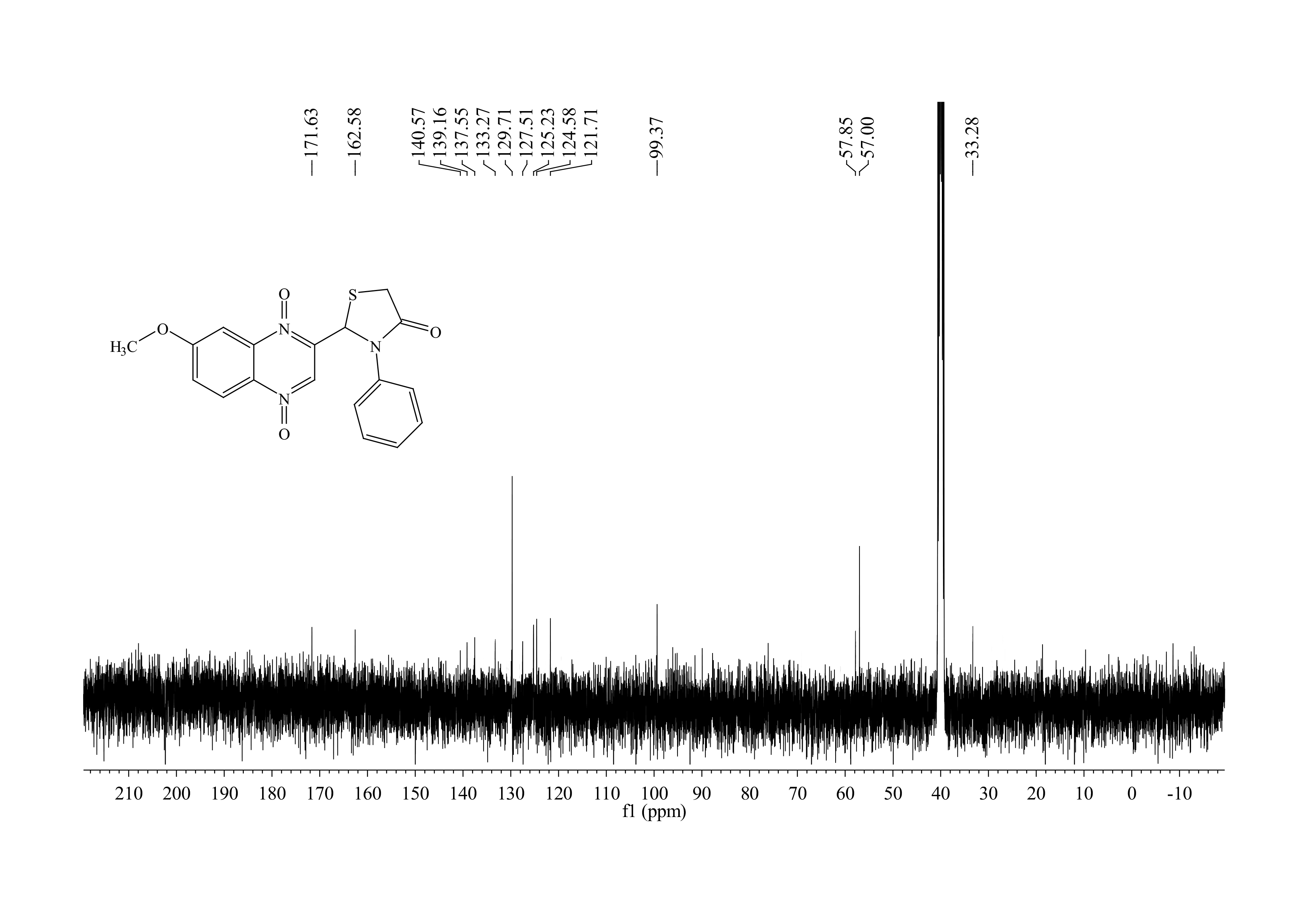


**2m**-^1^H NMR


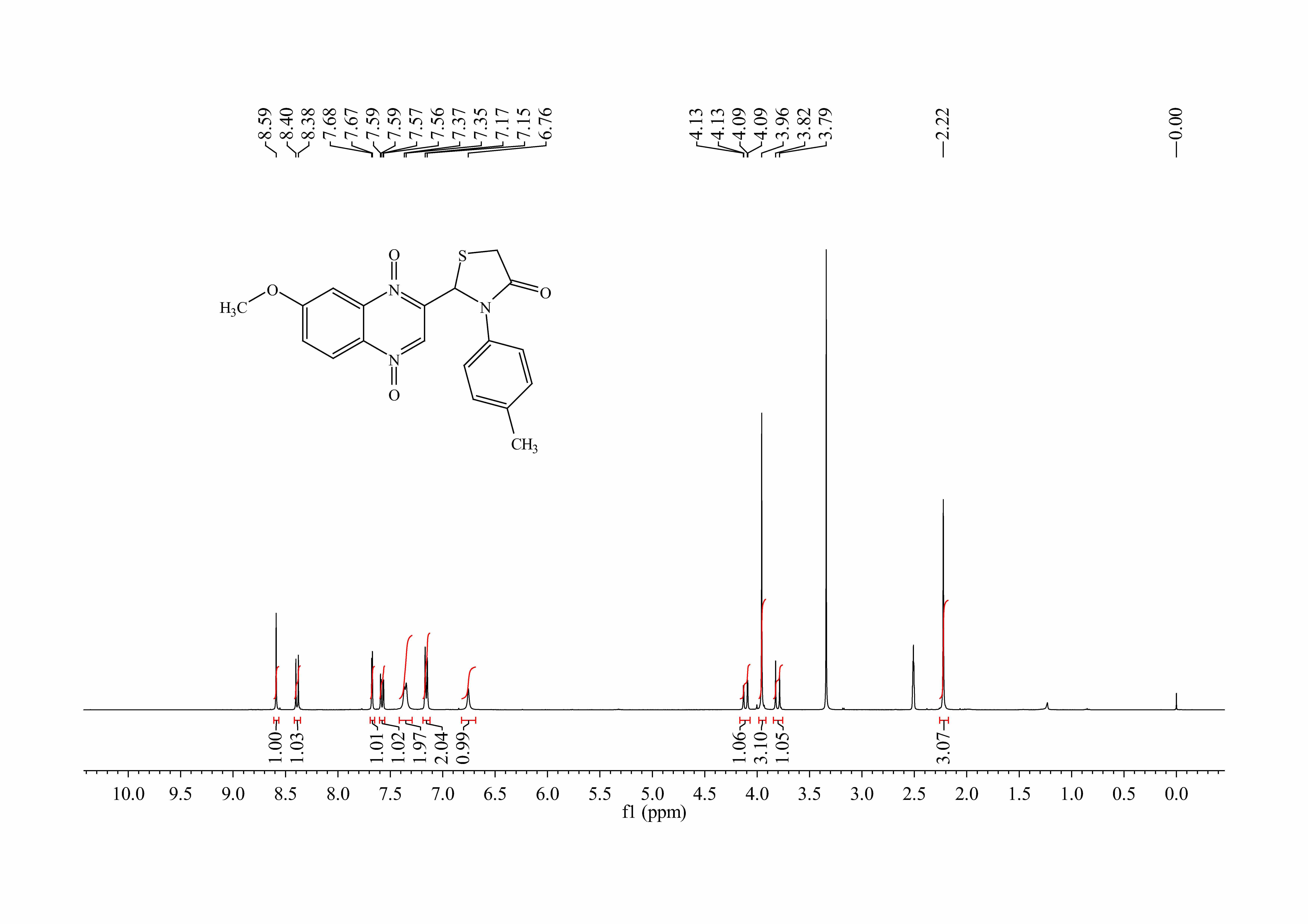


**2m**-^13^C NMR


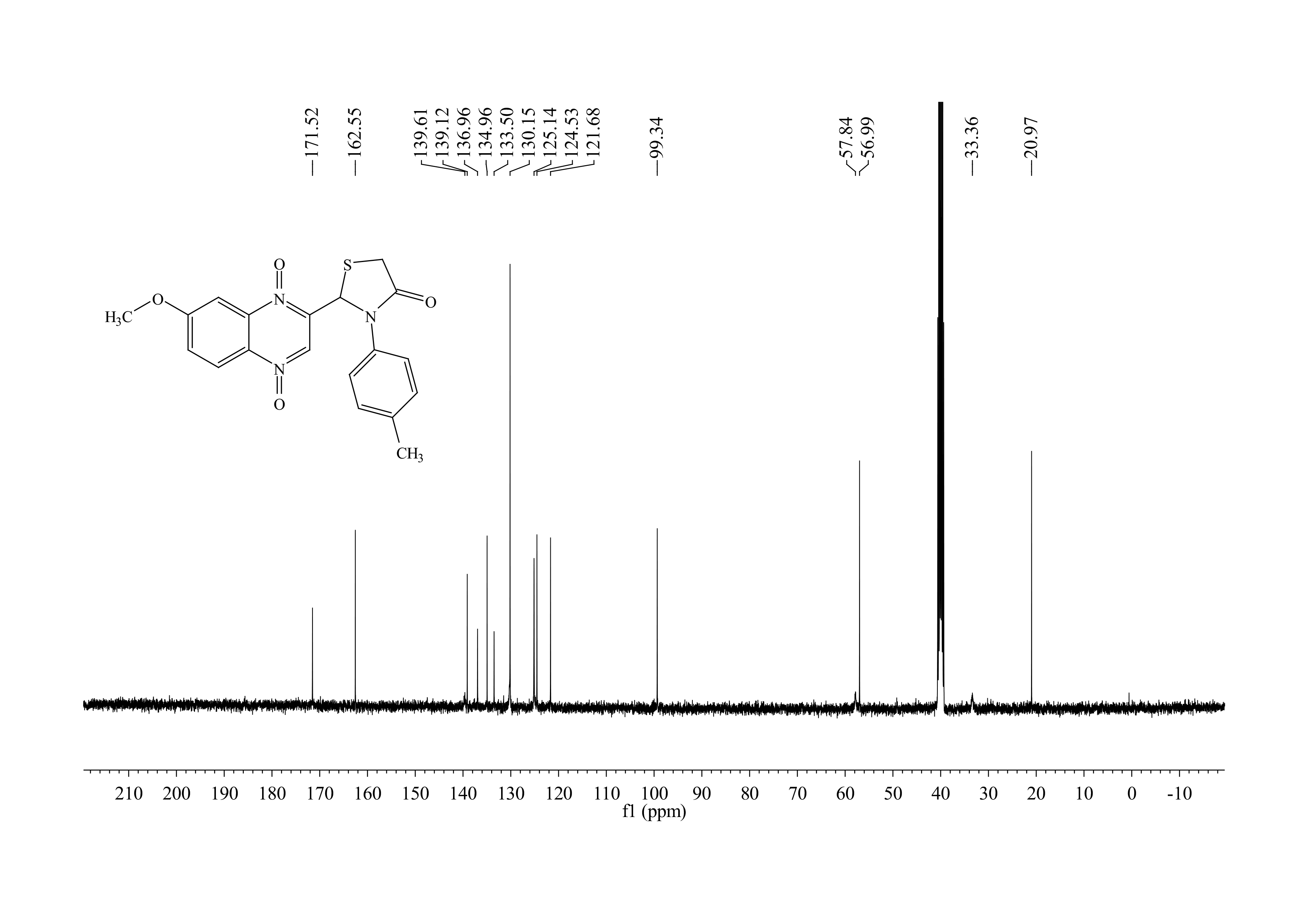


**2n**-^1^H NMR


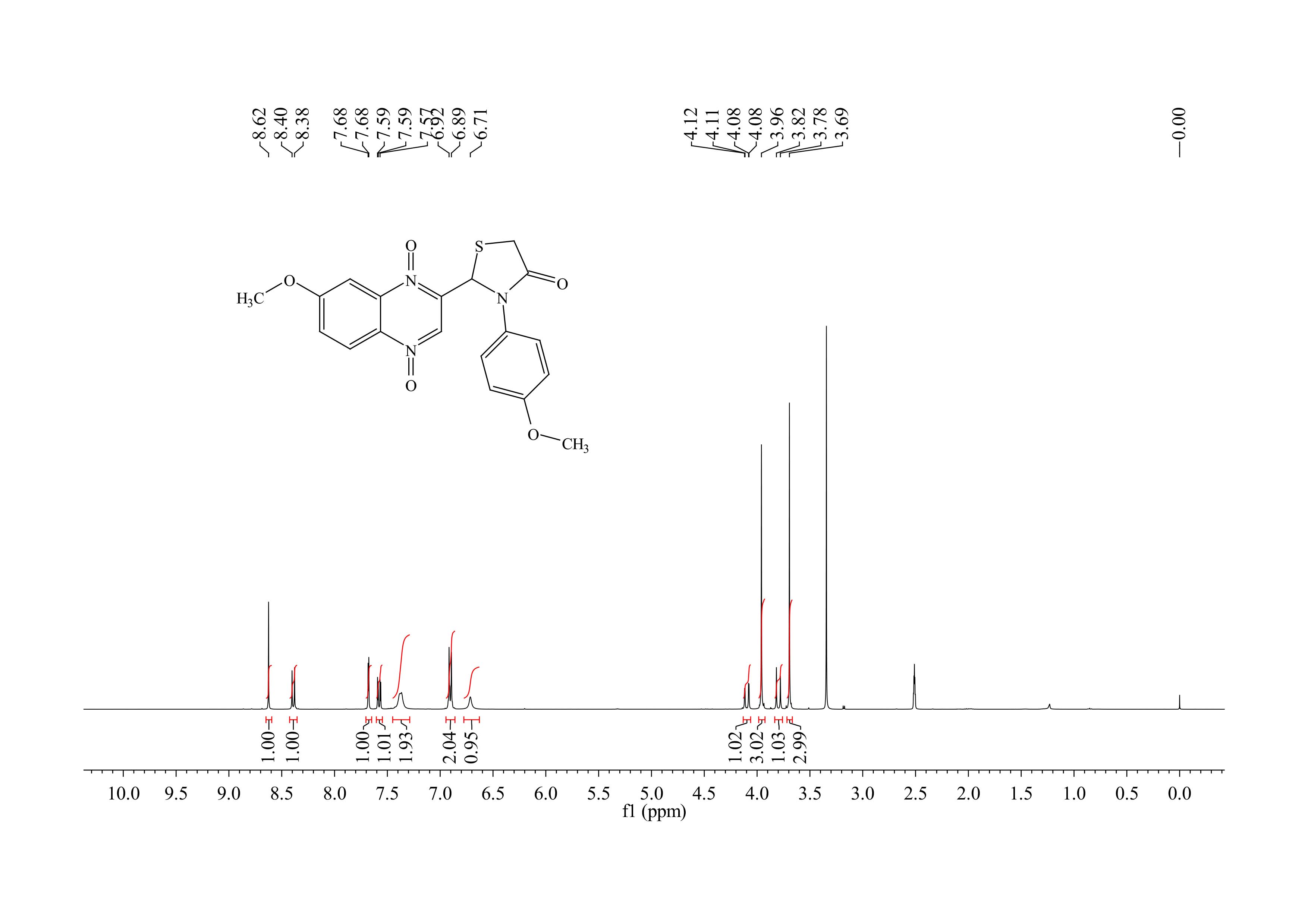


**2n**-^13^C NMR


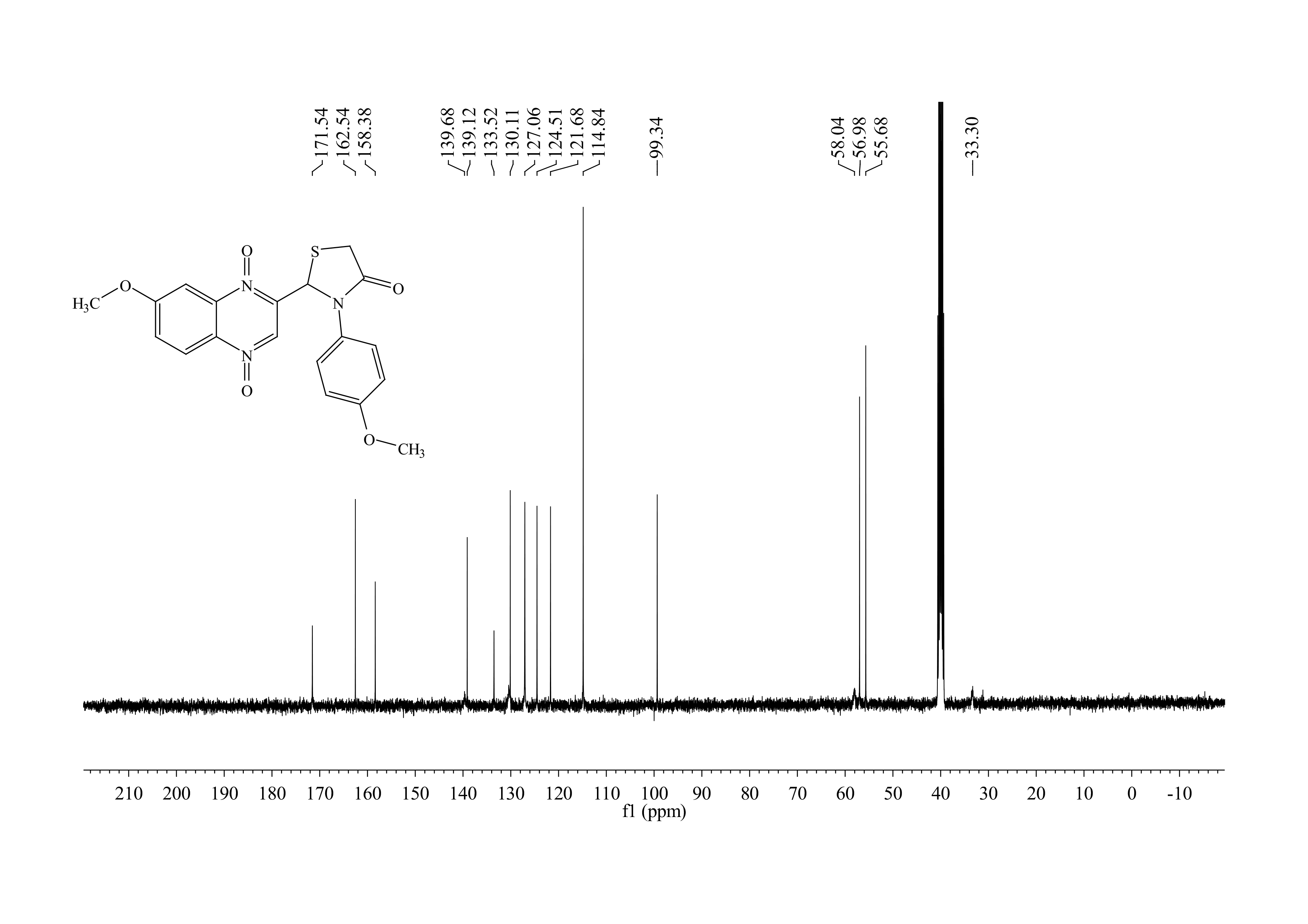


**2o**-^1^H NMR


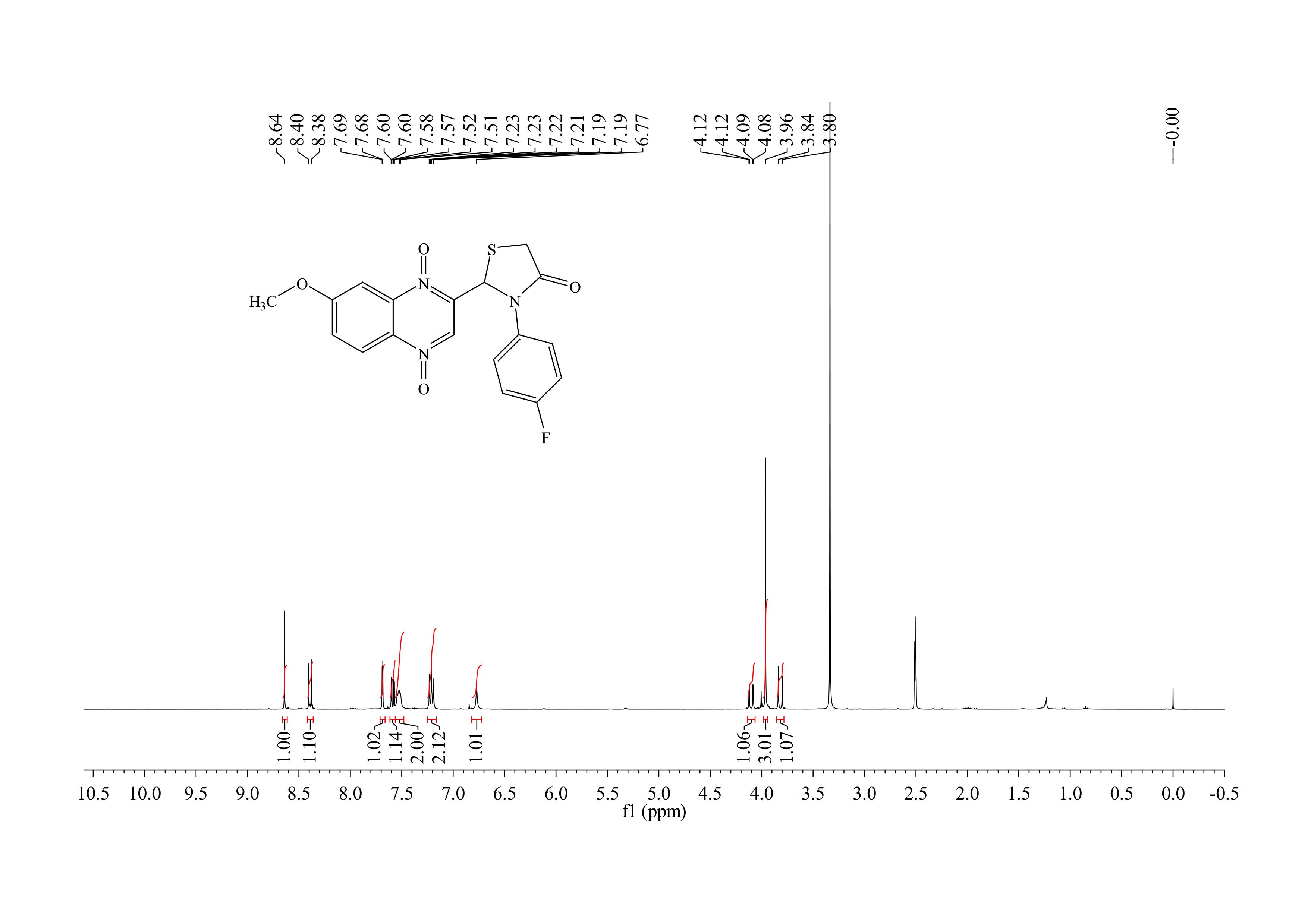


**2o**-^13^C NMR


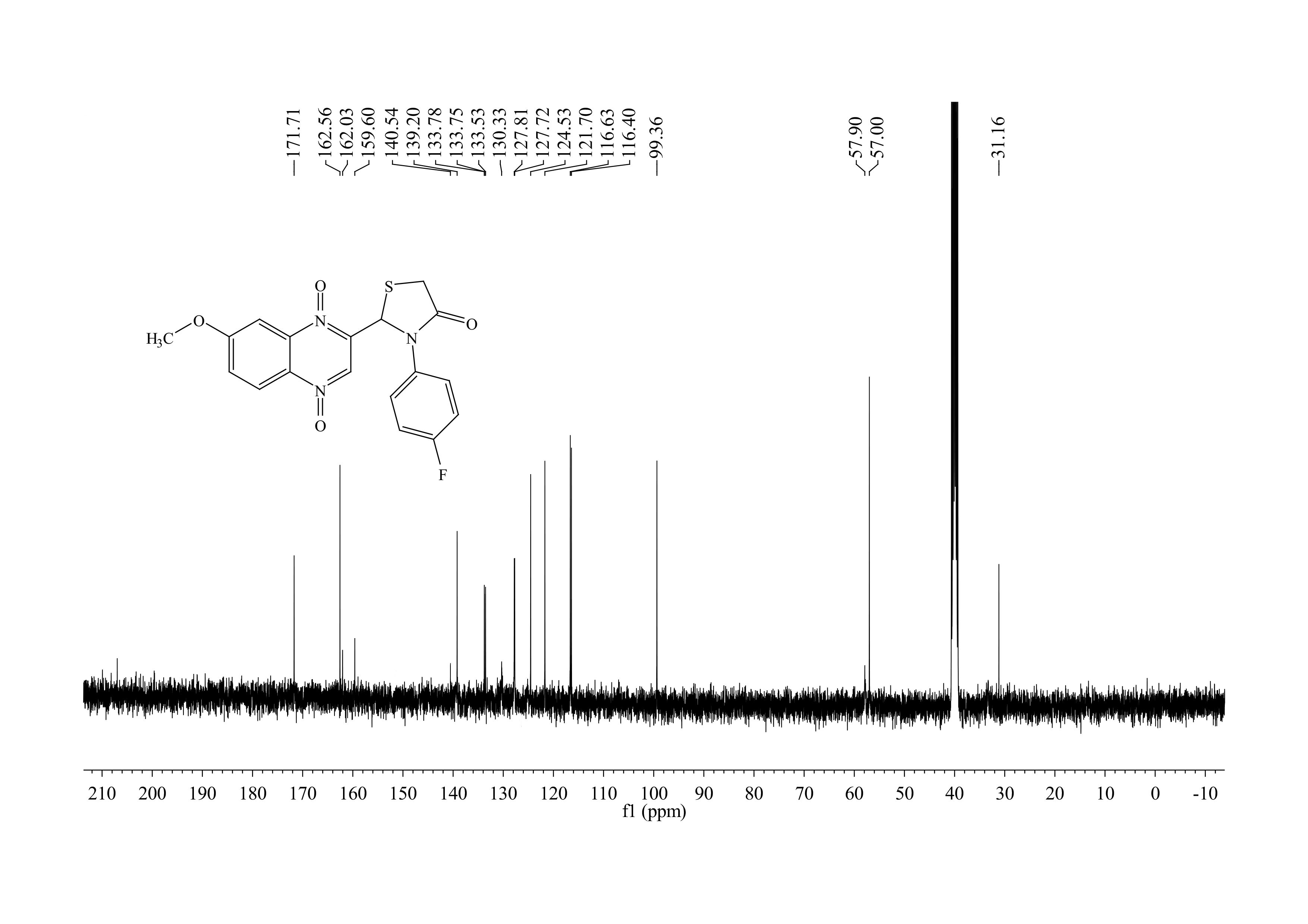


**2p**-^1^H NMR


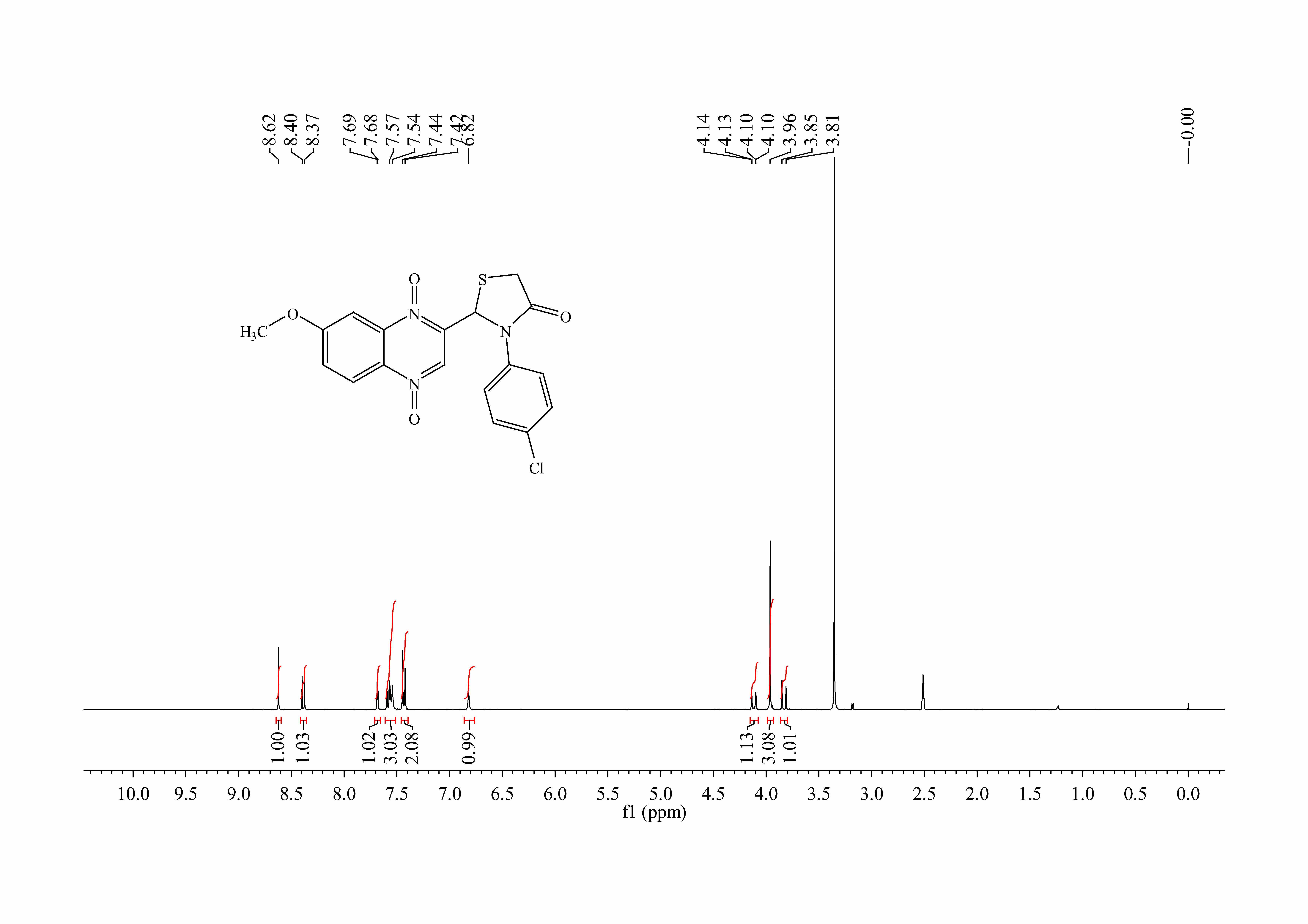


**2p**-^13^C NMR


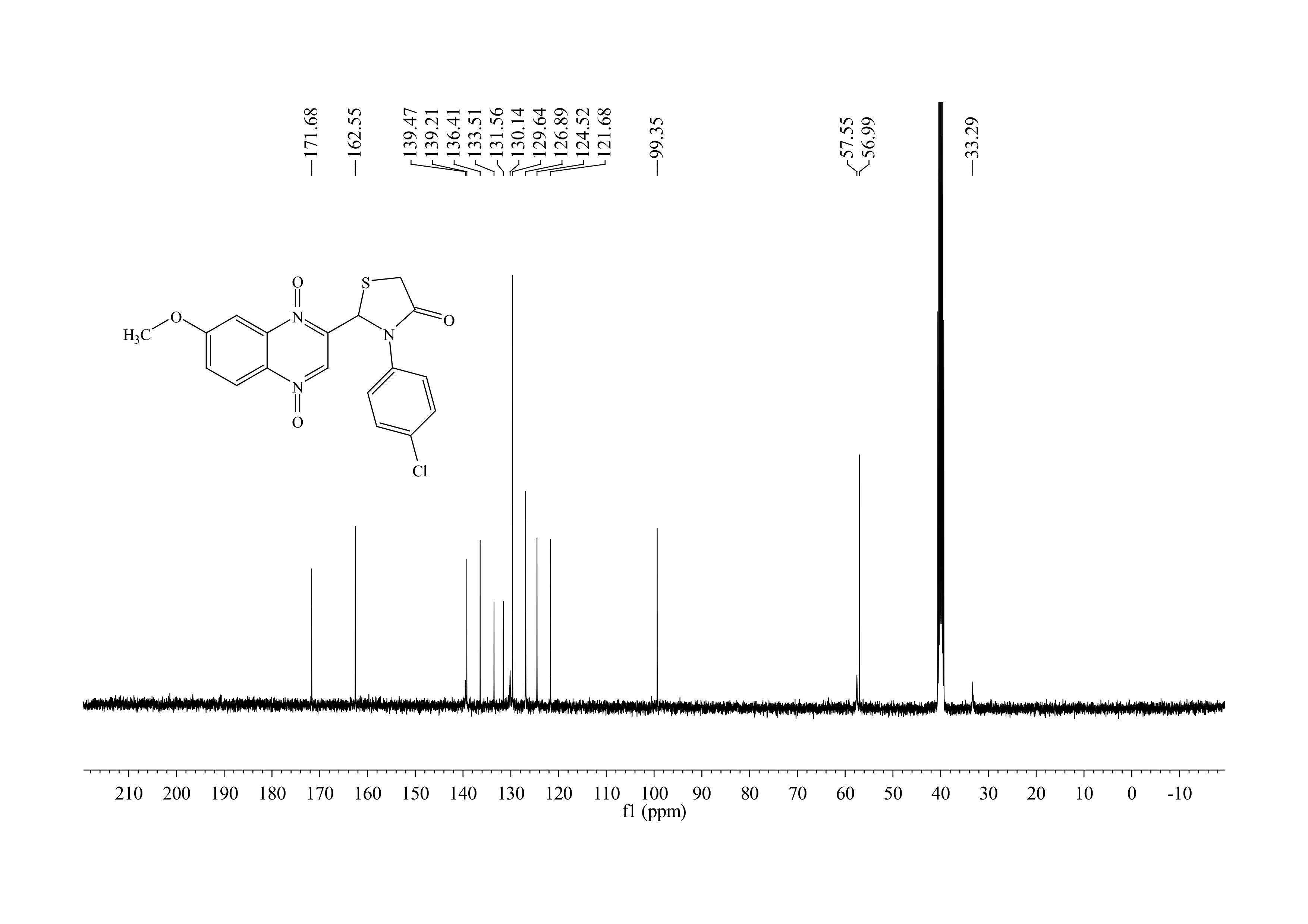


**2q**-^1^H NMR


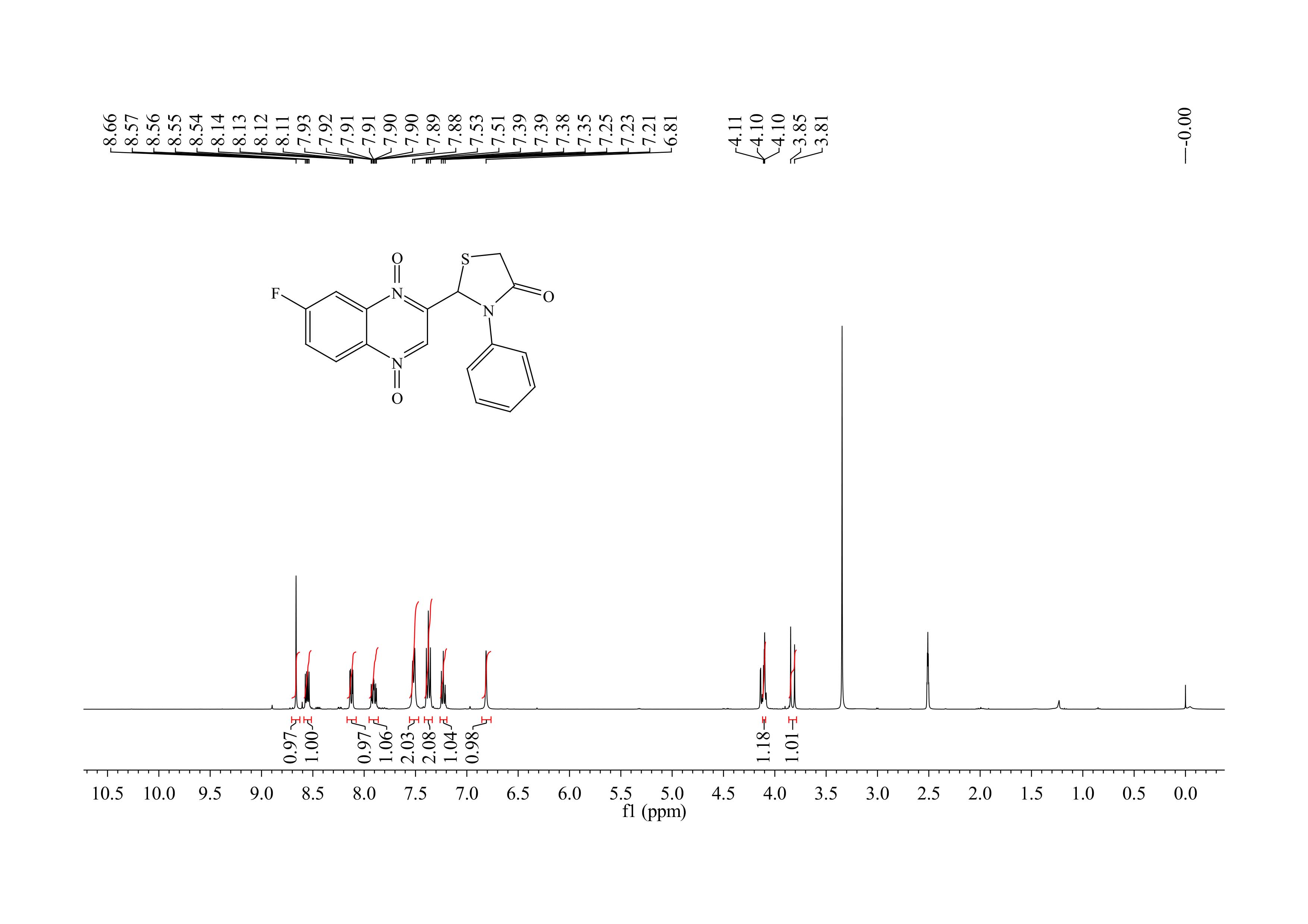


**2q**-^13^C NMR


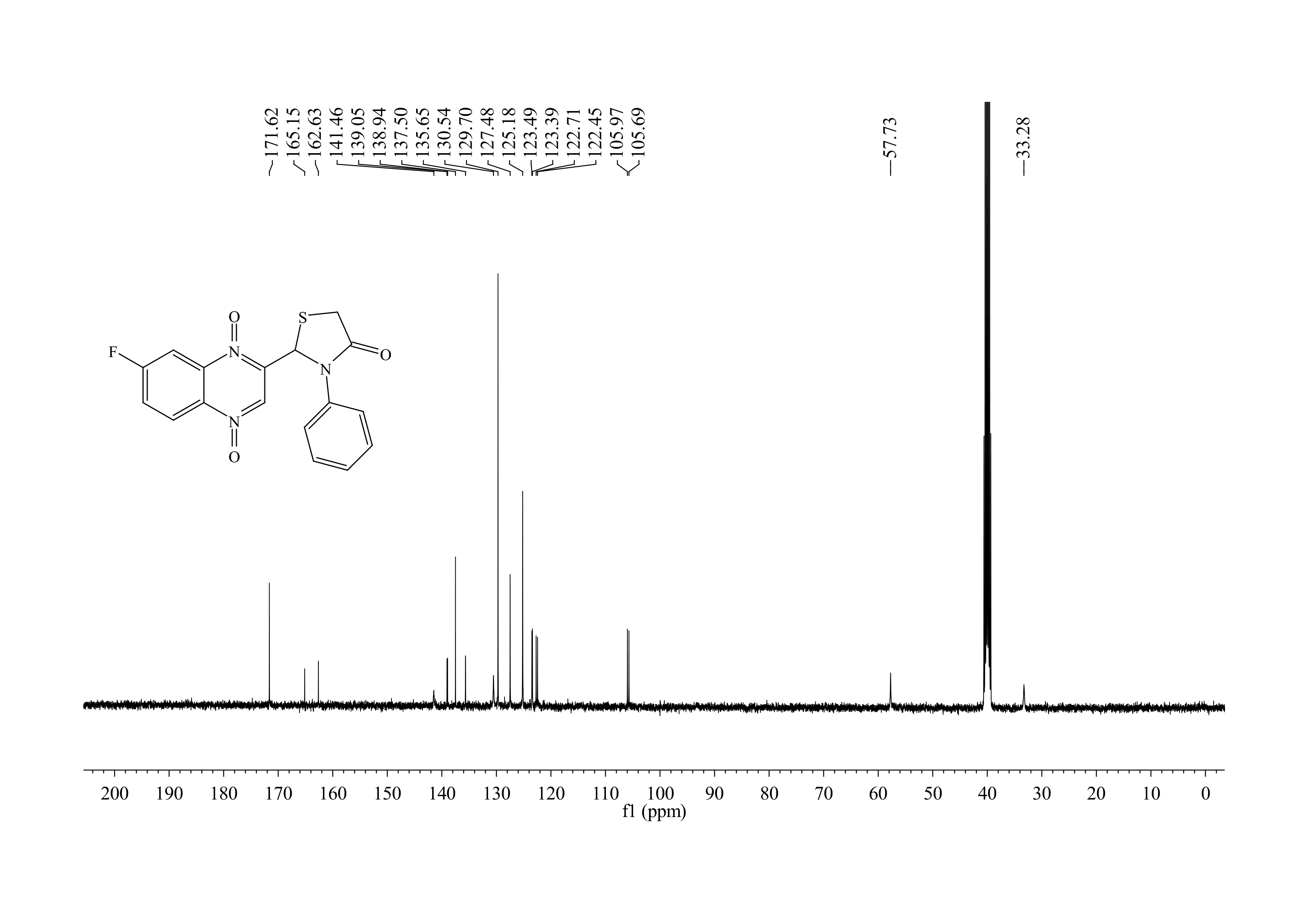


**2r**-^1^H NMR


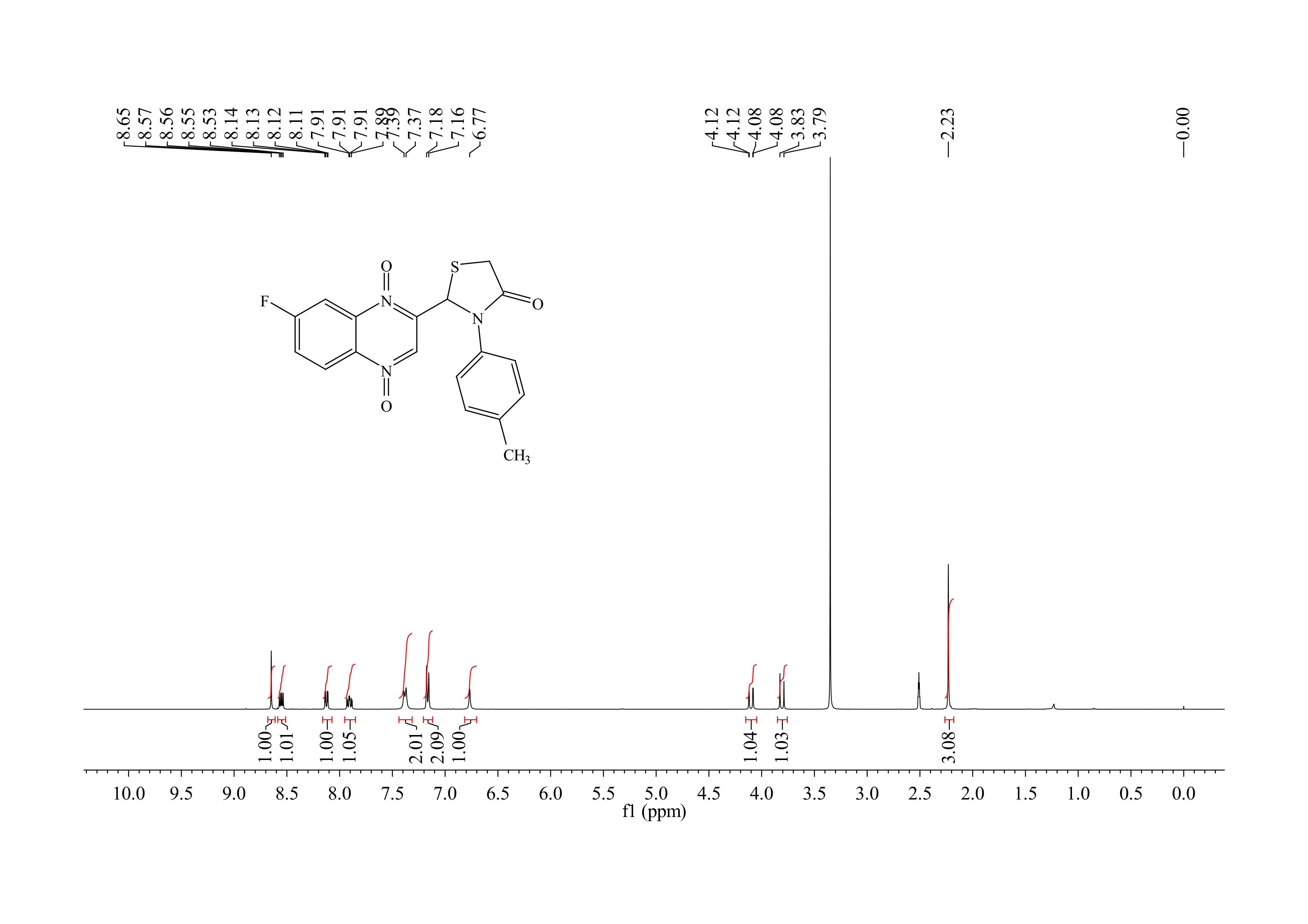


**2r**-^13^C NMR


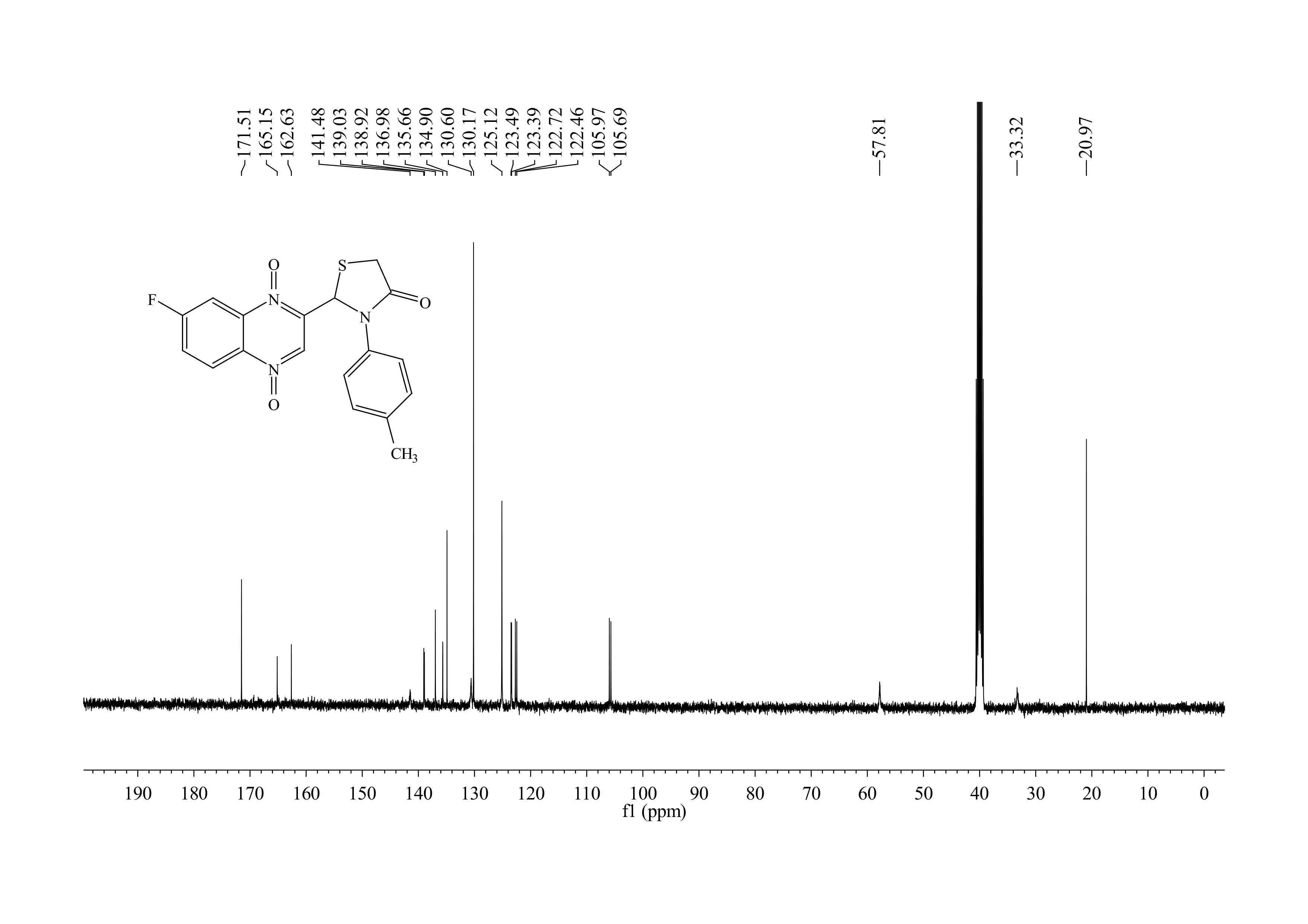


**2s**-^1^H NMR


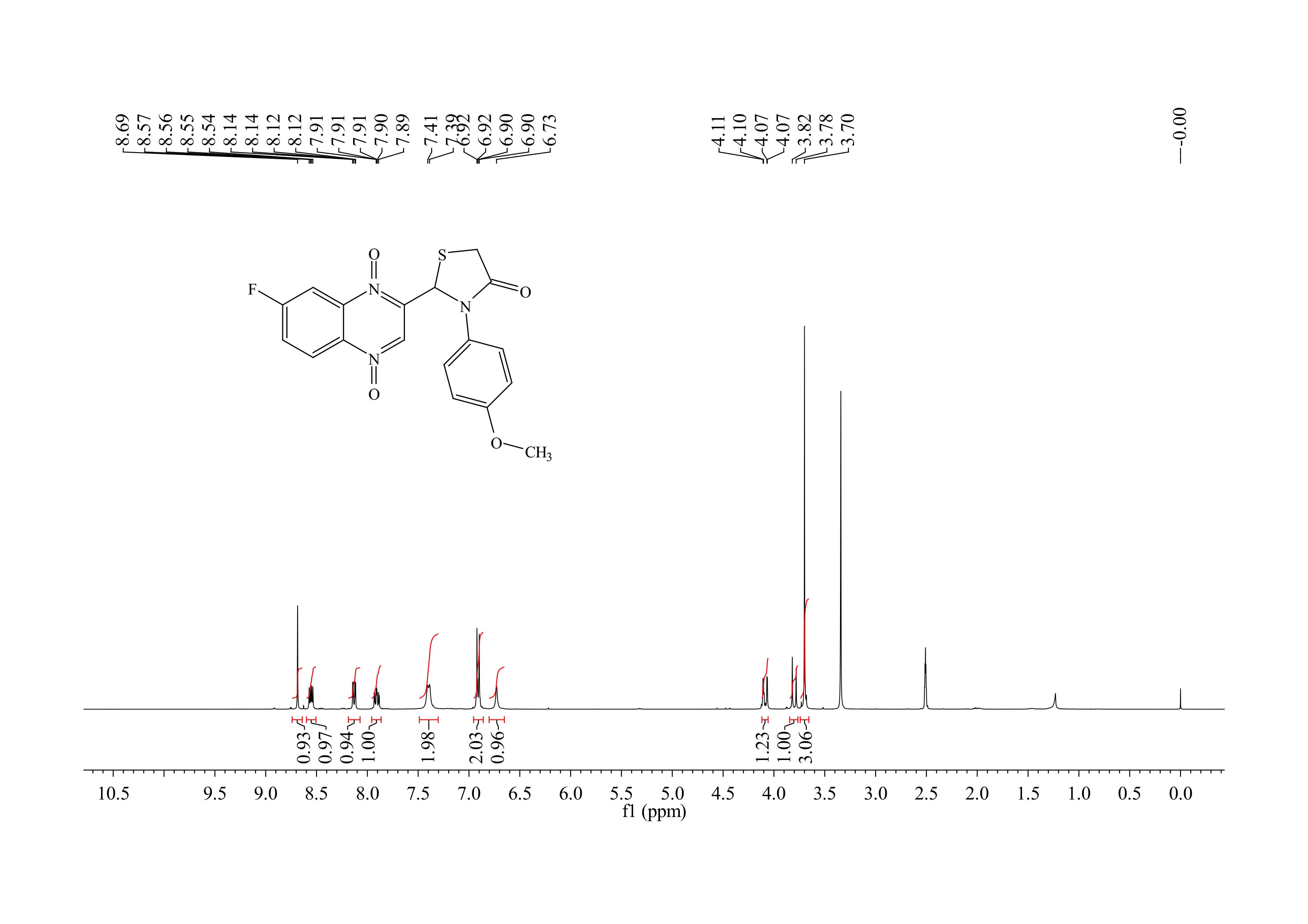


**2s**-^13^C NMR


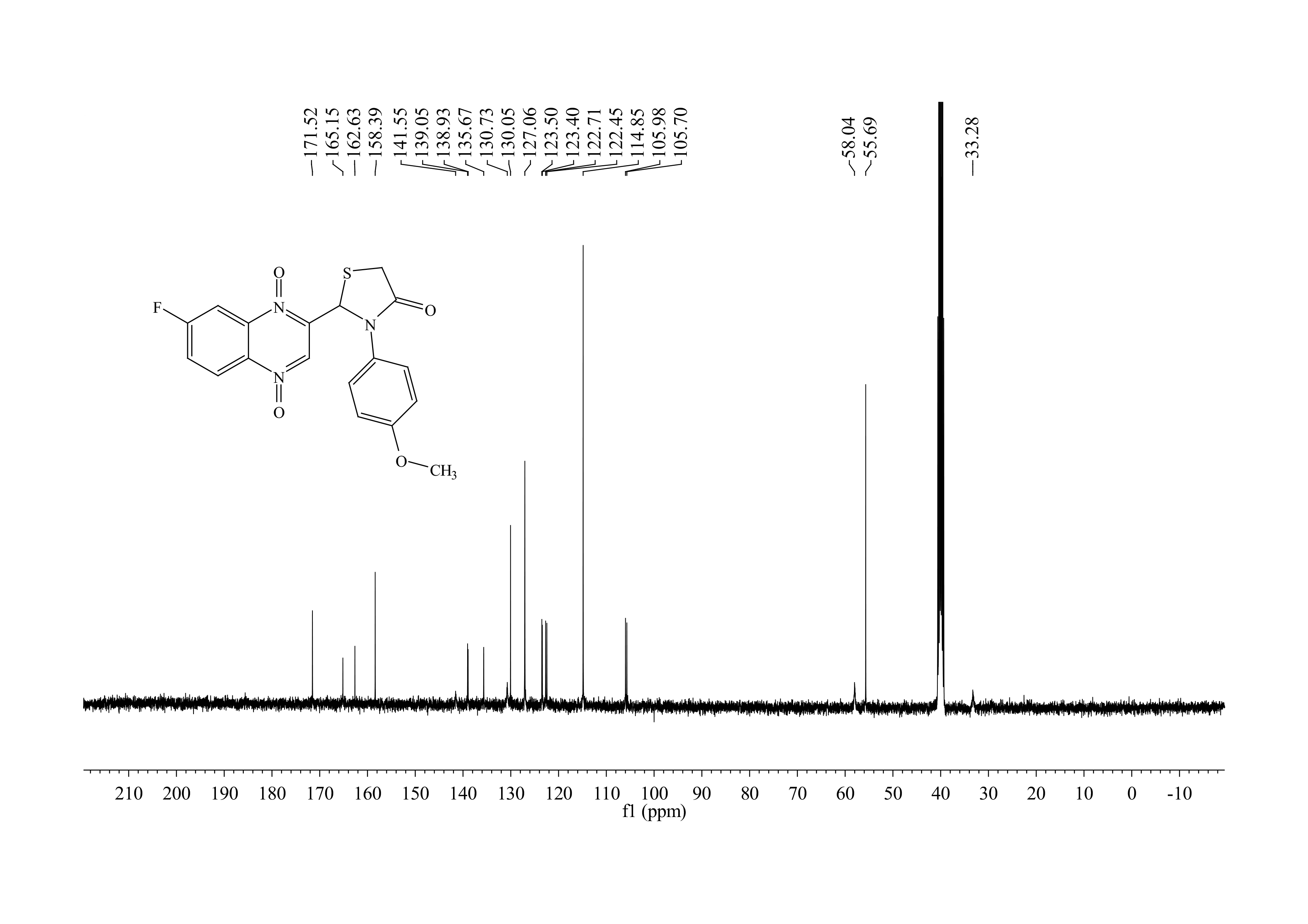


**2t**-^1^H NMR


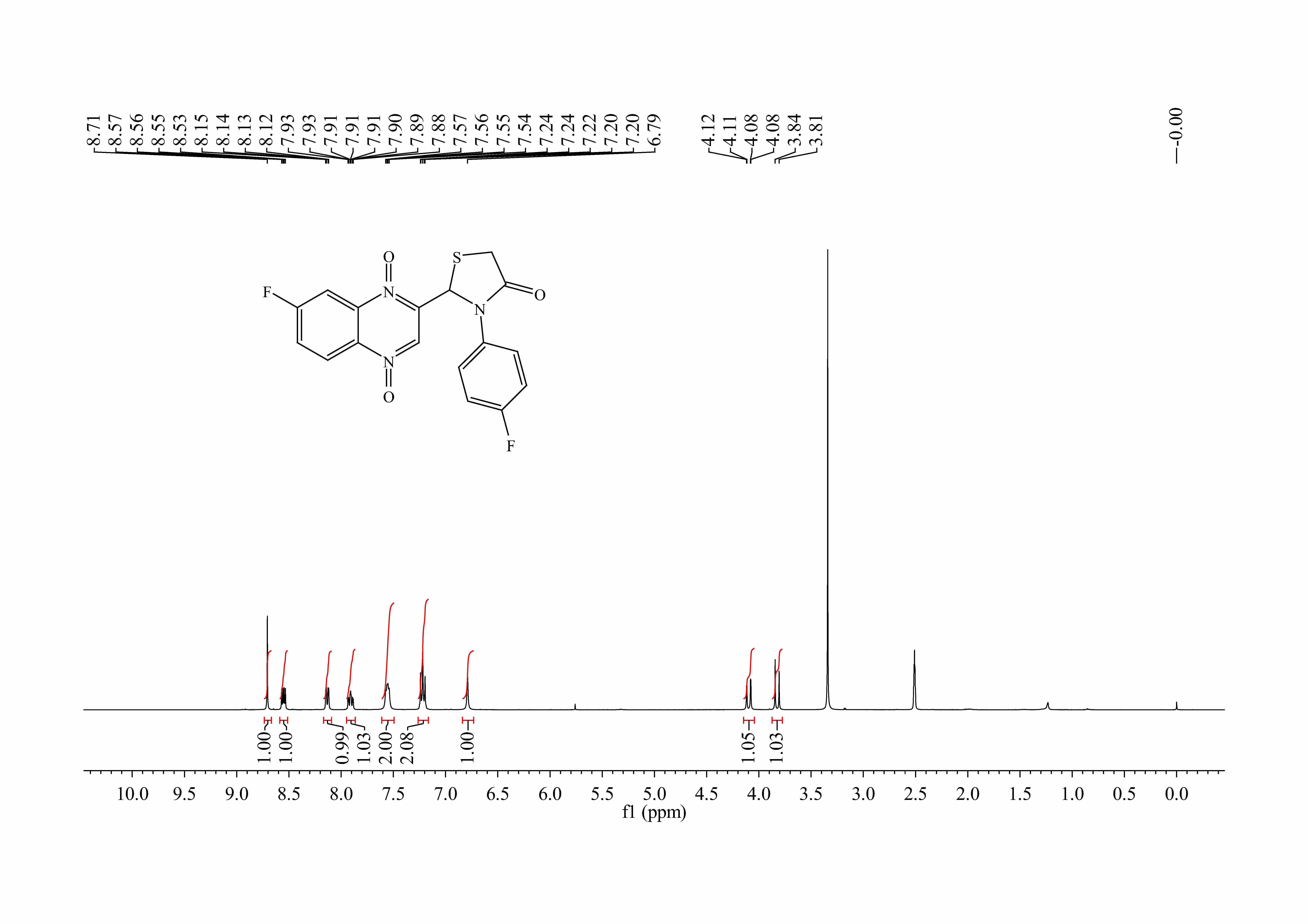


**2t**-^13^C NMR


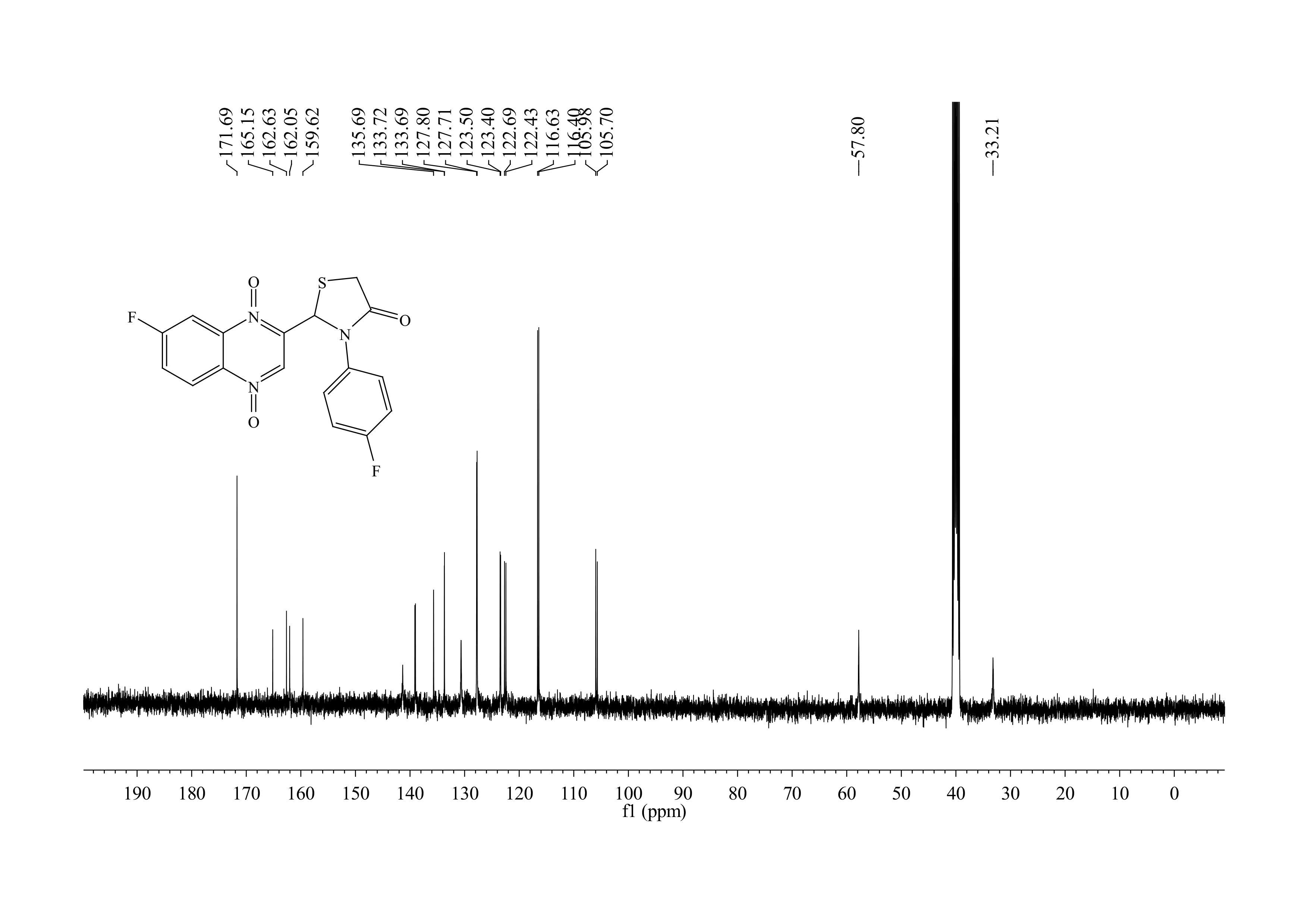


**2u**-^1^H NMR


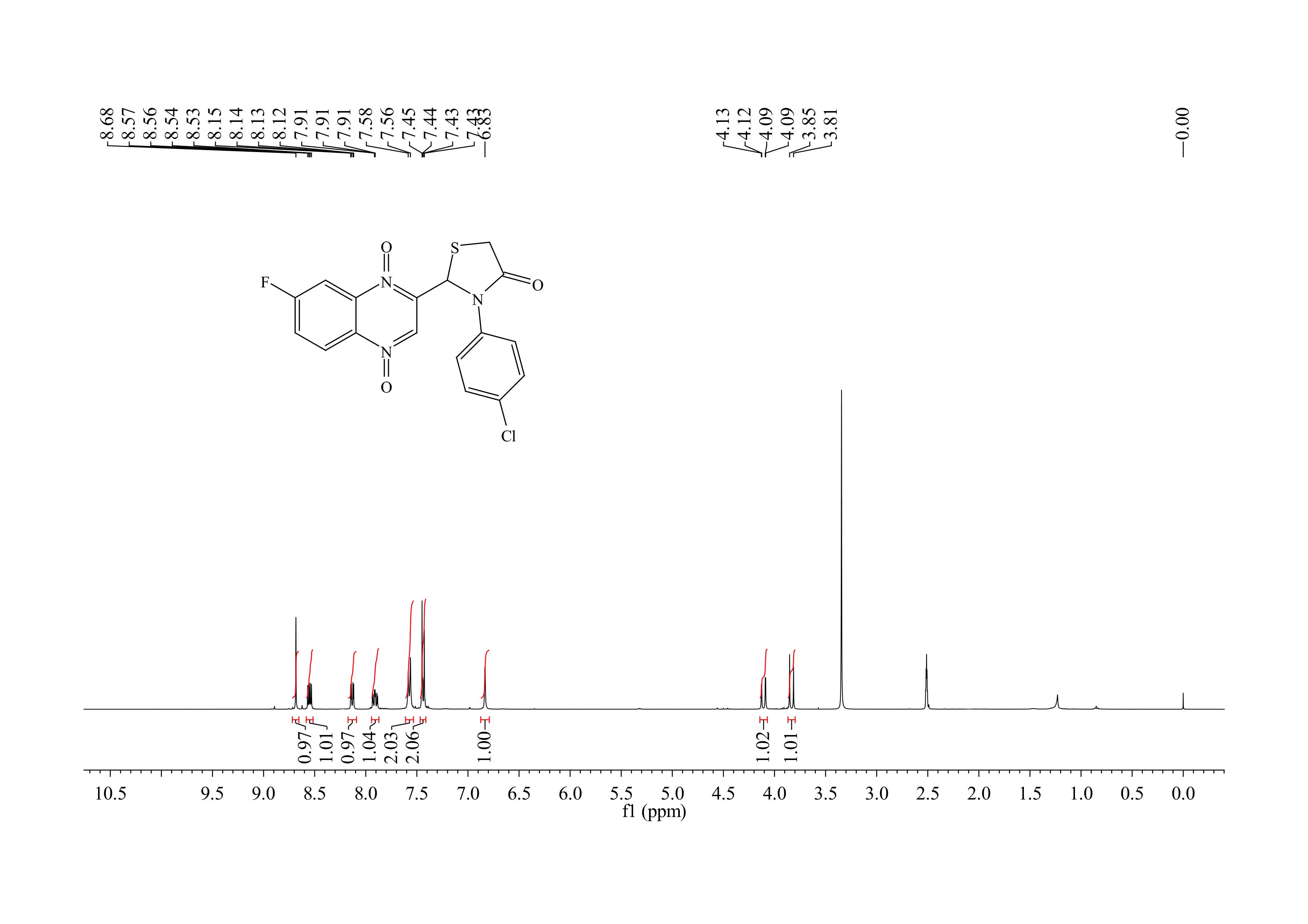


**2u**-^13^C NMR


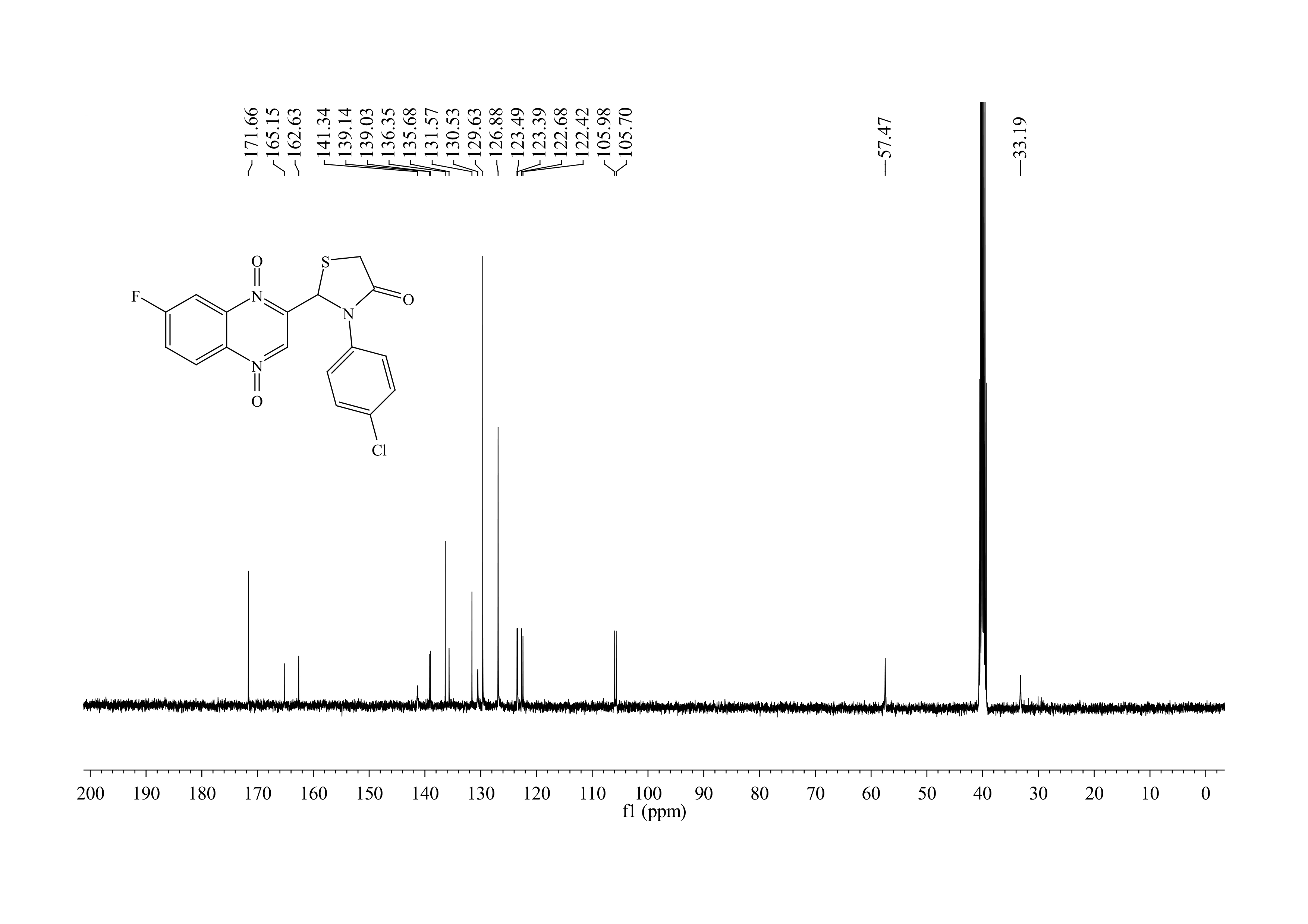


**2v**-^1^H NMR


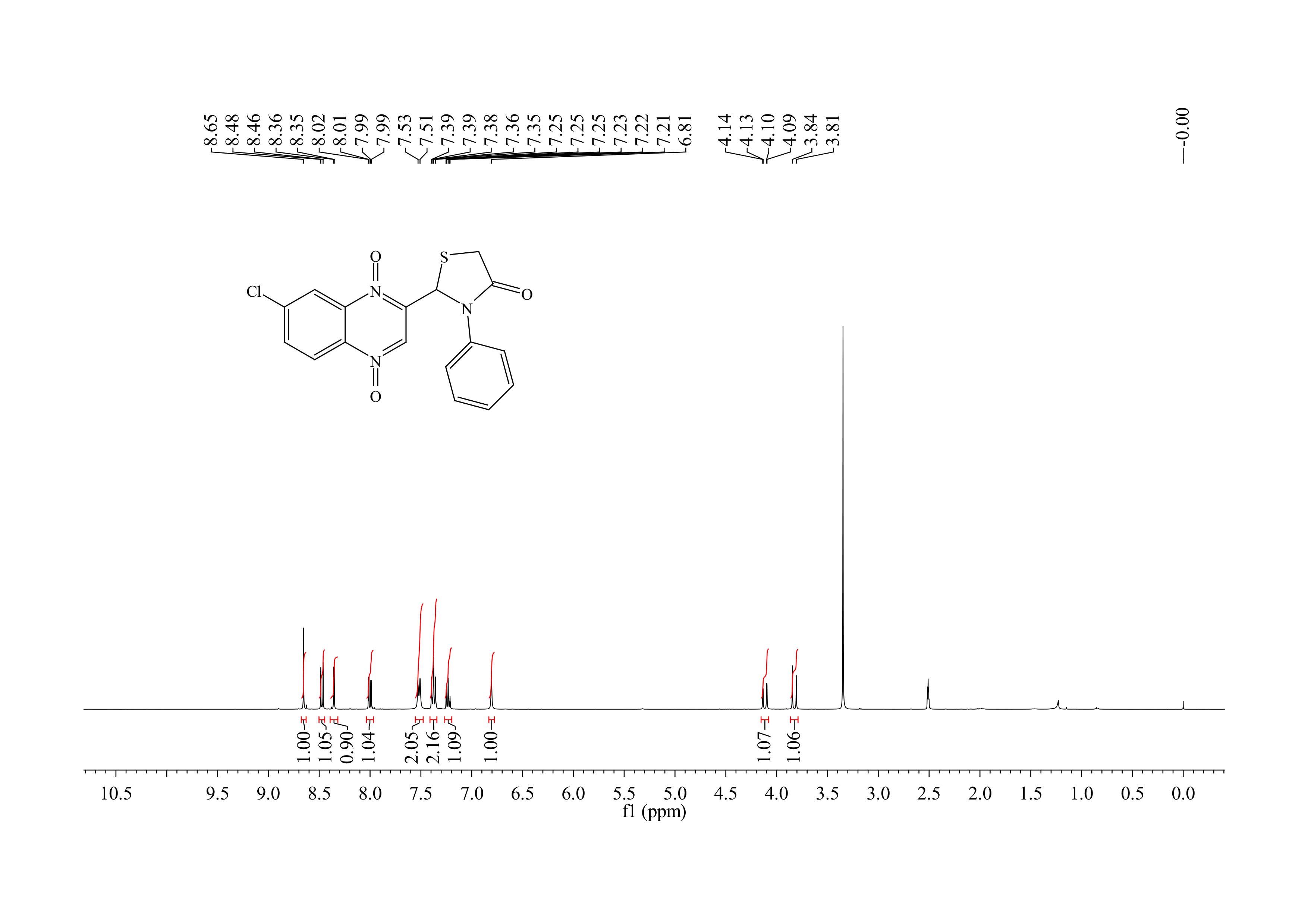


**2v**-^13^C NMR


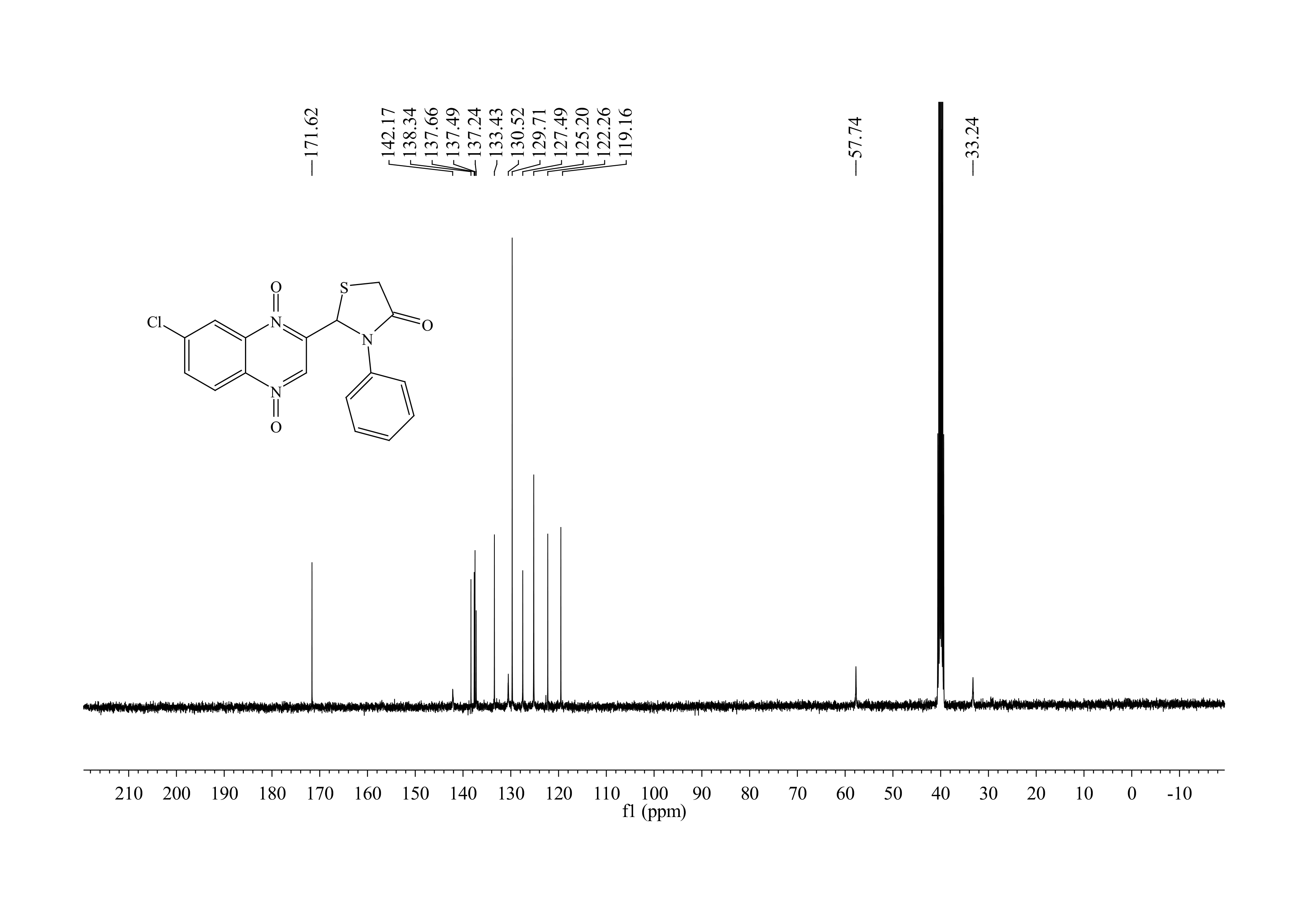


**2w**-^1^H NMR


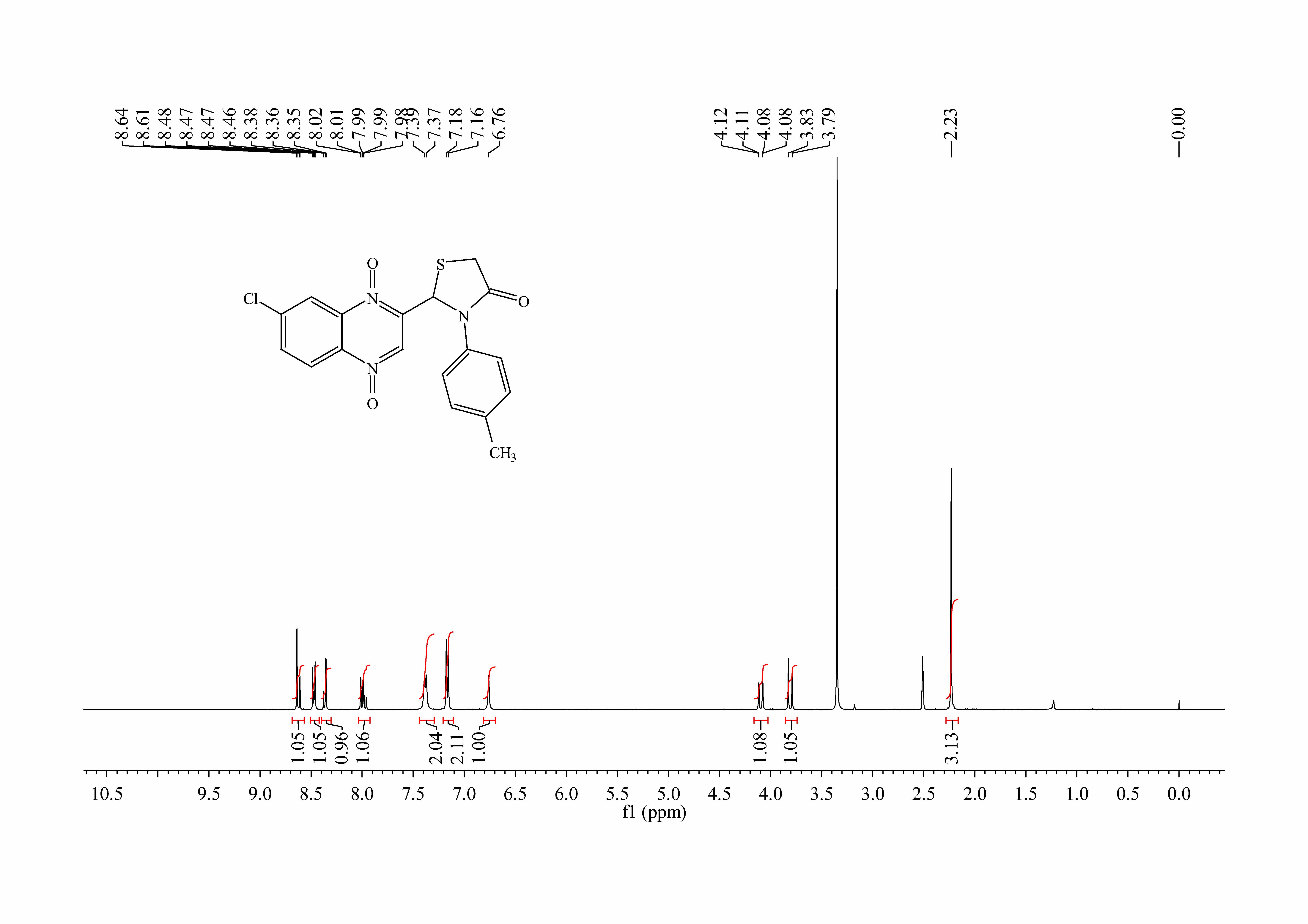


**2w**-^13^C NMR


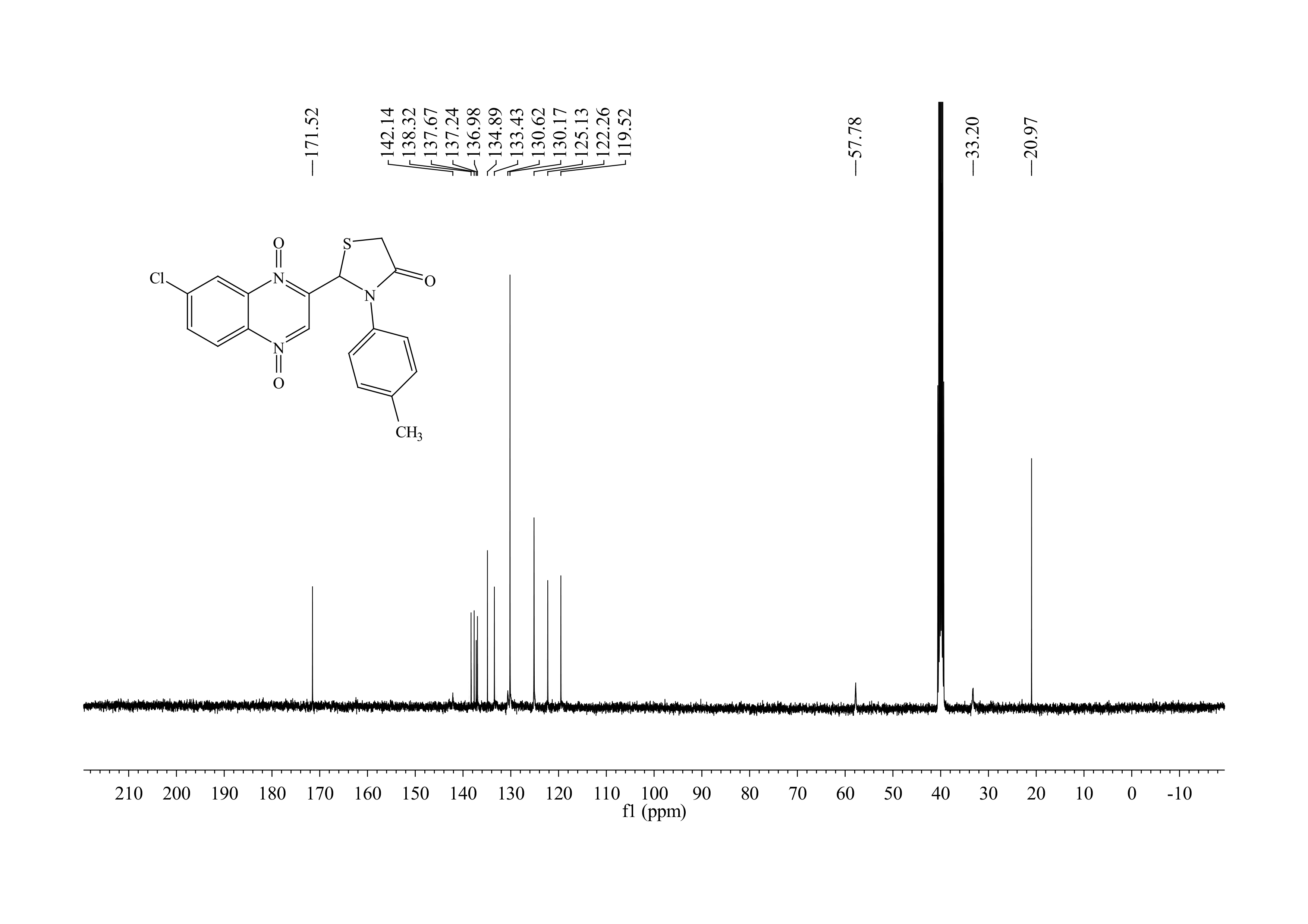


**2x**-^1^H NMR


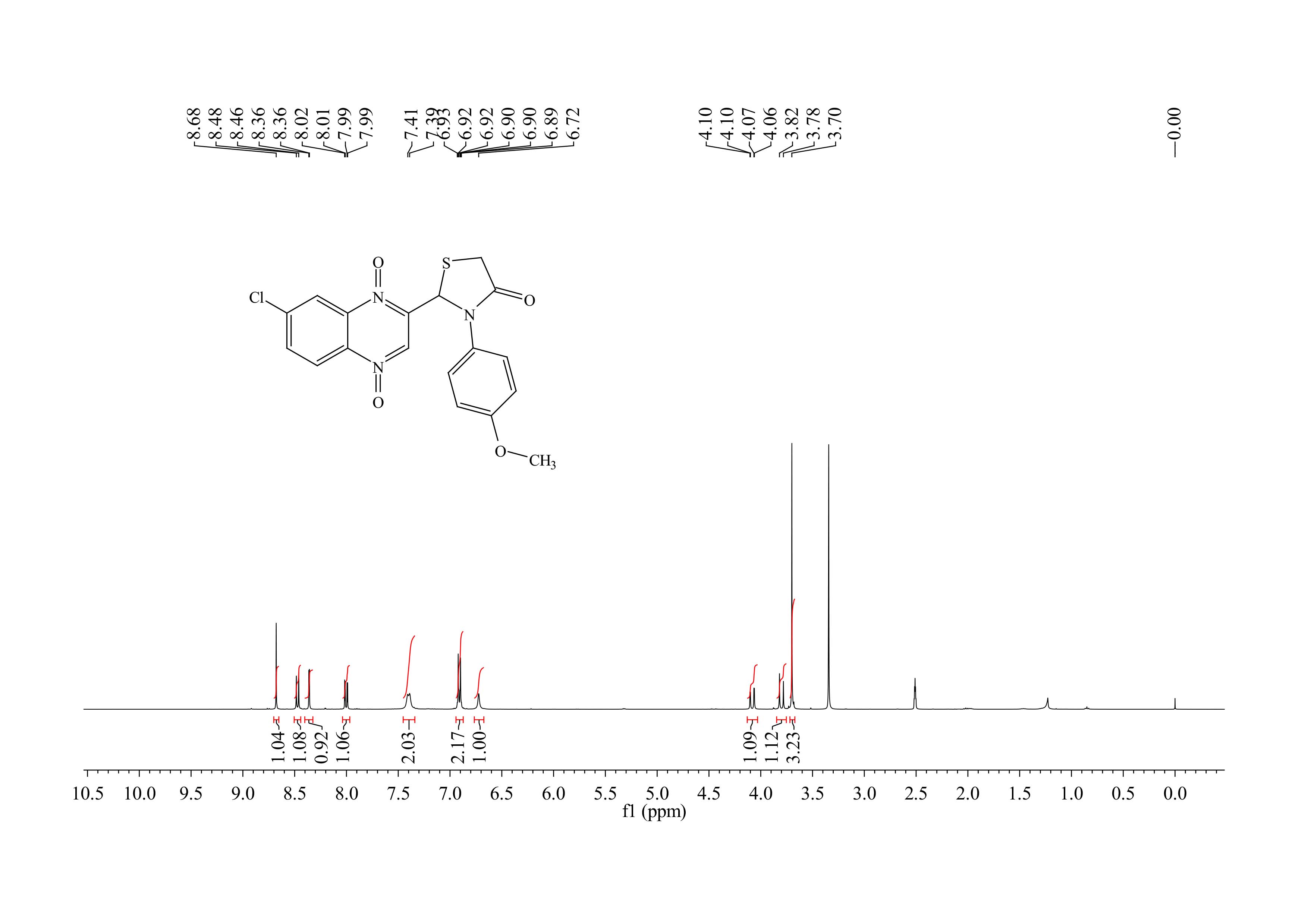


**2x**-^13^C NMR


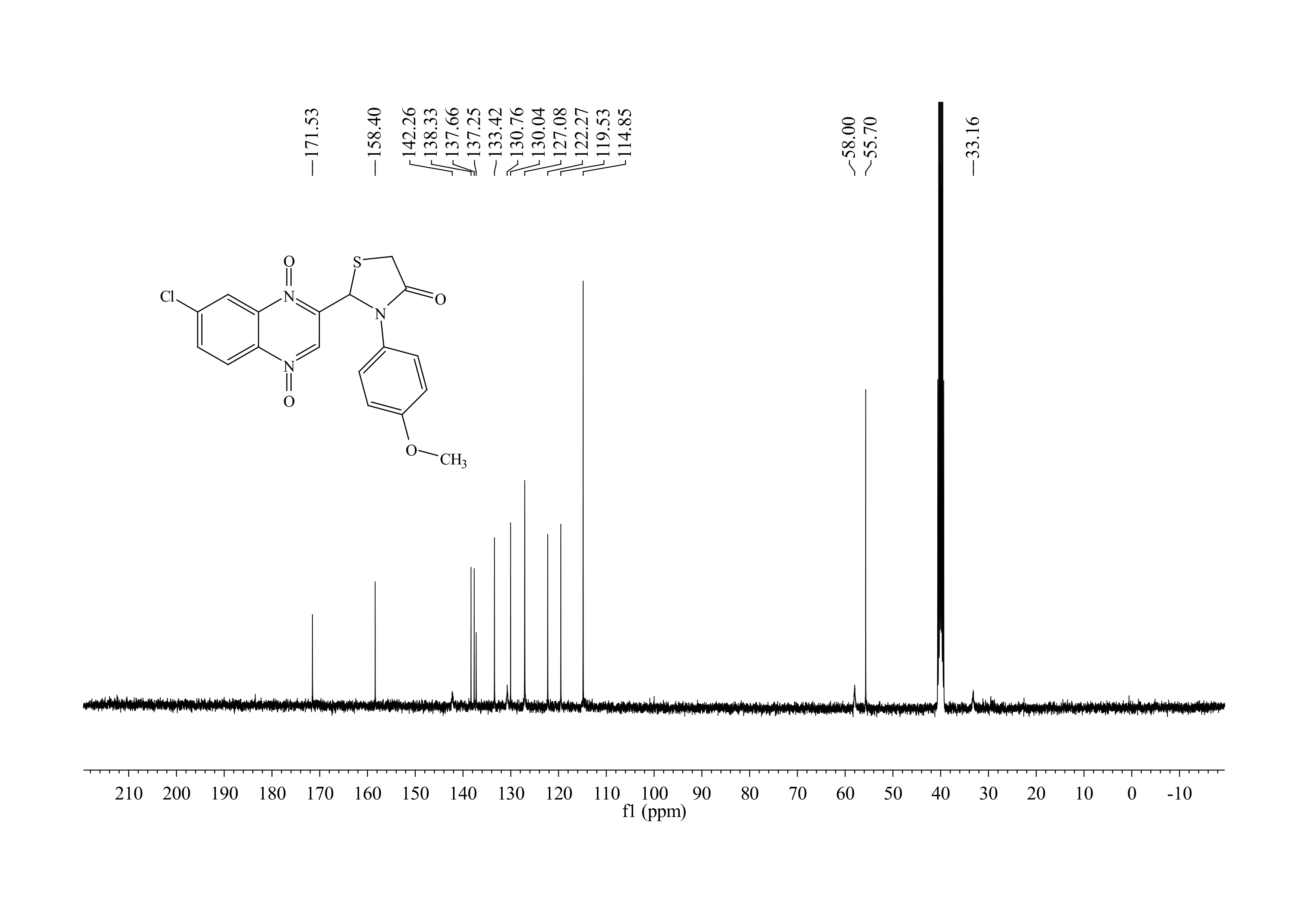


**2y**-^1^H NMR


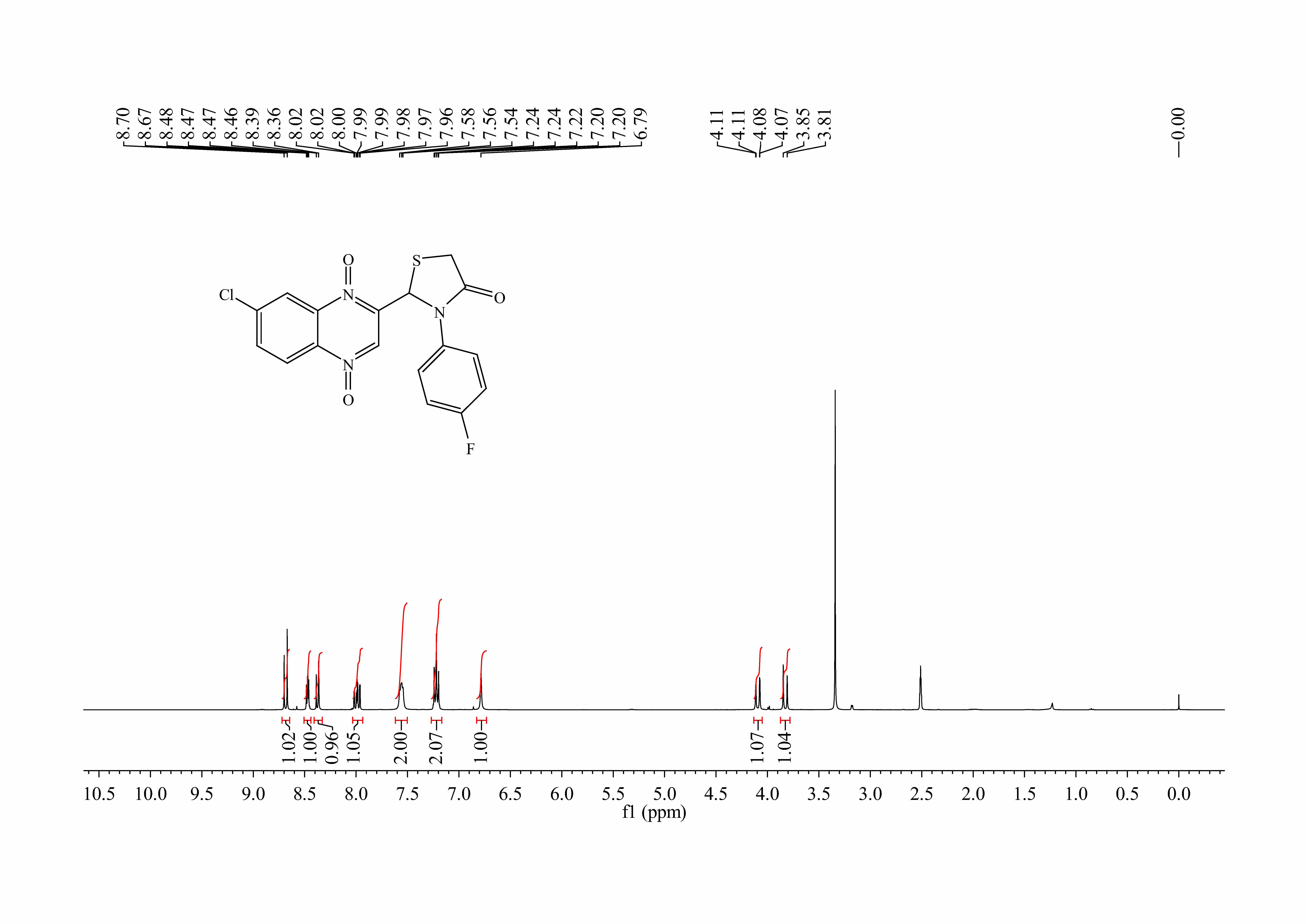


**2y**-^13^C NMR


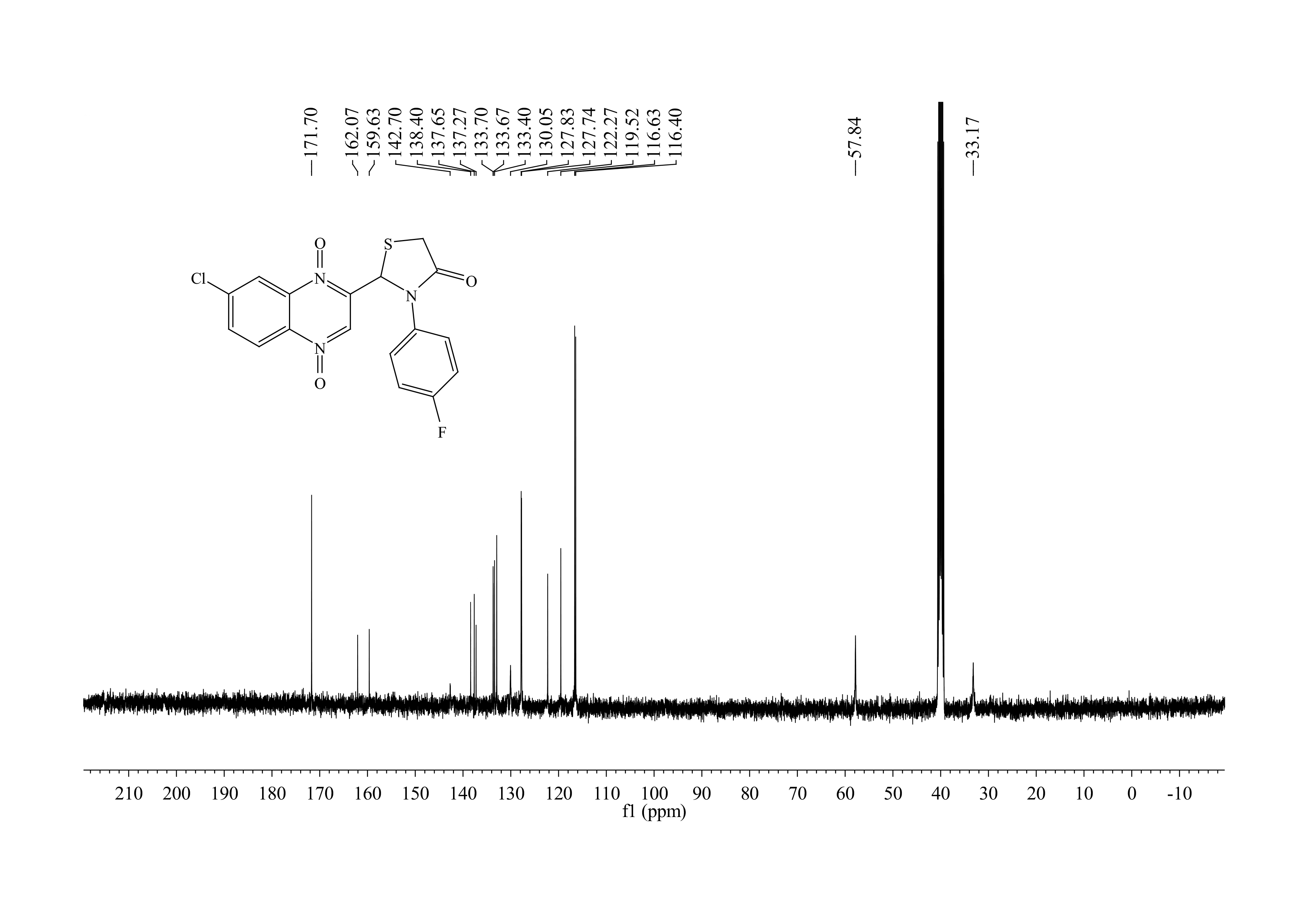


**2z**-^1^H NMR


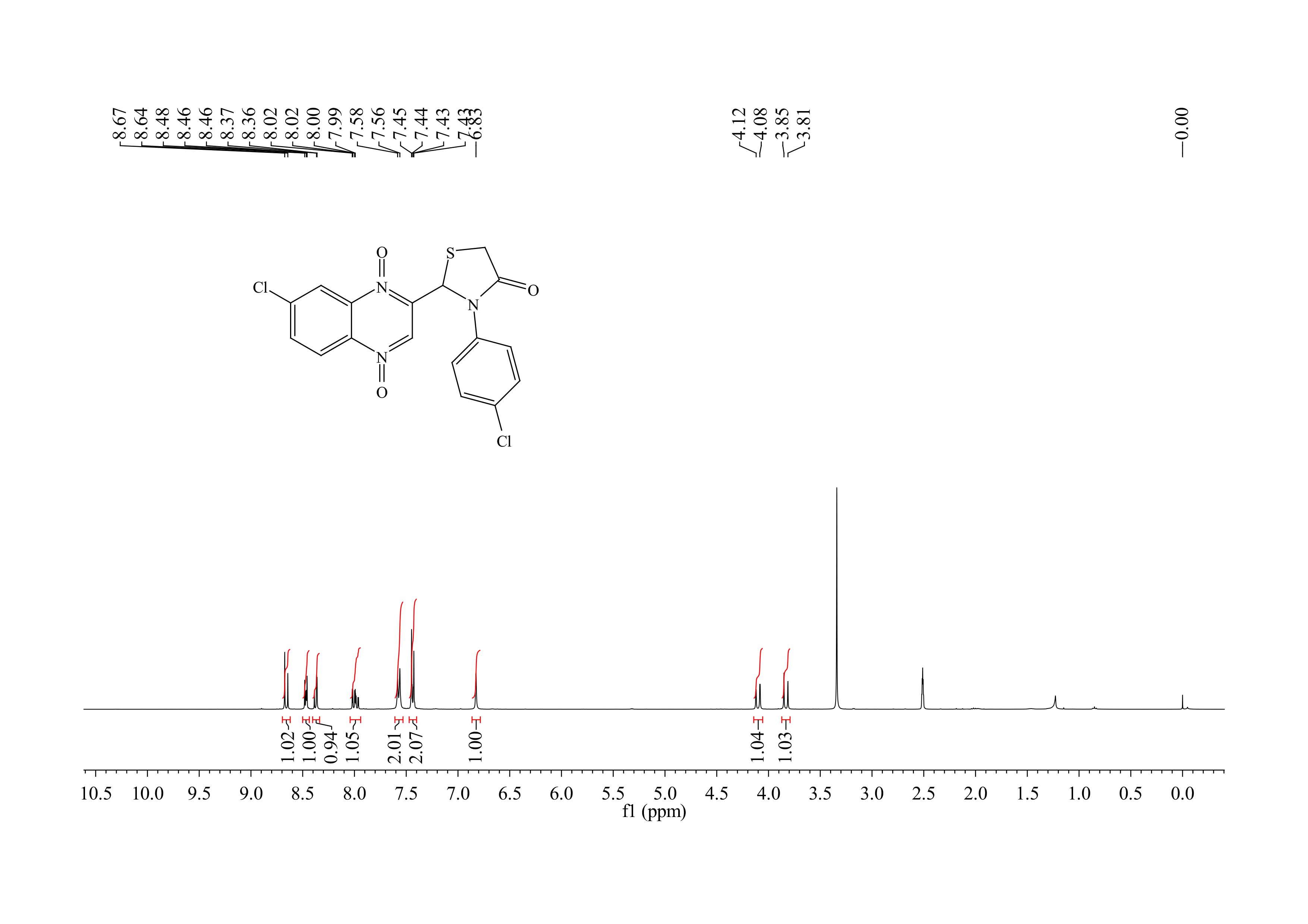


**2z**-^13^C NMR


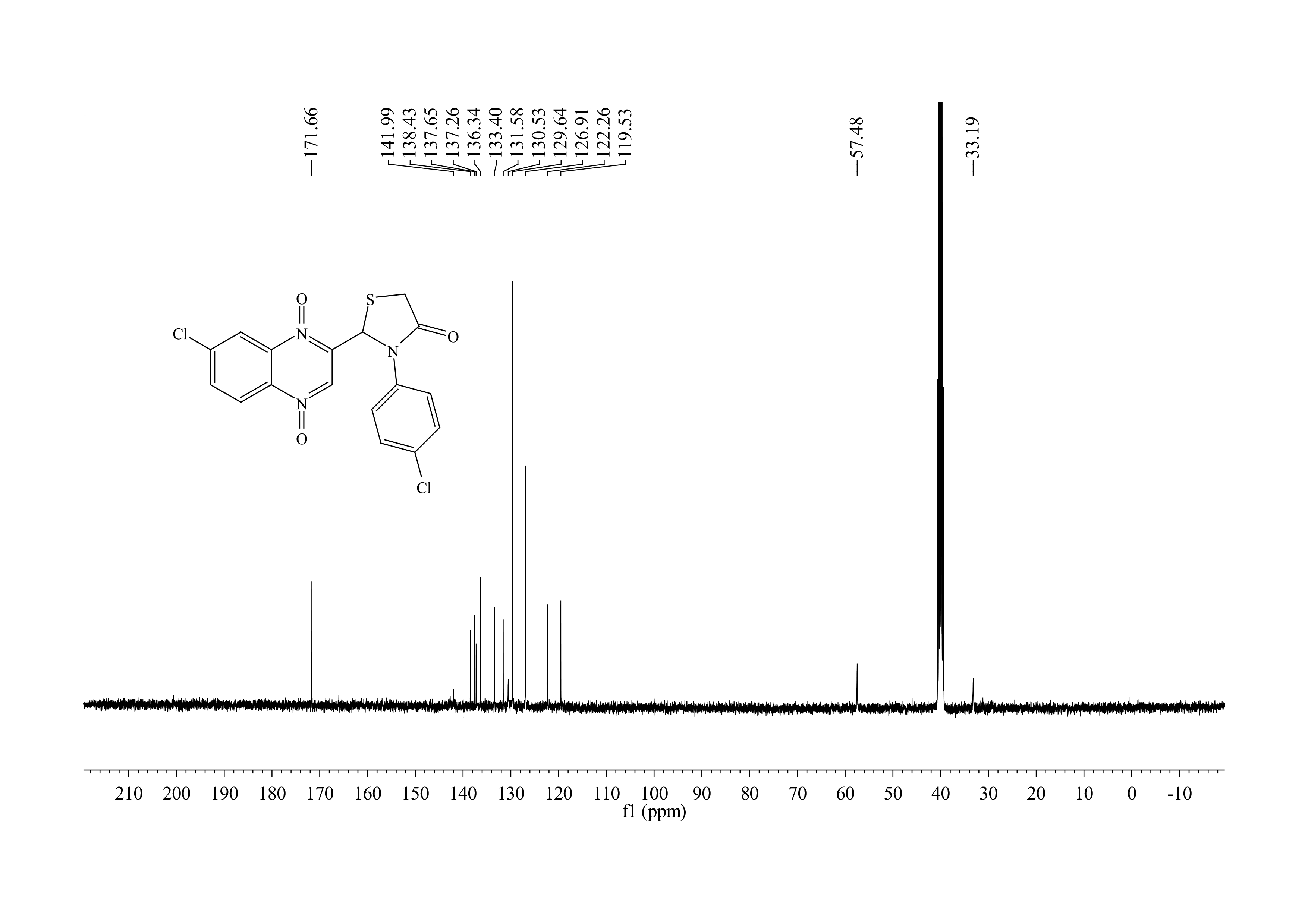

Supplement: Supplementary file 1 [file Data_Sheet_1.doc]
